# Escherichia coli SPFH Membrane Microdomain Proteins HflKC Contribute to Aminoglycoside and Oxidative Stress Tolerance

**DOI:** 10.1128/spectrum.01767-23

**Published:** 2023-06-22

**Authors:** Aimee K. Wessel, Yutaka Yoshii, Alexander Reder, Rym Boudjemaa, Magdalena Szczesna, Jean-Michel Betton, Joaquin Bernal-Bayard, Christophe Beloin, Daniel Lopez, Uwe Völker, Jean-Marc Ghigo

**Affiliations:** a Institut Pasteur, Université de Paris-Cité, CNRS UMR6047, Genetics of Biofilms Laboratory, Paris, France; b Department of Functional Genomics, Interfaculty Institute for Genetics and Functional Genomics, University Medicine Greifswald, Greifswald, Germany; c Abbelight, Cachan, France; d Institut Pasteur, Université de Paris-Cité, UMR UMR6047, Stress adaptation and metabolism in enterobacteria, Paris, France; e Universidad Autonoma de Madrid, Centro Nacional de Biotecnologia, Madrid, Spain; f Centre for Bacteriology Resistance Biology, Imperial College London, London, United Kingdom; g Departamento de Genética, Facultad de Biología, Universidad de Sevilla, Seville, Spain; Navarrabiomed-Universidad Pública de Navarra (UPNA)-Complejo Hospitalario de Navarra (CHN), IdiSNA

**Keywords:** membrane microdomains, SPFH proteins, flotillin, lipid raft, stress tolerance, *Escherichia coli*

## Abstract

Many eukaryotic membrane-dependent functions are often spatially and temporally regulated by membrane microdomains (FMMs), also known as lipid rafts. These domains are enriched in polyisoprenoid lipids and scaffolding proteins belonging to the *s*tomatin, *p*rohibitin, *f*lotillin, and *H*flK/C (SPFH) protein superfamily that was also identified in Gram-positive bacteria. In contrast, little is still known about FMMs in Gram-negative bacteria. In Escherichia coli K-12, 4 SPFH proteins, YqiK, QmcA, HflK, and HflC, were shown to localize in discrete polar or lateral inner membrane locations, raising the possibility that E. coli SPFH proteins could contribute to the assembly of inner membrane FMMs and the regulation of cellular processes. Here, we studied the determinant of the localization of QmcA and HflC and showed that FMM-associated cardiolipin lipid biosynthesis is required for their native localization pattern. Using Biolog phenotypic arrays, we showed that a mutant lacking all SPFH genes displayed increased sensitivity to aminoglycosides and oxidative stress that is due to the absence of HflKC. Our study therefore provides further insights into the contribution of SPFH proteins to stress tolerance in E. coli.

**IMPORTANCE** Eukaryotic cells often segregate physiological processes in cholesterol-rich functional membrane microdomains. These domains are also called lipid rafts and contain proteins of the *s*tomatin, *p*rohibitin, *f*lotillin, and *H*flK/C (SPFH) superfamily, which are also present in prokaryotes but have been mostly studied in Gram-positive bacteria. Here, we showed that the cell localization of the SPFH proteins QmcA and HflKC in the Gram-negative bacterium E. coli is altered in the absence of cardiolipin lipid synthesis. This suggests that cardiolipins contribute to E. coli membrane microdomain assembly. Using a broad phenotypic analysis, we also showed that HflKC contribute to E. coli tolerance to aminoglycosides and oxidative stress. Our study, therefore, provides new insights into the cellular processes associated with SPFH proteins in E. coli.

## INTRODUCTION

In addition to separating the intracellular content of cells from the environment, lipid bilayer membranes also contribute to specialized functions, including cross-membrane transport, enzymatic activity, signaling, and anchoring of cytoskeletal and extracellular structures (1, 2). In eukaryotes, these membrane-dependent functions are often spatially and temporally regulated by functional membrane microdomains (FMMs), also called lipid rafts (3–5). FMMs compartmentalize membrane cellular processes in cholesterol- and sphingolipid-enriched membrane regions formed upon lipid-lipid, lipid-protein, and protein-protein interactions (5–7). A family of membrane proteins called SPFH proteins (for *s*tomatin/*p*rohibitin/*f*lotillin/*H*flK/C) has been shown to localize in eukaryotic lipid rafts and to recruit and provide a stabilizing scaffold to other raft-associated proteins (8–13).

Whereas most prokaryotes lack sphingolipids and cholesterol (14), the Gram-positive bacteria Bacillus subtilis and Staphylococcus aureus can also compartmentalize cellular processes in functional membrane microdomains (FMMs) (14–16). Although whether bacterial FMMs display a distinct lipidic composition still needs to be established, they have been reported to be potentially enriched in polyisoprenoid lipids as well as in cardiolipins at the cell poles (14–16).

FMMs also contain SPFH proteins, including flotillins, and a pool of proteins involved in diverse cellular processes (14, 16, 17). In B. subtilis, flotillins FloT and FloA colocalize in membrane foci and contribute to the assembly of membrane protein complexes (15, 18). Lack of flotillins impairs biofilm formation, sporulation, protease secretion, motility, and natural competence, indicating that the formation of FMMs also plays critical cellular roles in B. subtilis (15, 18–22).

SPFH proteins are also present in Gram-negative bacteria, and Escherichia coli K-12 even possesses four genes, *yqiK*, *qmcA*, *hflK*, and *hflC*, which encode proteins with an SPFH domain and an N-terminal transmembrane (TM) segment (23). QmcA and YqiK are predicted to face the cytoplasmic compartment, while HflK and HflC are predicted to be exposed in the periplasm, forming the HflKC complex negatively regulating the protease activity of FtsH against membrane proteins (24–27). Fluorescent microscopy also showed that E. coli SPFH proteins HflC and QmcA are localized in discrete polar or lateral membrane foci (28), raising the possibility that E. coli SPFH proteins could localize in inner membrane FMMs and regulate specific cellular processes (29). However, apart from the functional and structural description of HflKC as a regulator of the FtsH membrane protease (24, 27, 30) and a recent study suggesting that YqiK is involved in cell motility and resistance to ampicillin (31), the functions of FMMs in E. coli and other Gram-negative bacteria are still poorly understood.

In this study, we used fluorescent and superresolution microscopy to perform a detailed analysis of QmcA and HflC membrane localization signals. We then showed that the integrity of QmcA and HflC protein domains is required for their inner membrane localization and that the lack of cardiolipin and isoprenoid lipids known to associate with FMMs alters their localization. Moreover, using single and multiple SPFH gene mutants, we showed that HflKC SPFH proteins contribute to aminoglycoside and oxidative stress resistance. Our study therefore provides new insights into the determinants of cellular localization and the function associated with E. coli SPFH proteins.

## RESULTS

### Chromosomal E. coli SPFH fluorescent fusion proteins show distinct localization patterns.

**FIG 1:**
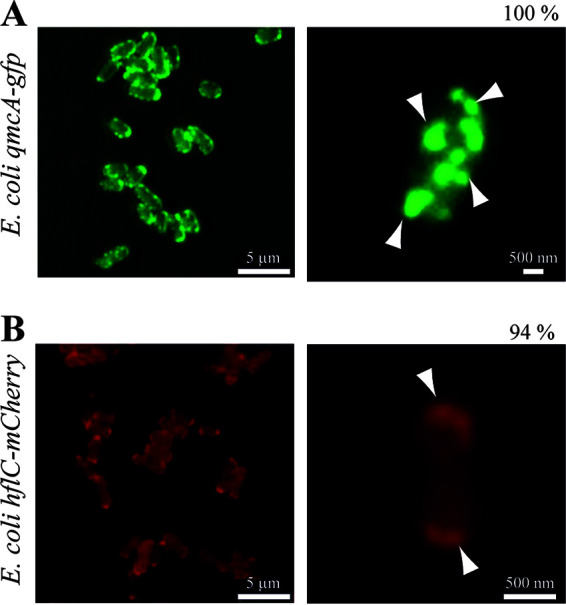
Cell localization patterns of HflC and QmcA. Epifluorescence microscopy of cells expressing QmcA-GFP (A) and HflC-mCherry (B). Representative images are shown. Percentages indicate the frequencies of cells showing localization foci. Arrowheads indicate polar or punctate localization foci. Scales are indicated as white bars.

**FIG 2:**
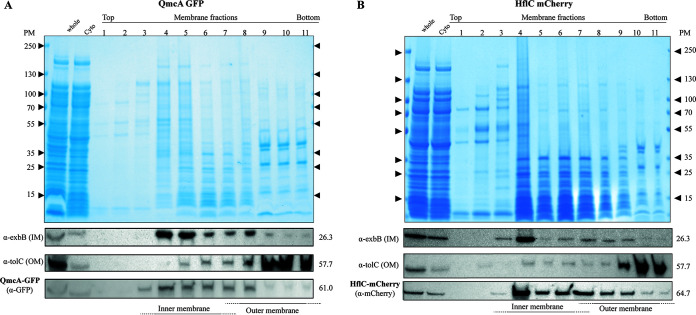
QmcA and HflC localize to the inner membrane. SDS-PAGE and immunodetection analyses of whole-cell extracts, cytosolic fractions, and inner membrane (IM) or outer membrane (OM) fractions prepared from cells expressing QmcA-GFP (A) and HflC-mCherry (B). Anti-GFP and anti-mCherry antibodies were used to detect the presence of QmcA-GFP and HflC-mCherry, respectively. An anti-ExbB antibody was used to detect the inner membrane (IM) marker ExbB, and anti-TolC antibodies were used to detect the outer membrane (OM) marker TolC.

To investigate the determinant of cell localization of E. coli SPFH proteins, we first tagged YqiK and QmcA, the C termini of which are predicted to be in the cytoplasm (26), with a C-terminal monomeric superfolder green fluorescent protein (msfGFP). We then tagged HflC and HflK, the C termini of which are predicted to be in the periplasm (26), with the C-terminal monomeric red fluorescent protein mCherry. All these fusions were expressed under their own promoter from their native chromosomal location (see Fig. S1 in the supplemental material). Epifluorescence and superresolution microscopy confirmed the previously reported polar localization of HflK and HflC (28), with 94% and 91% polar localization patterns for HflC-mCherry and HflK-mCherry, respectively (*n* = 150) (Fig. 1B and Fig. S2). In contrast, C-terminally tagged QmcA-GFP showed punctate foci distributed throughout the cell body, with 96% of the cells harboring 5 foci or more (*n* = 150) (Fig. 1A). However, we could not detect YqiK-GFP, possibly due to its low native chromosomal expression level. We then used anti-GFP or mCherry antibodies to perform immunodetection on cytoplasmic as well as inner and outer membrane fractions of E. coli strains expressing either HflC-mCherry or QmcA-GFP. In agreement with previous results (26, 29), both fusion proteins were detected in the inner membrane fraction and showed minimal degradation profiles (Fig. 2 and Fig. S3 and S4). Moreover, the functionality of the HflKC fusion could be demonstrated (see below).

### Domain swap analysis shows that protein integrity is essential for QmcA-GFP and HflC-mCherry localization.

**FIG 3:**
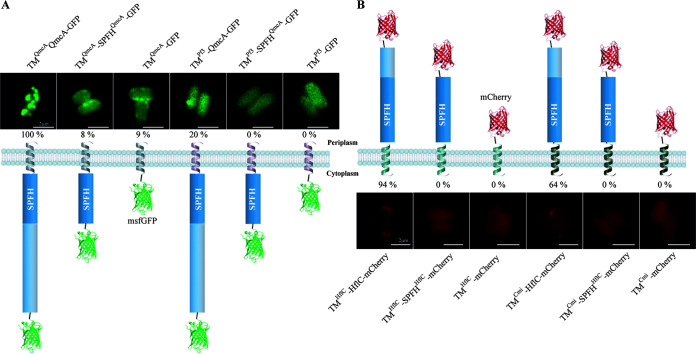
The localization pattern and membrane topology of full-length, domain-swapped, or truncated versions of QmcA and HflC. (A) GFP fusion derivatives of QmcA; (B) mCherry fusion derivatives of HflC. The representative images are shown for each strain with the frequencies of cells showing punctate (A) or polar (B) localization. In panel A, second panel from left, the control WT QmcA-GFP is renamed TM^QmcA^-SPFH^QmcA^-GFP, and the fluorescence microscopy image corresponding to its localization is therefore a duplicate of the one presented in Fig. 1A. Similarly, in panel B, second panel from left, the control WT HflC-mCherry is renamed TM^HflC^-SPFH^HflC^-mCherry, and the fluorescence microscopy image corresponding to its localization is therefore a duplicate of the one presented in Fig. 1B. In membrane topology, helical structures represent transmembrane (TM) domains; silver, native TM domain of QmcA; pink, Pf3 domain; green, native TM domain of HflC; black, Cmi domain. Bars, 2 μm.

To identify HflC and QmcA membrane localization signals, we constructed multiple fluorescently tagged truncated versions of both proteins. We tagged with msfGFP a QmcA protein reduced to its transmembrane region and SPFH domain (QmcA-GFP is therefore now named TM^QmcA^-SPFH^QmcA^-GFP in Fig. 3) and, separately, one reduced to the QmcA transmembrane region only (TM^QmcA^-GFP) (Fig. 3A). To test the role of the QmcA transmembrane region, we also swapped TM^QmcA^ in the three constructs with the single-spanning TM domain of the phage coat protein Pf3 (TM^Pf3^), which orients subsequent amino acids to the cytosol (32) (Fig. 3A). Similarly, in addition to the full-length HflC-mCherry, we tagged with mCherry the HflC transmembrane region and SPFH domain (HflC-mCherry is therefore now named TM^HflC^-SPFH^HflC^-mCherry in Fig. 3) and, separately, only its TM region (TM^HflC^-mCherry) (Fig. 3B). We also swapped the HflC TM region with the single-spanning TM region of colistin M immunity protein (TM^Cmi^), which orients subsequent amino acids to the periplasm (33) (Fig. 3B). Epifluorescence microscopy of HflC and QmcA derivative fusions showed that in addition to native full-length constructs, only full-length constructs with a swapped TM (TM^Pf3^-QmcA-GFP and TM^Cmi^-HflC-mCherry) displayed significant punctate foci or polar localization, respectively (Fig. 3A and B), although at reduced frequency compared to native QmcA-GFP and HflC-mCherry (Fig. S5). Finally, we prepared inner and outer membrane fractions of E. coli strains expressing each QmcA and HflC derivative, and we observed that all these constructs were still mainly located in the inner membrane fraction. This indicates that, while we observed that QmcA-GFP and HflC-mCherry derivatives exhibit altered cell localization, they do not exhibit significant mislocalization and remain located in the inner membrane (Fig. S3 and S4). These results therefore indicated that specific QmcA and HflC localization requires the combination of a transmembrane and a full cytoplasmic (QmcA) or periplasmic (HflC) domain.

### Lack of cardiolipin and isoprenoid lipid synthesis alters the cell localization of QmcA and HflC.

**FIG 4:**
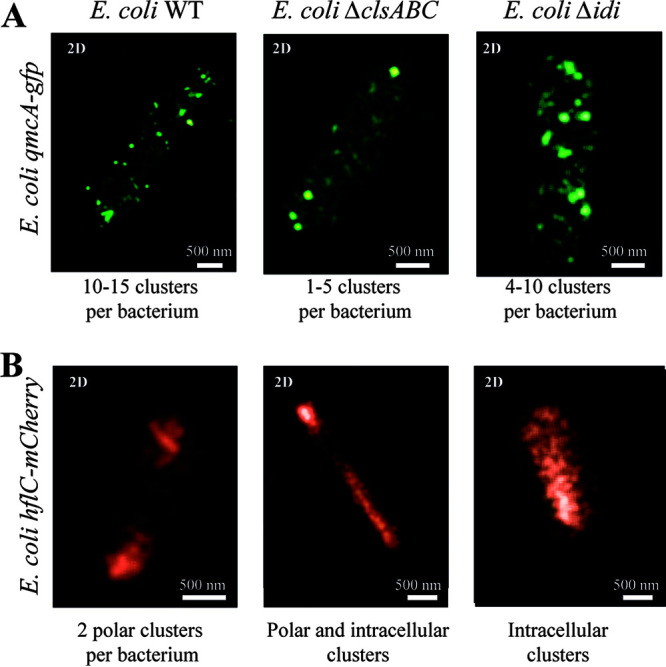
Alteration of QmcA and HflC cell localization in E. coli cardiolipin and isoprenoid pathway mutants. Two-dimensional (2D) superresolution microscopy images of WT, Δ*clsABC*, and Δ*idi* strains expressing QmcA-GFP (A) and HflC-mCherry (B) in stationary phase. The number or the nature of the detected cluster is indicated. Scales are indicated as white bars.

FMMs were proposed to be enriched with negatively charged cardiolipins and isoprenoids, which promote the localization of polar proteins and modulation of membrane lipid fluidity (15, 18, 34–39). We first tested whether alteration of cardiolipin synthesis could cause mislocalization of E. coli SPFH protein QmcA or HflKC in a mutant lacking the major cardiolipin synthases encoded by *clsABC* (40). Whereas a superresolution microscopy analysis showed only an alteration of the number of QmcA-GFP punctate foci (1 to 5 clusters per bacterium) compared to wild type (WT) (10 to 15 clusters per bacterium) (Fig. 4A and Fig. S6), the localization of HflC-mCherry showed a drastic loss of polar localization pattern (Fig. 4B and Fig. S4). We then used an *idi* mutant with reduced isoprenoid lipid synthesis due to the lack of isomerization of isopentenyl diphosphate (IPP) into dimethylallyl diphosphate (DMAPP) (41, 42). Whereas QmcA-GFP punctate localization was not affected, HflC-mCherry polar localization was abolished in the Δ*idi* mutant (Fig. 4A and Fig. S6). These results demonstrated that the alteration of the cardiolipin and, to a lesser extent, the isoprenoid lipid synthesis pathway affects HflC fusion protein localization in E. coli.

### Phenotypic analysis of an E. coli SPFH mutant shows that only the absence of HflKC increases E. coli sensitivity to aminoglycosides and oxidative stress.

**FIG 5:**
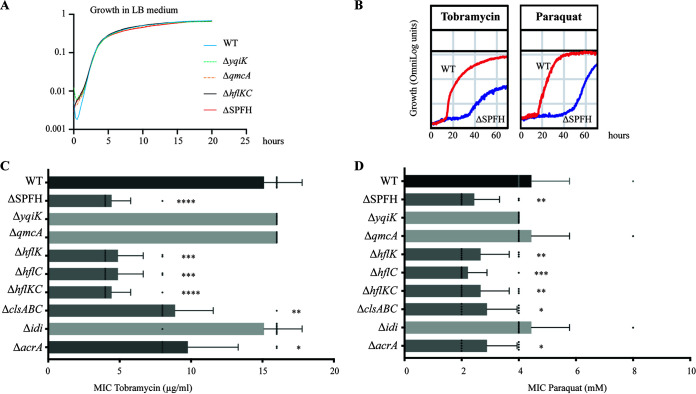
Phenotypic analysis of E. coli SPFH mutants. (A) Bacterial growth curve of WT and SPFH gene deletion mutants in LB medium. (B) Biolog bacterial growth curve of WT and Δ*SPFH* strains in the presence of tobramycin and paraquat. (C) MIC of tobramycin for E. coli WT and indicated mutants. (D) MIC of paraquat for E. coli WT and indicated mutants. *, *P* < 0.05; **, *P* < 0.01; ***, *P* < 0.001; ****, *P* < 0.0001, compared with WT.

To identify potential phenotypes and functions associated with the E. coli SPFH proteins YqiK, QmcA, HflK, and HflC, we introduced single and multiple deletions of the corresponding SPFH genes. We observed that neither single mutants nor the quadruple Δ*hflK*, Δ*hflC*, Δ*qmcA*, and Δ*yqiK* mutant (here referred to as the Δ*SPFH* mutant) displayed any significant growth defects in rich or minimal medium (Fig. 5A and Fig. S7A). Considering the role of SPFH proteins in the activation of inner membrane kinases involved in B. subtilis biofilm formation (15), we tested adhesion and biofilm capacity of WT and Δ*SPFH* strains but could not detect any significant differences between these two strains. We then used Biolog phenotypic microarrays to perform a large-scale phenotypic assay comparing E. coli WT and Δ*SPFH* mutant strains (Table S1). This analysis revealed that the Δ*SPFH* mutant is metabolically less active when grown in the presence of various aminoglycosides (tobramycin, capreomycin, sisomicin, and paromomycin) or when exposed to paraquat (Fig. 5B and Fig. S7B and C). Consistently, the MIC of tobramycin for the Δ*SPFH* mutant was 3-fold lower than that for the WT (Fig. 5C), and the sensitivity of the Δ*SPFH* mutant to paraquat was increased compared to the WT (Fig. 5D and Fig. S7D). Testing of individual SPFH gene mutants for their sensitivity to tobramycin and paraquat showed that the HflKC complex is solely responsible for both phenotypes, as both single *hflK* and *hflC* mutants or a double *hflKC* mutant displayed increased sensitivity to tobramycin and oxidative stress (Fig. 5C and D and Fig. S5E). This phenotype could be complemented upon the introduction of a plasmid expressing *hflKC* genes in the double *hflKC* mutant, and C-terminally tagged HflC-mCherry and HflK-mCherry displayed wild-type MICs for tobramycin and paraquat, indicating that both fusions were functional and relevant proxies for the bacterial localization of the HflKC complex (Fig. S8).

### Contribution of HflC localization to tobramycin and paraquat tolerance.

Whereas the determination of the MIC of tobramycin and paraquat for an Δ*idi* mutant showed no significant difference from that for the WT, the MIC of tobramycin and paraquat for a Δ*clsABC* mutant was reduced by 2- to 3-fold (Fig. 5C and D). Considering the scaffolding role of HflKC and the importance of cell localization, we tested the localization and contribution to aminoglycoside and stress tolerance of AcrA, a protein previously identified in the E. coli inner membrane potentially associated with FMM (28). AcrA is an efflux pump involved in the transport of a wide range of substrates including aminoglycosides (43). However, while an *acrA* deletion did not alter the E. coli MIC profile of tobramycin and paraquat as much as did an Δ*hflC* mutant (Fig. 5C and D), the expression of AcrA-GFP from the native chromosomal context did not lead to any distinct polar colocalization with HflC under exponential- or stationary-phase conditions (Fig. S9).

Taken together, these results indicate that the HflKC SPFH protein complex contributes to oxidative and antibiotic stress resistance.

## DISCUSSION

SPFH domain proteins have been identified in most organisms (16, 44) and extensively studied in eukaryotes (3, 5, 45). In contrast, prokaryotic SPFH proteins and proteins associated with functional membrane microdomains (FMMs) are much less understood. This is particularly the case for Gram-negative bacteria, in which potential FMM functions are mostly inferred from studies performed in B. subtilis and S. aureus. In this study, we investigated the functions and the localization determinants of E. coli SPFH proteins. Focusing on QmcA and HflKC SPFH proteins, we used a domain deletion and replacement approach and showed that most of the tested domain replacement variants correctly localized to the inner membrane but failed to display WT protein localization patterns. This indicates that inner membrane localization alone is not sufficient for the correct subcellular distribution of HflC and QmcA, whose localization signals likely rely on oligomerization for focal flotillin cluster formation (46), which could be abolished in the domain deletion constructs, potentially explaining the observed localization defects.

QmcA and HflKC SPFH proteins display different localization patterns and could be part of different FMMs, potentially using different localization signals. The punctate localization pattern displayed by the QmcA-GFP fusion was also observed in the case of E. coli YqiK expressed from plasmid and other Gram-positive bacterial flotillin homologs (15, 16, 38, 47, 48). Interestingly, B. subtilis, Bacillus anthracis and S. aureus flotillin genes are physically associated with a gene encoding an NfeD protein, which could contribute to protein-protein interactions within flotillin assemblies (49, 50). Consistently, the E. coli *qmcA* gene is located upstream of the NfeD-like *ybbJ* gene, like the NfeD-like *yqiJ* gene is located upstream of *yqiK* (see Fig. S1 in the supplemental material). This further supports the notion that QmcA and YqiK could be considered E. coli FloA/FloT homologs.

In contrast, the *hflKC* transcription unit is not closely associated with an *nfeD*-like gene, suggesting that HflKC may not be a flotillin. However, while QmcA and YqiK have an opposite orientation from HflK and HflC, they are structurally similar proteins, and the four E. coli SPFH proteins could therefore share some degrees of functionalities. The topological similarity between HflK and HflC might contribute to HflKC complex formation, and its interaction with FtsH protease, resulting in a large periplasmic FtsH-HflKC complex localized at the cell pole (24, 25, 51–54).

Along with phosphatidylethanolamine and phosphatidylglycerol, cardiolipins are the primary constituent components of E. coli membranes that concentrate into cell poles and dividing septum (55–58). It was indeed observed that the composition of E. coli membrane lipids at cell poles is altered in a *clsABC* cardiolipin-deficient mutant, compensated by an increased amount of phosphatidylglycerol (34, 59). Several studies also reported that cardiolipin-enriched composition in membranes at cell poles influences both the localization and activity of inner membrane proteins, such as respiratory chain protein complexes and the osmosensory transporter ProP (35, 36, 60–62). We showed here that, similarly to ProP, HflC and QmcA localization patterns are affected in a Δ*clsABC* mutant, suggesting that HflKC and QmcA complexes could act as scaffolds for cardiolipin-enriched FMM cargo proteins. Isoprenoid lipids such as farnesol, carotenoids, and hopanoids have been proposed to be constituents of bacterial FMMs or to interact with SPFH proteins and FMM-associated proteins (14). Consistently, blocking the S. aureus carotenoid synthetic pathway by zaragozic acid leads to flotillin mislocalization (15), and the inactivation of farnesol synthesis in a B. subtilis *yisP* mutant impairs focal localization of the FMM-associated sensor kinase KinC (15). We showed that interfering with the E. coli *idi* isoprenoid biosynthesis pathway also strongly alters the localization of HflC. This further indicates that isoprenoid lipids contribute to the formation or integrity of FMMs, possibly by altering isoprenoid-dependent membrane fluidity, as shown in S. aureus and B. subtilis FMMs (14, 63).

Our investigation of the phenotypes displayed by an E. coli mutant lacking all SPFH protein genes showed that the absence of HflKC leads to an increased susceptibility to oxidative stress and aminoglycosides. The HflKC complex was previously shown to modulate the quality control proteolytic activity of FtsH by regulating the access of misfolded membrane protein products to FtsH (24, 25, 64). E. coli Δ*hflK* and Δ*hflC* mutant strains were also shown to accumulate increased amounts of hydroxyl radical, suggesting that HflK and HflC could influence tolerance to aminoglycosides and oxidative stress by suppressing excessive hydroxyl radical production. Alternatively, HflK and HflC could contribute to tobramycin resistance via FtsH-dependent proteolytic activity (65) or favoring FMM formation and the assembly of membrane proteins and lipids, such as cardiolipin, involved in the transport and movement of aminoglycosides within cells and cell membranes. Consistently, several proteins associated with aminoglycoside transport were potentially associated with FMM composition, including transporters and several components of the AcrAB-TolC efflux pump (28), suggesting that deletion of *hflK* or *hflC* could reduce the activity of these proteins in FMMs and enhance entry of aminoglycosides. Whereas the susceptibility to aminoglycosides indeed partly relies on the AcrAB-TolC efflux pump (41, 66–68), we found that lack of AcrA only moderately decreases the MIC of tobramycin, compared to an *hflKC* mutation. We also showed that the alteration of the cardiolipin pathway in a Δ*clsABC* mutant altered both the localization and sensitivity to tobramycin and paraquat of an HflC-GFP fusion. This suggests that cardiolipin could be required for the correct localization of HflKC to FMMs at cell poles. However, we observed that, although an *idi* isoprenoid pathway mutant affects HflC localization, it does not show altered sensitivity. We cannot rule out that the effect of the Δ*clsABC* mutant on resistance to aminoglycosides and oxidative stress could be unrelated to its impact on HflKC polar localization. Alternatively, the lack of effect of the Δ*idi* mutant may be due to an uncomplete inactivation of the pathway since lycopene production in an Δ*idi* mutant is reduced by one-third but not totally abolished, due to the fact that the IPP isomerase (IDI) protein is a reversible isomerase (33).

In conclusion, the present study provides new insights into the functions of E. coli SPFH proteins and some of their interacting partners, and further experiments will be needed to fully uncover the roles played by this intriguing family of membrane proteins in Gram-negative bacteria.

## MATERIALS AND METHODS

### Bacterial strains and growth conditions.

Bacterial strains and plasmids used in this study are described in Table S2 in the supplemental material and further explained in Fig. S1 and Fig. 3. Unless stated otherwise, all experiments were performed in lysogeny broth (LB) or M63B1 minimal medium supplemented with 0.4% glucose (M63B1.G) at 37°C. Antibiotics were used at the following concentrations: kanamycin, 50 μg/mL; chloramphenicol, 25 μg/mL; ampicillin, 100 μg/mL; and zeocin, 50 μg/mL. All compounds were purchased from Sigma-Aldrich (St. Louis, MO, USA) except for zeocin (InvivoGen, Santa Cruz, CA, USA).

### Mutant construction. (i) Generation of mutants in E. coli.

Briefly, E. coli deletion or insertion mutants used in this study either originated from the E. coli Keio collection of mutants (69) or were generated by λ-red linear recombination using pKOBEG (Cm^r^) or pKOBEGA (Amp^r^) plasmids (70) using primers listed in Table S3. P1vir transduction was used to transfer mutations between different strains. When required, antibiotic resistance markers flanked by two FLP recombination target (FRT) sites were removed using the Flp recombinase (71). Plasmids used in this study were constructed using an isothermal assembly method, Gibson assembly (New England Biolabs, Ipswich, MA, USA), using primers listed in Table S3. The integrity of all cloned fragments, mutations, and plasmids was verified by PCR with specific primers and DNA sequencing.

### (ii) Construction of deletion mutants.

Δ*yqiK*, Δ*qmcA*, Δ*hflK*, Δ*hflC*, Δ*clsA*, Δ*clsB*, Δ*clsC*, Δ*idi*, and Δ*acrA* deletions were transferred into E. coli MG1655*strep* by P1vir phage transduction from the corresponding mutants in the E. coli BW25113 background of the Keio collection (69). The associated kanamycin marker was then removed using the Flp recombinase expressed from the plasmid pCP20 (71). (Details regarding the construction of all other strains used in this study are presented in Table S2).

### (iii) Construction of GFP and mCherry fusions.

For construction of GFP and mCherry fusions, see the Supplementary Methods in the supplemental material.

### (iv) Construction of complemented strains.

The *hflKC* genes were amplified from MG1655*strep* using primers listed in Table S3 and cloned the downstream of the isopropyl-β-d-thiogalactopyranoside (IPTG)-inducible promoter of a pZS*12 vector using Gibson assembly to generate pZS*12-HflKC plasmids. Then, these plasmids were introduced into Δ*hflKC* mutants, respectively, to construct complemented mutants (Table S2). A pZS*12 empty vector was also introduced into wild-type and Δ*hflKC* mutant strains. Mutants harboring these pZS*12 plasmids were incubated and used for the experiments described below in the presence of IPTG (1 mM) and ampicillin.

### Epifluorescence microscopy.

Bacteria were incubated in 5 mL of fresh LB medium and harvested at an optical density at 600 nm (OD_600_) of 0.4 for samples in exponential phase or an OD_600_ of 2.0 for samples in stationary phase. After washing twice with M63B1 medium, cells corresponding to 1 mL of the bacterial culture were pelleted by centrifugation and resuspended into 0.1 mL of M63B1 medium for exponential samples or 1 mL of the medium for stationary samples. Ten-microliter aliquots of the cell suspension were immobilized on glass slides previously covered with freshly made M63B1 medium-0.8% agarose pads. Cells were observed using a Zeiss Definite focus fluorescence microscope (Carl Zeiss, Oberkochen, Germany), equipped with an oil-immersion lens objective microscope (Pln-Apo 63×/1.4 oil Ph3). GFP or mCherry fluorescence was excited with a Zeiss Colibri LED illumination system, and the fluorescence signal was detected with Zeiss FS38 HE (Carl Zeiss) or Semrock HcRed (Semrock, Rochester, NY, USA) filters. GFP and mCherry fluorescence images were taken at 1,000- and 2,000-ms exposure, respectively. Image processing was performed using ImageJ and Adobe Photoshop. For each tested strain, the subcellular localization patterns of 50 randomly selected bacteria were evaluated four times (a total of 200 cells), and each frequency was expressed as a percentile.

### Superresolution microscopy.

Bacteria were imaged using single-molecule localization microscopy and stochastic optical reconstruction microscopy (SMLM-STORM), using a previously described method (72). Overnight cultures were fixed with 4% paraformaldehyde (PFA), permeabilized with 0.05% Triton, and labeled with either GFP monoclonal FluoTag-Q–sulfo-cyanine 5 (Cy5) or red fluorescent protein (RFP) monoclonal FluoTag-Q–Cy5, each of which is a single-domain antibody (sdAb) conjugated to Cy5. Labeling was performed at 1:250 (concentration), and washing steps were carried out three times using Abbelight’s SMART kit buffer. For imaging, Abbelight’s imaging system was used with NEO software. Abbelight’s module was added to an Olympus IX83 with a 100× total internal-reflection fluorescence (TIRF) objective, numerical aperture (NA) 1.49. We used Hamamatsu’s scientific complementary metal oxide semiconductor (sCMOS) Flash 4 camera and a 647-nm 500-mW Oxxius laser, with an astigmatic lens, to allow for three-dimensional (3D) imaging of the sample (73).

### Inner membrane separation.

E. coli overnight cultures were diluted into 1 L of fresh LB medium to an OD_600_ of 0.02 and incubated at 37°C and 180 rpm until reaching an OD_600_ of 0.4. Cells were harvested and washed once with 10 mM HEPES (pH 7.4) and stored at −20°C for at least 1 h. Bacteria were then resuspended in 10 mL of 10 mM HEPES (pH 7.4) containing 100 μL of Benzonase (3 × 10^4^ U/mL) and were passed through a French press (Thermo) at 20,000 lb/in^2^. The lysate was centrifuged at 15,000 × *g* for 15 min at 4°C to remove cell debris, and aliquots of the suspension were stored at 4°C as the whole extract. Then, the suspension was centrifuged at 100,000 × *g* for 45 min at 4°C to separate supernatant and pellets, and aliquots of the supernatant were stored at 4°C as the cytosolic and periplasmic fractions. The pellets were suspended into 600 μL of cold 10 mM HEPES (pH 7.4) and homogenized by using a 2-mL tissue grinder (Kontes Glass, Vineland, NJ, USA). Discontinuous sucrose gradients with the following composition were placed into an ultracentrifugation tube: bottom to top, 0.5 mL of 2 M sucrose, 2.0 mL of 1.5 M sucrose, and 1.0 mL of 0.8 M sucrose. Five hundred microliters of the homogenized samples was placed on the top of the sucrose gradients. The gradients were centrifuged at 100,000 × *g* for 17.5 h at 4°C. Subsequently, 400 μL aliquots was collected into 11 microtubes from top to bottom, and the samples were subjected to the immunodetection method, as described below.

### Immunodetection of inner membrane proteins.

Aliquots of samples were suspended in 4× Laemmli buffer (Bio-Rad) with 2-mercaptoethanol (Sigma) and incubated for 5 min at 98°C. The protein samples (10 μL each) were run on 4 to 20% Mini-Protean TGX stain-free precast gels (Bio-Rad) in 1× TGX buffer and then transferred to a nitrocellulose membrane using a Trans-Blot Turbo transfer system (Bio-Rad). Subsequently, the membranes were blocked using blocking buffer consisting of 5% skim milk in phosphate-buffered saline (PBS) with 0.05% Tween 20 (PBST) for 2 h at 4°C with agitation. The membranes were then incubated in PBST containing 1% skim milk with primary antibodies, polyclonal rabbit antiserum raised against ExbB and TolC (kindly given by Philippe Delepelaire), GFP (Invitrogen; A6455; Thermo Fisher Scientific, Indianapolis, IN, USA), and mCherry (Invitrogen; PA5-34974) at 1:20,000 overnight at 4°C with agitation. The membranes were washed in PBST and incubated in PBST containing 1% skim milk with a secondary antibody, anti-rabbit IgG conjugated with horseradish peroxidase (Cell Signaling; 7074S), at 1:10,000 for 2 h at 25°C with agitation. After washing the excess secondary antibody, specific bands were visualized using the enhanced chemiluminescence (ECL) prime detection method (GE Healthcare) and imaged with an imaging system, iBright CL1500 (Invitrogen).

### Microbial growth phenotypic analysis.

A high-throughput analysis for microbial growth phenotypes using a colorimetric reaction, Phenotype MicroArrays (Biolog Inc., Hayward, CA, USA), was performed in accordance with the manufacturer’s protocol. Briefly, several colonies of E. coli grown on LB agar were transferred in 10 mL of a mixture of Biolog IF-0a medium (Biolog) and sterilized water into a sterile capped test tube. The suspension was mixed gently, and the turbidity was adjusted to achieve the appropriate transmittance using the Biolog turbidimeter (Biolog). The cell suspension was diluted with the IF-0a Plus dye mix, as mentioned in the manufacturer’s protocol. One hundred microliters of the mixture suspension was inoculated into phenotypic microarray (PM) plates 1 to 3 and 9 to 20 and incubated for 72 h at 37°C. The absorbance of each well was taken every 15 min. The OmniLog software (Biolog) was used to view and edit data, to compare data lists, and to generate reports.

### Monitoring of bacterial growth.

An overnight culture of E. coli was diluted into fresh LB and M63B1 supplemented with 0.4% glucose medium to an OD_600_ of 0.05, and 200-μL aliquots were cultured in the presence or absence of paraquat (methyl viologen dichloride hydrate; Sigma-Aldrich) in 96-well microplates at 37°C for 24 h with shaking. The absorbance of each culture at 600 nm was measured every 15 min for 24 h using a microplate reader (Tecan Infinite, Mannedorf, Switzerland).

### Susceptibility of E. coli to tobramycin and paraquat.

The broth microdilution method was used to determine the MIC values of tobramycin (Sigma-Aldrich) and paraquat (Sigma-Aldrich) in 96-well microtiter plates. Briefly, 100 μL of LB medium was distributed into each well of the microtiter plates. Tobramycin was 2-fold serially diluted in each well. Five microliters of approximately 1 × 10^7^ CFU/mL of E. coli was inoculated into each well, and the plates were incubated at 37°C for 24 h. The lowest concentration that visibly inhibited bacterial growth was defined as the MIC. All strains were evaluated in biological and technical triplicates.

The spot assay was performed to evaluate the susceptibility of E. coli to paraquat. Briefly, an overnight culture of E. coli was diluted into fresh LB medium to an OD_600_ of 0.05. Ten microliters of the diluted culture was spotted on LB plates containing either no or 100 μM paraquat. The plates were incubated at 37°C for 24 h, and photographs were taken. All strains were evaluated in triplicate.

### Statistical analysis.

Data analysis was performed using GraphPad Prism 9.5 software (GraphPad, La Jolla, CA, USA). All data are expressed as mean (±standard deviation [SD]) in figures. Statistical analysis was performed using an unpaired nonparametric Mann-Whitney test. Differences were considered statistically significant for *P* values of <0.05.

### Data availability.

The data that support the findings of this study are presented in the paper and/or the supplemental material. Strains and plasmids are available from the corresponding author, J.-M.G., upon reasonable request.
