# Supplementary material for: Escherichia coli SPFH Membrane Microdomain Proteins HflKC Contribute to Aminoglycoside and Oxidative Stress Tolerance
Source: Microbiol Spectr. 2023 Jun 22;11(4):e01767-23. doi: 10.1128/spectrum.01767-23 (PMC10434171; doi:10.1128/spectrum.01767-23)
Supplement: Supplemental file 1 — Supplemental material. Download spectrum.01767-23-s0001.pdf, PDF file, 3.8 MB [file spectrum.01767-23-s0001.pdf]

SUPPLEMENTARY MATERIALS

Escherichia coli SPFH membrane microdomain proteins HflKC contributes to aminoglycoside and oxidative stress tolerance

Wessel AK, Yoshii Y *et al.*

This pdf file includes supplementary methods, supplementary figures S1 to S9 and supplementaryTables S2 and S3. Supplementary Table S1 is provided separately as a dataset.

SUPPLEMENTARY METHODS

Construction of YqiK tagged with GFP and HflK tagged with mCherry

YqiK-GFP chromosomal construct

The *yqiK* (except for the stop codon), msf*gfp,* and *aphAIII* genes encoding YqiK, msfGFP, and KmFRT cassette were amplified from MG1655 *strep*, MG1655mreB-GFP (1), and MG1655Δ*cpxP*::KmFRT (2) chromosomes, respectively. Then, the intermediate plasmid pUC18 *yqiK-gfp-aphAIII* was constructed by combining these three DNA fragments with the linearized backbone vector pUC18, using Gibson assembly (New England Biolabs, Ipswich, MA, USA) and primers described in Table S3. Next, the DNA fragment encoding YqiK-GFP-KmFRT was amplified from the plasmid pUC18*yqiK-gfp-aphAIII* using the long recombining primers described in Table S3. The resulting PCR product was introduced on its native location under the *yqiK* promoter in strain MG1655 *strep* using pKOBEG plasmid by λ-red recombination to produce YqiK-GFP.

HflK-mCherry chromosomal construct

The DNA gene block corresponding to the entire *hflK* gene except for the stop codon, mCherry, and KmFRT with 40 bp-long homology regions of upstream of the *hflK* start codon and downstream of the *aphAIII* last codon was designed from the DNA sequence of MG1655 *strep*, MG1655mreB-mCherry (3), and MG1655Δ*cpxP*::KmFRT (2) chromosomes, respectively, and purchased from Eurofins Genomics (France). Then, the DNA oligonucleotide was introduced on its native location under the *hflK* promoter in *MG1655 strep* using pKOBEG plasmid by λ-red recombination to produce HflK-mCherry.

Construction of QmcA-GFP and QmcA-derivatives tagged with GFP

The *qmcA and ybbJ*, msf*gfp,* and *aphAIII* genes encoding QmcA and YbbJ, msfGFP and KmFRT cassette, respectively, were amplified from MG1655 *strep*, MG1655mreB-GFP, and MG1655Δ*cpxP*::KmFRT (2) chromosomes. The sequence of information on the *pf3* gene was obtained from (4).

• ***TM^{QmcA}-QmcA-GFP chromosomal construct***

The intermediate plasmid pUC18*qmcA-gfp-aphAIII* was constructed by combining DNA fragments of the entire *qmcA* gene except for the stop codon, msfGFP, the entire *ybbJ* gene followed by 20 bp upstream regions of the start codon, and KmFRT, with the linearized backbone vector pUC18 using Gibson assembly (New England Biolabs). The DNA fragment encoding QmcA-GFP-KmFRT was amplified from the plasmid pUC18*qmcA-gfp-aphAIII* using the long recombining primers described in Table S3, and introduced on its native location under the *qmcA* promoter in strain MG1655 *strep* using pKOBEG plasmid by λ-red recombination to produce TM^{qmcA}-QmcA-GFP.

TM^{QmcA}-SPFH^{QmcA}-GFP chromosomal construct

The DNA fragment encoding msfGFP-KmFRT with 40 bp-long upstream and downstream homology regions was amplified from the TM^{pf3}-SPFH^{QmcA}-GFP chromosomal construct using the long recombining primers described in Table S3. Then, the resulting PCR product was introduced on the chromosome at the downstream position of the *qmcA* SPFH domain region in strain MG1655 *strep* by λ-red recombination to produce TM^{QmcA}-SPFH^{QmcA}-GFP.

• ***TM^{QmcA} -GFP chromosomal construct***

The DNA fragment encoding to msfGFP-KmFRT with 40 bp-long upstream and downstream homology regions was amplified from the TM^{QmcA}-QmcA-GFP chromosomal construct using long recombining primers described in Table S3. Then, the resulting PCR product was introduced on the chromosome at the downstream position of the *qmcA* TM domain region in strain MG1655 *strep* by λ-red recombination to produce TM^{QmcA}-GFP.

• ***ΔqmcA::zeo chromosomal construct***

The *zeo* gene with 40 bp-long upstream and downstream homology regions was amplified from

the MG1655_ λATT:*amp_GFPmut3_ΔfimAICDFGH::zeo* *ΔyadyadNecpDhtrEyadMLKC::cat* (5) using long recombining primers described in Table S3. Then, the resulting PCR product was introduced on the native location under the *qmcA* promoter in strain MG1655 strep by λ-red recombination to generate *ΔqmcA*::zeo.

• ***TM^{pf3}-QmcA-GFP chromosomal construct***

The plasmid pZS*12*TM^{pf3}-gfp-aphAIII* was constructed via pZS*12*TM^{pf3}-gfp,* by combining DNA fragments of *pf3*, msfGFP, and KmFRT, with the linearized backbone vector pZS*12 using Gibson assembly (New England Biolabs) and primers described in Table S3. Then, the intermediate plasmid pZS*12*TM^{pf3}-qmcA-gfp-aphAIII* was constructed by combining the DNA fragment of *qmcA* gene except for the native TM domain region with the linearized vector pZS*12*TM^{pf3}-gfp* using Gibson assembly (New England Biolabs). Next, the DNA fragment encoding TM^{pf3}-QmcA-GFP-KmFRT with 40 bp-long upstream and downstream homology regions was amplified from the intermediate plasmid using the long recombining primers described in Table S3. The resulting PCR product was introduced on its native location under *qmcA* promoter by λ-red recombination into strain Δ*qmcA*::*zeo* using pKOBEG plasmid to produce TM^{pf3}-QmcA-GFP.

• ***TM^{pf3}-$SPFH^{QmcA}$-GFP chromosomal construct***

The intermediate plasmid pZS*12*TM^{pf3}-$SPFH^{QmcA}$-gfp-aphAIII* was constructed by combining the SPFH domain region of *qmcA* gene with the linearized vector PZ*12 *TM^{pf3}-gfp-aphAIII* using Gibson assembly (New England Biolabs). Then, the DNA fragment encoding TM^{pf3}-$SPFH^{QmcA}$-GFP-KmFRT with 40 bp-long upstream and downstream homology regions was amplified from the plasmid pZS*12 *TM^{pf3}-$SPFH^{QmcA}$-GFP-aphAIII* using the long recombining primers described in Table S3. The resulting PCR product was introduced on its native location under *qmcA* promoter by λ-red recombination into Δ*qmcA*::*zeo* strain using pKOBEG plasmid to produce TM^{pf3}-$SPFH^{QmcA}$-GFP.

• ***TM^{pf3}-GFP chromosomal construct***

The DNA fragment encoding *TM^{pf3}*-GFP-KmFRT with 40 bp-long upstream and downstream homology regions was amplified from pZS*12*TM^{pf3}-gfp-aphAIII* using the long recombining primers described in Table S3. The resulting PCR product was introduced on its native location under *qmcA* promoter by λ-red recombination into Δ*qmcA*::*zeo* strain using pKOBEG plasmid to produce TM^{pf3}-GFP.

Construction of HflC derivatives tagged with mCherry

The *hflC*, *mCherry,* and *cat* genes encoding HflC, mCherry, and CmFRT cassette, respectively, were amplified from MG1655 *strep*, MG1655mreB-mCherry, and MG1655ΔyfcV-P::CmFRT (6) chromosomes. The sequence of information on *cmi* gene was obtained from (4).

• ***TM^{HflC}-HflC-mCherry chromosomal construct***

The intermediate plasmid pUC18*TM^{hflC}-HflC-mCherry-aphAIII* was constructed by combining DNA fragments of the *hflC* gene except for the stop codon, mCherry, and KmFRT, with the linearized backbone vector pUC18 using Gibson assembly (New England Biolabs) and primers described in Table S3. The DNA fragment encoding HflC-mCherry-KmFRT with 40 bp-long upstream and downstream homology regions was amplified from the plasmid pUC18*TM^{hflC}-HflC-mCherry-aphAIII* using the long recombining primers described in Table S3 and introduced on its native location under the *hflC* promoter in strain *MG1655 strep* using pKOBEG plasmid by λ-red recombination to TM^{hflC}-HflC-mCherry.

• ***TM^{HflC}- $SPFH^{HflC}$ -mCherry chromosomal construct***

The DNA fragment encoding mCherry-CmFRT with 40 bp-long upstream and downstream homology regions was amplified from the TM^{cmi}-$SPFH^{HflC}$-mCherry chromosomal construct, using the long recombining primers described in Table S3. The resulting PCR product was introduced on the chromosome at the downstream position of the *hflC* SPFH domain region by λ-red recombination into *MG1655 strep* strain using pKOBEGA plasmid to produce TM^{hflC}-$SPFH^{HflC}$-mCherry.

• ***TM^{HflC}-mCherry chromosomal construct***

The DNA fragment encoding mCherry-KmFRT with 40 bp-long upstream and downstream homology regions was amplified form the plasmid pUC18*TM^{hflC}-HflC-mCherry-aphAIII* using the long recombining primers described in Table S3. The resulting PCR product was introduced on the chromosome at the downstream position of the *hflC* TM domain region by λ-red recombination into *MG1655 strep* strain using pKOBEGA plasmid to produce TM^{hflC}-mCherry.

• ***TM^{cmi}-HflC-mCherry chromosomal construct***

The plasmid pZS*12*TM^{cmi}-mCherry-cat* was constructed, via pZS*12*TM^{cmi}-mCherry,* by combining DNA fragments encoding *TM^{cmi}*, mCherry, and CmFRT, with the linearized backbone vector pZS*12 using Gibson assembly (New England Biolabs) and the primers described in Table S3. Then, the intermediate plasmid pZS*12*TM^{cmi}-hflC-mCherry-cat* was constructed by combining the DNA fragment of *hflC* gene except for the native TM domain region with the linearized vector pZS*12*TM^{cmi}-mCherry-cat* using Gibson assembly (New England Biolabs). Then, the DNA fragment encoding TM^{cmi}-HflC-mCherry-CmFRT with 40 bp-long upstream and downstream homology regions was amplified from the intermediated plasmid using the long recombining primers described in Table S3. The resulting PCR product was introduced on its native location under *hflC* promoter by λ-red recombination into Δ*hflC*::KmFRT strain using pKOBEGA plasmid to produce TM^{cmi}-HflC-mCherry.

• ***TM^{cmi}- SPFH^{HflC} -mCherry chromosomal construct***

The intermediated plasmid pZS*12*TM^{cmi}-SPFH^{HflC}-mCherry-cat* was constructed by combining the *hflC* SPFH domain region with the linearized initial plasmid pZS*12*TM^{cmi}-mCherry-cat*, which was used for generating TM^{cmi}-HflC-mCherry chromosomal construct, using Gibson assembly (New England Biolabs). Then, the DNA fragment encoding TM^{cmi}-SPFH^{HflC}-mCherry-CmFRT with 40 bp-long upstream and downstream homology regions was amplified from the intermediated plasmid using the long recombining primers described in Table S3. The resulting PCR product was introduced on its native location under *hflC* promoter by λ-red recombination into Δ*hflC*::KmFRT strain using pKOBEGA plasmid to produce TM^{cmi}-SPFH^{HflC}-mCherry.

• ***TM^{cmi}- mCherry chromosomal construct***

The DNA fragment encoding TM^{cmi}-GFP-CmFRT with 40 bp-long upstream and downstream homology regions was amplified from the plasmid pZS*12*TM^{cmi}-mCherry-cat* using the long recombining primers described in Table S3. The resulting PCR product was introduced on its native location under *hflC* promoter by λ-red recombination into Δ*hflC*::KmFRT strain using pKOBEGA plasmid to produce TM^{cmi}-mCherry.

Construction of QmcA-GFP and HflC-mCherry in the ΔclsABC and Δidi mutants

• ***QmcA-GFP chromosomal construct* in the Δ*clsABC* strain**

TM^{QmcA}-QmcA-GFP-KmFRT mutation from TM^{QmcA}-QmcA-GFP chromosomal construct

was introduced into strain Δ*clsABC* by P1vir phage transduction (Δ*clsABC* QmcA-GFP).

• ***TM^{QmcA}-QmcA-GFP chromosomal construct*** **in the Δ*idi* strain**

TM^{QmcA}-QmcA-GFP-KmFRT mutation from TM^{QmcA}-QmcA-GFP chromosomal construct was introduced into strain Δ*idi* by P1vir phage transduction (Δ*idi* QmcA-GFP).

• ***TM^{HflC}-HflC-mCherry chromosomal construct*** **in the *ΔclsABC* strain**

TM^{HflC}-HflC-mCherry-KmFRT mutation from TM^{HflC}-HflC-mCherry chromosomal construct was introduced into strain Δ*clsABC* by P1vir phage transduction (Δ*clsABC* HflC-mCherry).

• ***TM^{HflC}-HflC-mCherry chromosomal construct*** **in the Δ*idi* strain**

TM^{HflC}-HflC-mCherry-KmFRT mutation from TM^{HflC}-HflC-mCherry chromosomal construct was introduced into strain Δ*idi* by P1vir phage transduction (Δ*idi* HflC-mCherry).

• ***AcrA-GFP***

The DNA fragment encoding GFP-KmFRT region with 40 bp-long upstream and downstream homology regions was amplified from the TM^{pf3}-SPFH^{QmcA}-GFP chromosomal construct using the long recombining primers described in Table S3. The resulting PCR product was introduced on chromosome at the downstream position of the *acrA* gene (without stop codons) in *MG1655 strep* by λ-red recombination, to produce a GFP fusion. The mutation harboring KmFRT was introduced into a MG1655 *strep* strain having clean background by P1vir phage transduction (AcrA-GFP).

REFERENCES

1. **Pédelacq JD, Cabantous S, Tran T, Terwilliger TC, Waldo GS.** 2006. Engineering and characterization of a superfolder green fluorescent protein. Nat Biotechnol **24:**79-88.10.1038/nbt1172: 10.1038/nbt1172

2. **Beloin C, Valle J, Latour-Lambert P, Faure P, Kzreminski M, Balestrino D, Haagensen JA, Molin S, Prensier G, Arbeille B, Ghigo JM.** 2004. Global impact of mature biofilm lifestyle on *Escherichia coli* K-12 gene expression. Mol Microbiol **51:**659-674.10.1046/j.1365-2958.2003.03865.x: 10.1046/j.1365-2958.2003.03865.x

3. **Bendezú FO, Hale CA, Bernhardt TG, de Boer PA.** 2009. RodZ (YfgA) is required for proper assembly of the MreB actin cytoskeleton and cell shape in *E. coli*. Embo j **28:**193-204.10.1038/emboj.2008.264: 10.1038/emboj.2008.264

4. **Luiten RG, Putterman DG, Schoenmakers JG, Konings RN, Day LA.** 1985. Nucleotide sequence of the genome of Pf3, an IncP-1 plasmid-specific filamentous bacteriophage of Pseudomonas aeruginosa. J Virol **56:**268-276

5. **Larsonneur F, Martin FA, Mallet A, Martinez-Gil M, Semetey V, Ghigo JM, Beloin C.** 2016. Functional analysis of Escherichia coli Yad fimbriae reveals their potential role in environmental persistence. Environmental microbiology 18:5228-5248.

6. **Korea CG, Badouraly R, Prevost MC, Ghigo JM, Beloin C.** 2010. *Escherichia coli* K-12 possesses multiple cryptic but functional chaperone-usher fimbriae with distinct surface specificities. Environ Microbiol **12**: 1957–1977. https://doi.org/10.1111/j.1462-2920.2010.02202.x

SUPPLENTARY FIGURES

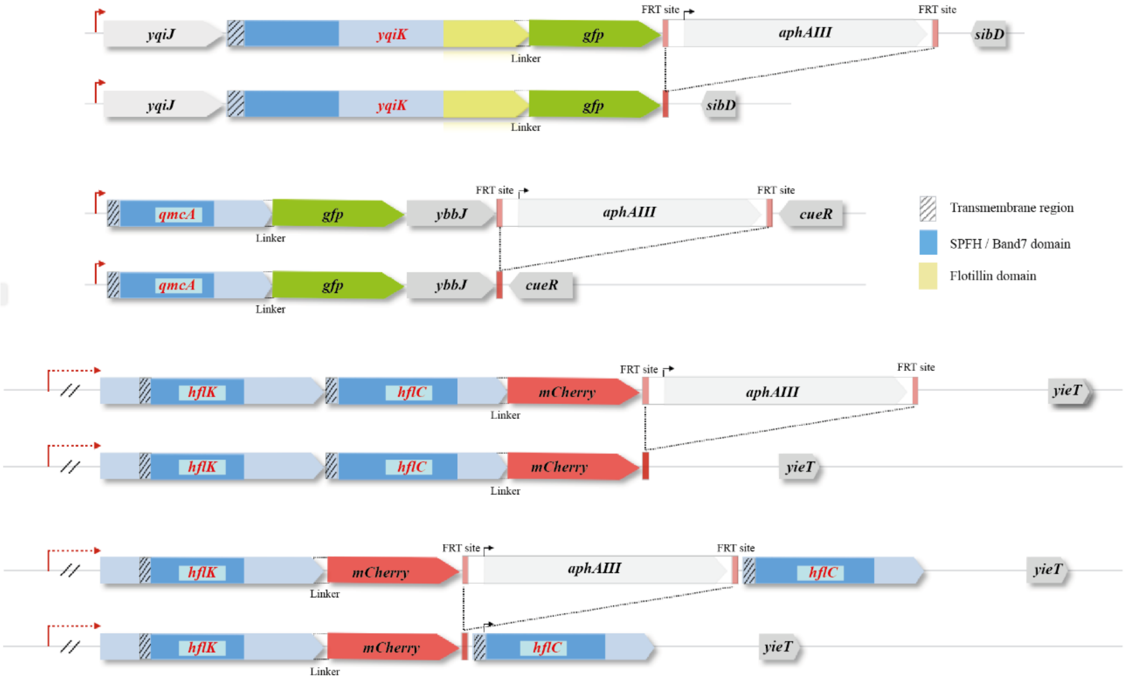

Supplementary Figure S1. Schematic representation of chromosomal GFP or mCherry fusions to genes encoding *E. coli* SPFH proteins. SPFH protein-encoding genes, *yqiK*, *qmcA*, are fused with monomeric super folder green fluorescent protein GFP (msfGFP) and *hflK*, and *hflC* to monomeric red fluorescent protein mCherry, respectively. Fusions were constructed as described in the Materials and Method section and the associated kanamycin antibiotic resistance marker (*aphAIII)* was inserted downstream of the fusion genes and removed using pCP20 plasmid, encoding the *flp* flippase.

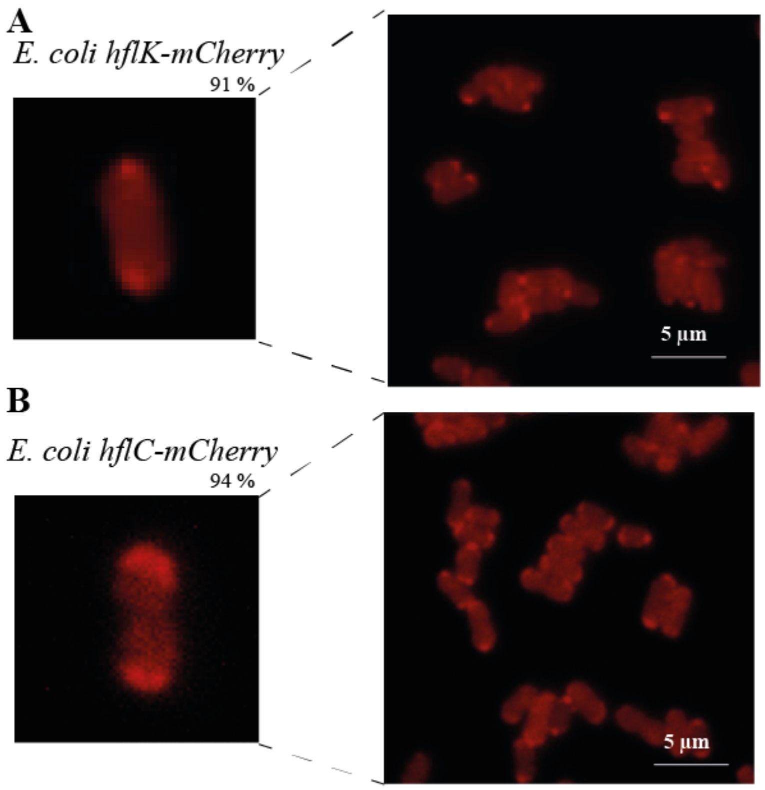

Supplementary Figure S2. Localization pattern of HflK and HflC. Epifluorescence microscopic images of cells expressing HflK-mCherry (A) and HflC-mCherry (B) in exponential phase with high and low magnifications.

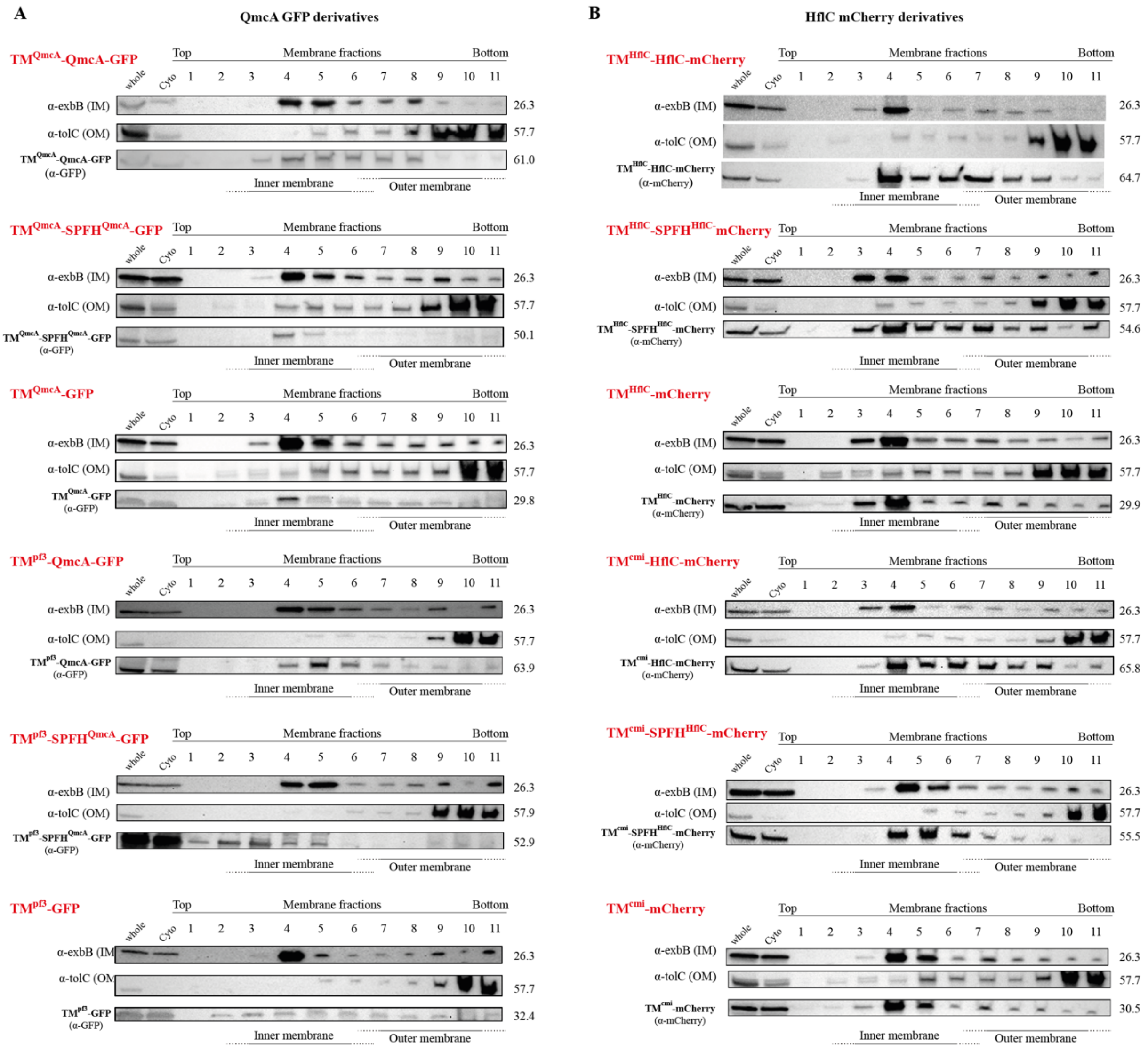

Supplementary Figure S3. Inner membrane localization of QmcA and HflC in domain swap constructs. SDS-PAGE and immunodetection analyses using proteins of whole-cell extract, cytosolic fraction, and membrane fractions prepared from cells expressing GFP fusion derivatives of QmcA (**A**) or mCherry fusion derivatives of HflC. (**B**). In immunodetection analysis, anti-ExbB antibodies were used to detect the inner membrane- (IM) marker ExbB and anti-TolC antibodies to detect the outer membrane- (OM) marker TolC. Anti-GFP and anti-mCherry antibodies were used to detect the expressions of GFP and mCherry fused proteins, respectively.

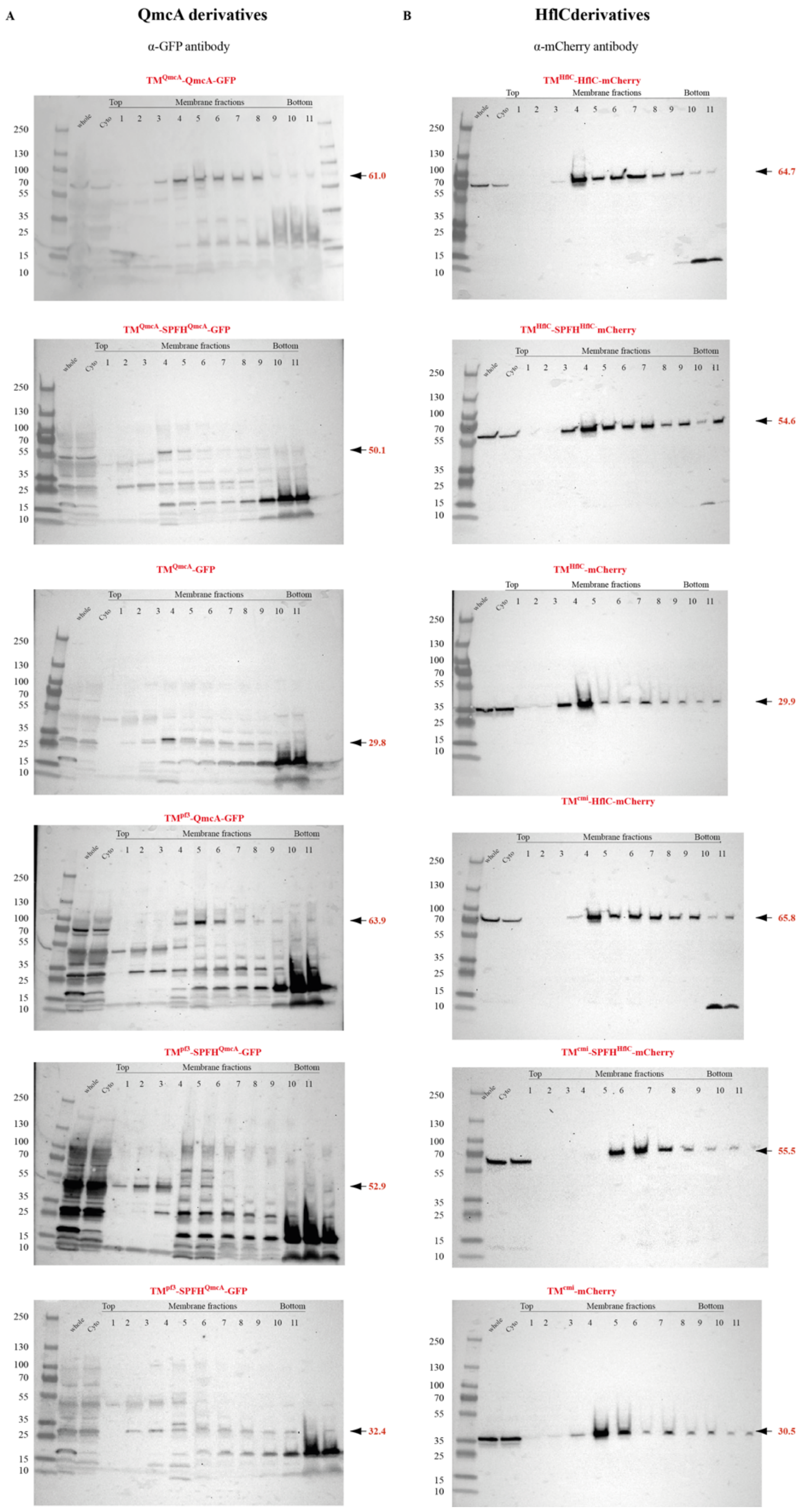

Supplementary Figure S4. Detection of QmcA and HflC fusion derivatives in domain swap constructs. A: immunodetection of GFP fusion derivatives of QmcA using anti-GFP antibody. **B:** immunodetection of mCherry fusion derivatives of HflC using anti-mCherry antibody.

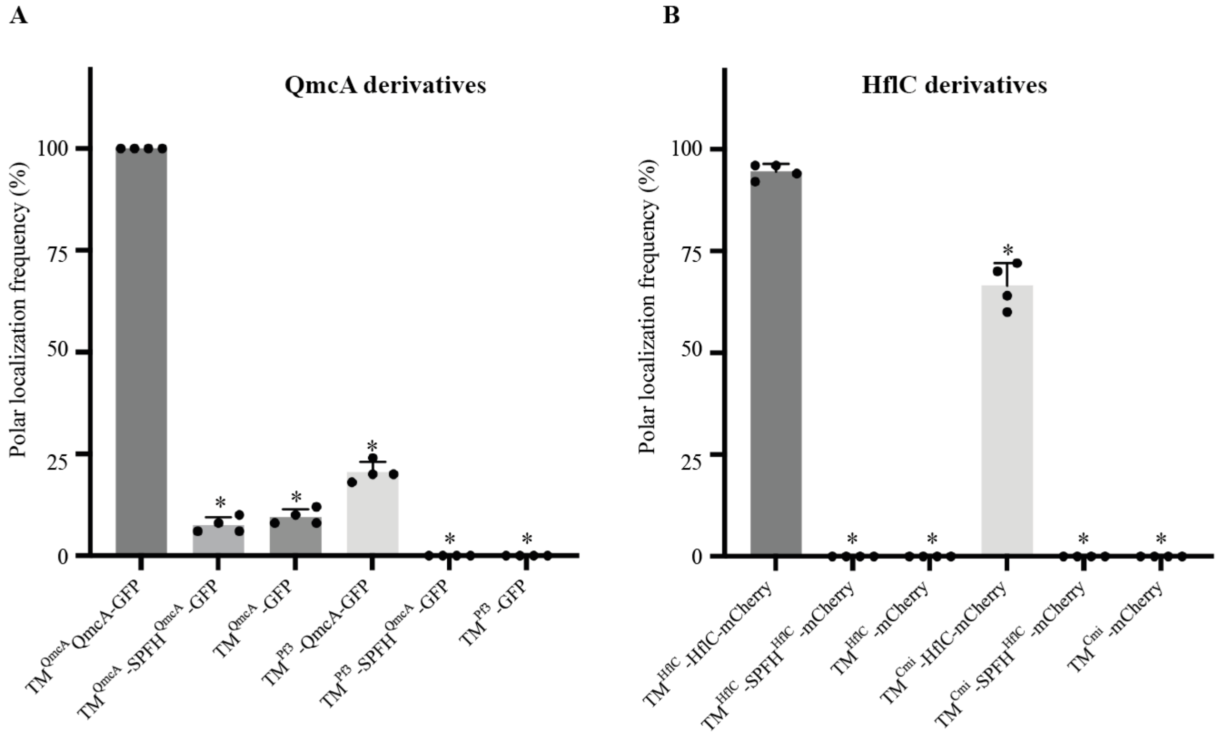

Supplementary Figure S5. Quantification data for punctate or polar localizations in QmcA and HflC derivatives,. **A:** QmcA derivatives. **B:** HflC derivatives. *p < 0.05 compared with TM^{QmcA}-QmcA-GFP or TM^{HflC}-HflC-mCherry.

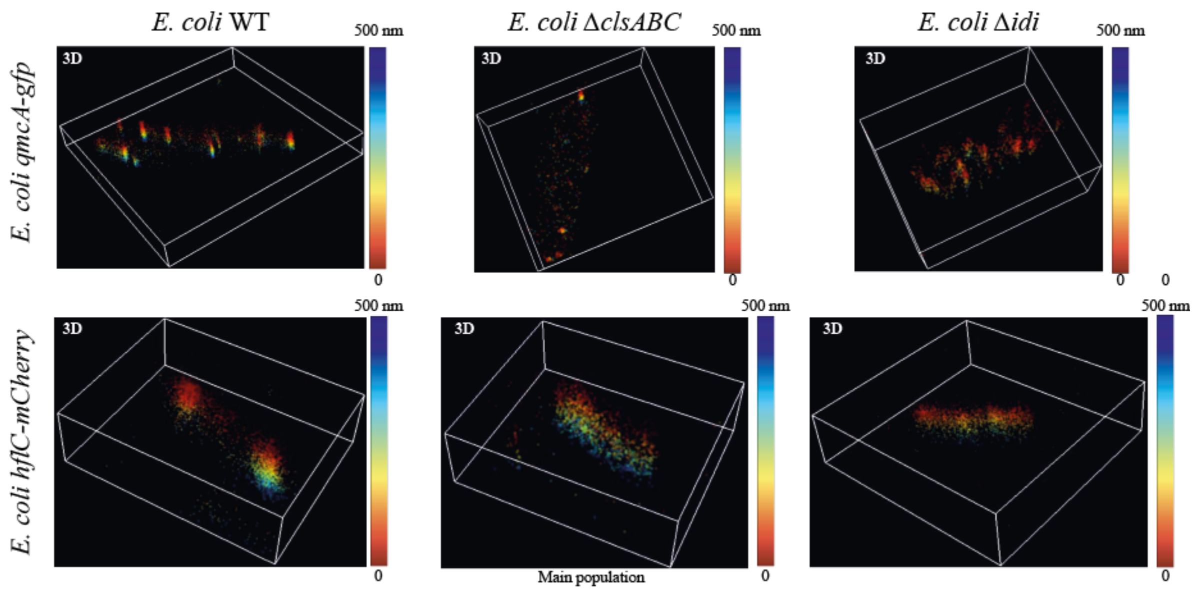

Supplementary Figure S6. The subcellular localization of QmcA and HflC in mutants with the alteration of cardiolipin or isoprenoid synthetic pathway. Three-dimensional super-resolution microscopy images of WT, Δ*clsABC*, and Δ*idi* strains expressing QmcA-GFP and HflC-mCherry in stationary phase, with colors corresponding to depth location along the Z axis, 0-500 nm, with 0 nm expressed in red, and 500 nm expressed in deep blue.

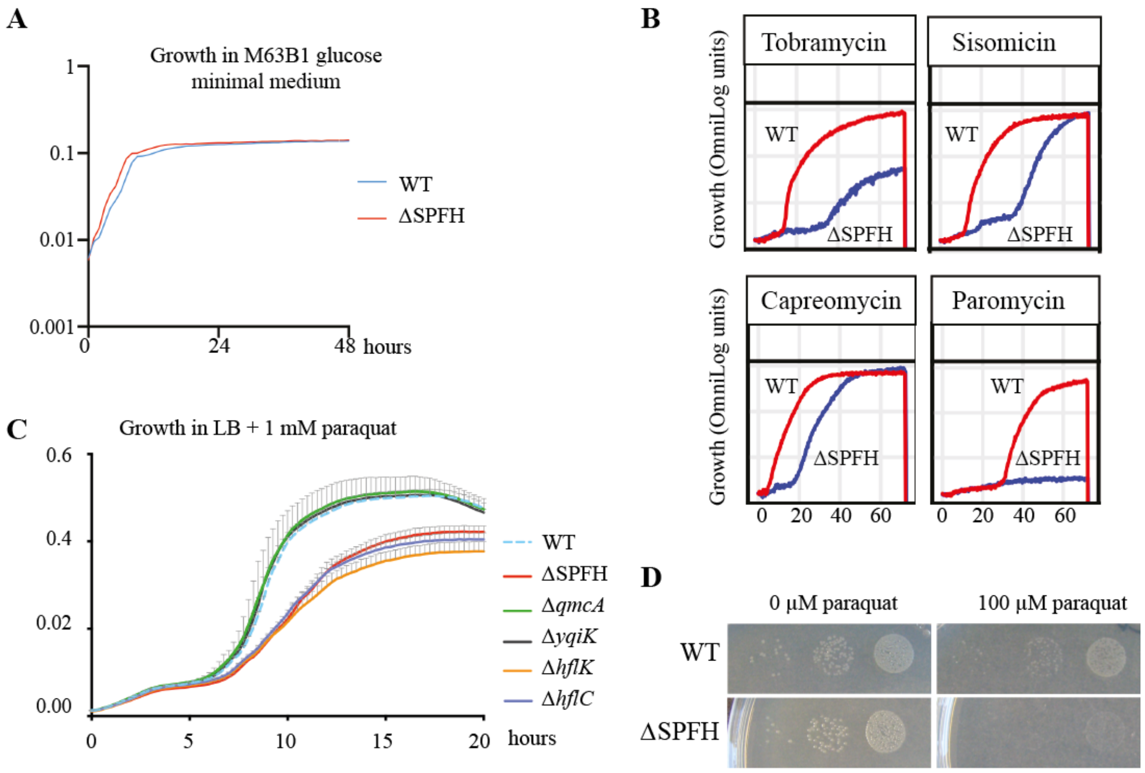

Supplementary Figure S7. Phenotypic analysis of *E. coli* SPFH mutant

(**A**) Growth curve of *E. coli* WT and ΔSPFH mutant in M63B1glucose minimal medium. (**B**) Biolog metabolic activity curve of WT and ΔSPFH in presence of tobramycin, capreomycin, sisomicin, and paromycin. (**C**) Bacterial growth curve of WT and ΔSPFH in the presence of 0.8 mM paraquat. (**D**) Paraquat susceptibility assay of WT and ΔSPFH spotted on M63B1glucose agar containing 100 µM paraquat. (**E**) Growth curve of WT and indicated SPFH gene mutants in the presence of 0.8 mM paraquat.

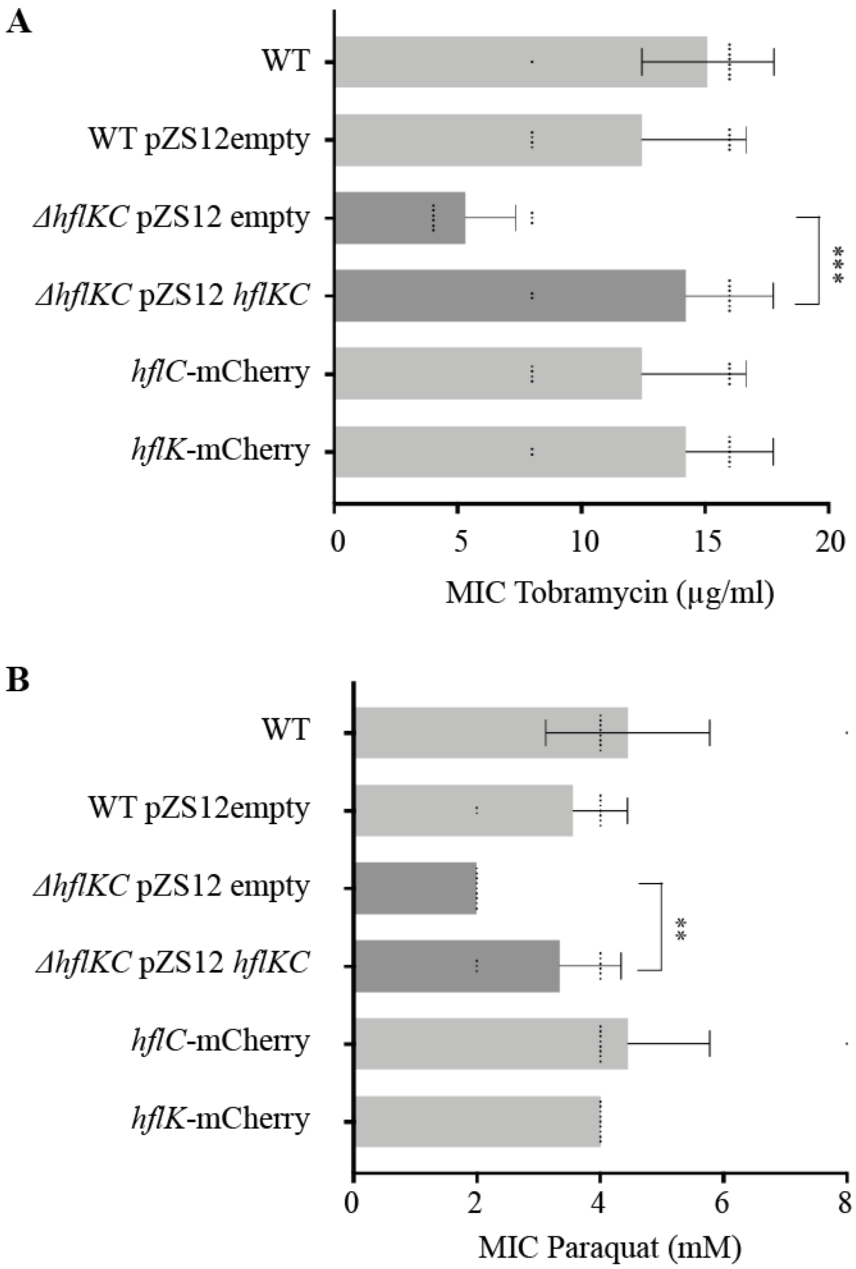

Supplementary Figure S8. Susceptibility to tobramycin and paraquat of complemented mutants. Minimum inhibitory concentrations (MICs) for tobramycin (**A**) and paraquat (**B**) of *E. coli* Wt, Δ*hflKC* strains harboring empty vector and *hflKC*- complemented strains or strain expressing *hflC or hflK-mCherry constructs* from their native chromosomal context. $**p < 0.01$, $***p < 0.001$.

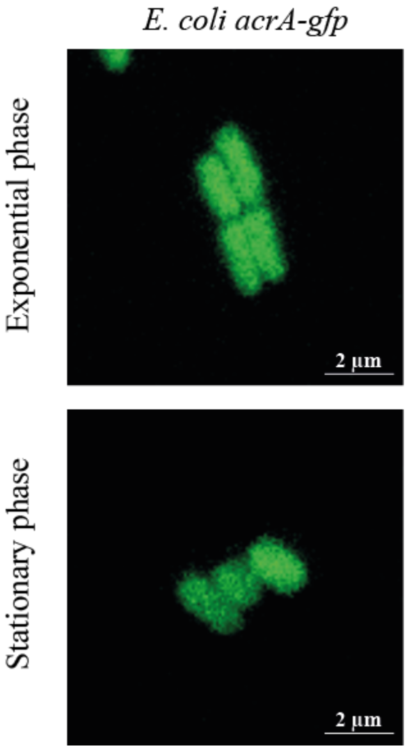

Supplementary Figure S9. Localization of AcrA-GFP fusion protein does not show polar localization pattern when expressed from native chromosomal context. Epifluorescence microscopic images of cells expressing AcrA-GFP in exponential and stationary phases.

Supplementary Table S2. Bacterial strains and plasmids used in this study

E. coli strains and plasmids	Genotype or description	Antibiotic resistance(s)	Reference
**Strains**			
BW25113Δ*yqiK*::Kmfrt	Keio collection	Km^R	(1)
BW25113Δ*qmcA*::Kmfrt	Keio collection	Km^R	(1)
BW25113Δ*hflK*::Kmfrt	Keio collection	Km^R	(1)
BW25113Δ*hflC*::Kmfrt	Keio collection	Km^R	(1)
BW25113Δ*clsA*::Kmfrt	Keio collection	Km^R	(1)
BW25113Δ*clsB*::Kmfrt	Keio collection	Km^R	(1)
BW25113Δ*clsC*::Kmfrt	Keio collection	Km^R	(1)
BW25113Δ*idi*::Kmfrt	Keio collection	Km^R	(1)
BW25113Δ*acrA*::Kmfrt	Keio collection	Km^R	(1)
MG1655 *strep*	Spontaneous streptomycin-resistant mutant of K-12 wild-type strain	$Strep^R$	(2)
MG1655mreB-GFP	Source of msf*gfp* gene	No resistance	Gift from Dr. Sven van Teeffelen
MG1655mreB-mCherry	Source of *mCherry* gene	No resistance	Gift from Dr. Sven van Teeffelen
MG1655Δ*cpxP*::KmFRT	Source of kanamycine resistance cassette	Km^R	(3)
MG1655ΔyfcV-P::CmFRT	Source of chloramphenicol resistance cassette	Cm^R	(4)
DH5alpir pSW25 ptetAccdB	Source of spectinomycin resistance cassette	$Spec^R$	(5)

MG1655_λATT:*amp_GFPmut3_ΔfimAICDFGH*::zeo Δ*yadyadNecpDhtrEyadMLKC*::*cat*	Source of zeocin resistance cassette	Zeo^R	(6)
Δ*yqiK*	P1 vir transduction from BW25113Δ*yqiK*::Kmfrt into MG1655 *strep*	Strep^R	This study
Δ*qmcA*	P1 vir transduction from BW25113Δ*qmcA*::Kmfrt into MG1655 *strep*	Strep^R	This study
Δ*hflK*	P1 vir transduction from BW25113Δ*hflK*::Kmfrt into MG1655 *strep*	Strep^R	This study
Δ*hflC*	P1 vir transduction from BW25113Δ*hflC*::Kmfrt into MG1655 *strep*	Strep^R	This study
Δ*hflKC*	Double mutant of Δ*hflK* and Δ*hflC*. MG1655*strepR*, Δ*hlfKC*::Cmfrt	Strep^R	This study
Δ*yqiJK*	Double mutant of Δ*yqiJ* and Δ*yqiK*. MG1655*strepR*, Δ*yqiJK*::Spec	Strep^R, Spec^R	This study
Δ*qmcA*, Δ*yqiJK*	Triple mutant of Δ*qmcA* and Δ*yqiJK*. MG1655*strepR*, Δ*qmcA,* Δ*yqiJK*::Spec	Strep^R, Spec^R	This study
Δ*SPFH*	Quintuple mutant of Δ*yqiK*, Δ*qmcA*, Δ*hflK*, and Δ*hflC*. MG1655*strepR*, Δ*qmcA*, Δ*yqiJK*::Spec, Δ*hlfKC*::Cmfrt	Strep^R, Spec^R	This study
Δ*clsA*	P1 vir transduction from BW25113Δ*clsA*::Kmfrt into MG1655 *strep*	Strep^R	This study
Δ*clsB*	P1 vir transduction from BW25113Δ*clsB*::Kmfrt into MG1655 *strep*	Strep^R	This study
Δ*clsC*	P1 vir transduction from BW25113Δ*clsC*::Kmfrt into MG1655 *strep*	Strep^R	This study

Δ*clsAB*	Double mutant of Δ*clsA* and Δ*clsB.* P1 vir transduction from BW25113Δ*clsA*::Kmfrt into Δ*clsB*	Strep^R	This study
Δ*clsABC*	Triple mutant of Δ*clsA*, Δ*clsB*, and Δ*clsC*. P1 vir transduction from BW25113Δ*clsC*::Kmfrt into Δ*clsAB*	Strep^R	This study
Δ*idi*	P1 vir transduction from BW25113Δ*idi*::Kmfrt into MG1655 *strep*	Strep^R	This study
Δ*acrA*	P1 vir transduction from BW25113Δ*acrA*::Kmfrt into MG1655 *strep*	Strep^R	This study
Δ*qmcA*::*zeo*	MG1655 *strep,* Δ*qmcA*::*zeo*	Strep^R, Zeo^R	This study
yqiK-gfp	MG1655 *strep, yqiK*::*gfp* Kmfrt	Strep^R, Km^R	This study
qmcA-gfp	MG1655 *strep, qmcA*::*gfp* Kmfrt	Strep^R, Km^R	This study
hflK-mCherry	MG1655 *strep, hflK*::*mCherry* Kmfrt	Strep^R, Km^R	This study
hflC-mCherry	MG1655 *strep, hflC*::*mCherry* Kmfrt	Strep^R, Km^R	This study
TM^{qmcA}-$SPFH^{qmcA}$-*gfp*	MG1655 *strep,* Δ*qmcA*::TM^{qmcA}-$SPFH^{qmcA}$-*gfp* Kmfrt	Strep^R, Km^R	This study
TM^{qmcA}-*gfp*	MG1655 *strep,* Δ*qmcA*::TM^{qmcA}-*gfp* Kmfrt	Strep^R, Km^R	This study
TM^{Pf3}-*qmcA-gfp*	MG1655 *strep,* Δ*qmcA*::TM^{pf3}-*qmcA-gfp* Kmfrt	Strep^R, Km^R	This study
TM^{Pf3}-$SPFH^{qmcA}$-*gfp*	MG1655 *strep,* Δ*qmcA*::TM^{pf3}-$SPFH^{qmcA}$-*gfp* Kmfrt	Strep^R, Km^R	This study
TM^{Pf3}-*gfp*	MG1655 *strep,* Δ*qmcA*::TM^{pf3}-*gfp* Kmfrt	Strep^R, Km^R	This study
TM^{hflC}-$SPFH^{hflC}$-*mCherry*	MG1655 *strep,* ΔhflC::TM^{hflC}-$SPFH^{hflC}$-*mCherry* Cmfrt	Strep^R, Cm^R	This study
TM^{hflC}-*mCherry*	MG1655 *strep,* ΔhflC::TM^{hflC}-*mCherry* Kmfrt	Strep^R, Km^R	This study
TM^{cmi}-*hflC-mCherry*	MG1655 *strep,* Δ*hflC*::TM^{cmi}-*hflC- mCherry* Cmfrt	Strep^R, Cm^R	This study
TM^{cmi}-$SPFH^{hflC}$-*mCherry*	MG1655 *strep,* Δ*hflC*::TM^{Cmi} -$SPFH^{hflC}$-*mCherry* Cmfrt	Strep^R, Cm^R	This study
TM^{cmi}-*mCherry*	MG1655 *strep,* Δ*hflC*::TM^{Cmi}-*mCherry* Cmfrt	Strep^R, Cm^R	This study

Δ*clsABC qmcA-gfp*	P1 vir transduction from *qmcA-gfp* into Δ*clsABC*	$Strep^R$, Km^R	This study
Δ*idi qmcA-gfp*	P1 vir transduction from *qmcA-gfp* into Δ*idi*	$Strep^R$, Km^R	This study
Δ*clsABC hflC-mCherry*	P1 vir transduction from *hflC-mCherry* into Δ*clsABC*	$Strep^R$, Km^R	This study
Δ*idi hflC-mCherry*	P1 vir transduction from *hflC-mCherry* into Δ*idi*	$Strep^R$, Km^R	This study
acrA-gfp	MG1655 *strep, acrA*::*gfp* Kmfrt	$Strep^R$, Km^R	This study
**Plasmids**			
pKOBEG	*ori*R101*ts araC* arabinose-inducible λred γβα operon	Cm^R	(7)
pKOBEGA	pKOBEG derivative	Amp^R	(7)
pCP20	Rep(Ts) Flp^+	Cm^R, Amp^R	(8)
pUC18	High-copy-number, lac promoter cloning vector, ampicillin resistant (AmpR)	Amp^R	(9)
pZS*12	Tightly regulated $P_{LtetO-1}$ Low copy-number vector ampicillin resistant (AmpR)	Amp^R	(10)
pUC18y*qiK-gfp-aphAIII*	Assembly of *YqiK-mCherry-aphAIII* into pUC18	Amp^R, Km^R	This study
pUC18TM^{QmcA}-*QmcA-gfp-aphAIII*	Assembly of TM^{QmcA}-*QmcA-gfp-aphAIII* into pUC18	Amp^R, Km^R	This study
pZS*12TM^{pf3}-*gfp*	Assembly of TM^{pf3}-*gfp* into pZS*12	Amp^R	This study
pZS*12TM^{pf3}-*gfp-aphAIII*	Assembly of *aphAIII* into PZ*12TM^{pf3}-*gfp*	Amp^R, Km^R	This study
pZS*12TM^{pf3}-*QmcA-gfp-aphAIII*	Assembly of *qmcA* into PZ*12TM^{pf3}-*gfp-aphAIII*	Amp^R, Km^R	This study
pZS*12 TM^{pf3}-$SPFH^{QmcA}$-*gfp-aphAIII*	Assembly of $SPFH^{qmcA}$ into PZ*12TM^{pf3}-*gfp-aphAIII*	Amp^R, Km^R	This study
pUC18TM^{HflC}-*HflC-mCherry-aphAIII*	Assembly of TM^{HflC}-*HflC-mCherry-aphAIII* into pUC18	Amp^R, Km^R	This study
pZS*12TM^{cmi}-*mCherry*	Assembly of *cmi-mCherry* into pZS*12	Amp^R	This study
pZS*12TM^{cmi}-*mCherry-cat*	Assembly of *Cmfrt* into PZ*12 *cmi-mCherry*	Amp^R, Cm^R	This study

pZS*12*TM^cmi^-hflC-mCherry-cat*	Assembly of *hflC* into PZ*12 *cmi-mCherry-Cmfrt*	Amp^R, Cm^R	This study
pZS*12*TM^cmi^-SPFH^HflC^-mCherry-cat*	Assembly of *SPFH^HflC^* into PZ*12 *cmi-mCherry-Cmfrt*	Amp^R, Cm^R	This study
pZS*12-*HflKC*	Assembly of *hflKC* into pZS*12	Amp^R	This study

REFERENCES

1. **Baba T, Ara T, Hasegawa M, Takai Y, Okumura Y, Baba M, Datsenko KA, Tomita M, Wanner BL, Mori H.** 2006. Construction of Escherichia coli K-12 in-frame, single-gene knockout mutants: the Keio collection. Molecular systems biology **2:**2006.0008. https://doi.org/10.1038/msb4100050

2. **Ferrières L, Hémery G, Nham T, Guérout AM, Mazel D, Beloin C, Ghigo JM.** 2010. Silent mischief: bacteriophage Mu insertions contaminate products of Escherichia coli random mutagenesis performed using suicidal transposon delivery plasmids mobilized by broad-host-range RP4 conjugative machinery. Journal of bacteriology **192:** 6418–6427. https://doi.org/10.1128/JB.00621-10

3. **Beloin C, Valle J, Latour-Lambert P, Faure P, Kzreminski M, Balestrino D, Haagensen JA, Molin S, Prensier G, Arbeille B, Ghigo JM.** 2004. Global impact of mature biofilm lifestyle on Escherichia coli K-12 gene expression. Molecular microbiology **51:**659–674. https://doi.org/10.1046/j.1365-2958.2003.03865.x

4. **Korea CG, Badouraly R, Prevost MC, Ghigo JM, Beloin C.** 2010. *Escherichia coli* K-12 possesses multiple cryptic but functional chaperone-usher fimbriae with distinct surface specificities. Environmental microbiology **12:**1957–1977. https://doi.org/10.1111/j.1462-2920.2010.02202.x

5. **Létoffé S, Audrain B, Bernier SP, Delepierre M, Ghigo JM**. 2014. Aerial exposure to the bacterial volatile compound trimethylamine modifies antibiotic resistance of physically separated bacteria by raising culture medium pH. mBio 5:e00944-00913.10.1128/mBio.00944-13: 10.1128/mBio.00944-13

6. **Larsonneur F, Martin FA, Mallet A, Martinez-Gil M, Semetey V, Ghigo JM, Beloin C.** 2016. Functional analysis of *Escherichia coli* Yad fimbriae reveals their potential role in environmental persistence. Environmental microbiology **18:**5228-5248. doi: 10.1111/1462-2920.13559. Epub 2016 Oct 24. PMID: 27696649.

7. **Chaveroche MK, Ghigo JM, d'Enfert C.** 2000. A rapid method for efficient gene replacement in the filamentous fungus Aspergillus nidulans. Nucleic acids research **28:** E97. https://doi.org/10.1093/nar/28.22.e97

8. **Cherepanov PP, Wackernagel W**. 1995. Gene disruption in Escherichia coli: TcR and KmR cassettes with the option of Flp-catalyzed excision of the antibiotic-resistance determinant. Gene **158:**9–14. https://doi.org/10.1016/0378-1119(95)00193-a

9. **Norrander J, Kempe T, Messing J.** 1983. Construction of improved M13 vectors using oligodeoxynucleotide-directed mutagenesis. Gene. 26:101-6. doi: 10.1016/0378-1119(83)90040-9.

10. **Lutz R, Bujard H.** 1997. Independent and tight regulation of transcriptional units in Escherichia coli via the LacR/O, the TetR/O and AraC/I1-I2 regulatory elements. Nucleic Acids Res **25:**1203-1210.10.1093/nar/25.6.1203: 10.1093/nar/25.6.1203

Supplementary Table S3. Primers used for generating chromosomal mutants or assembling plasmids in this study

Purpose	Name	Sequence (5'-3')
For amplifying the DNA fragment to generate Δ*hflKC* strain	Up.ΔhflKC-CmFRT-5	gacaacagggatcaccgcataacaaatatggagcacaaacgtgtaggctggagctgcttcgaag
	Down.ΔhflKC-CmFRT-3	ggatgcggtggctttattgacctgtaccgcagtcgttatacatatgaatatcctccttagttcc
For amplifying the DNA fragment to generate Δ*yqiJK* strain	Up.ΔyqiJK-Spec-5	caaagtaaatattgcgtcactaaatggacattggagtgatatgcgtcaattgcgccgaataaatac
	Down.ΔyqiJK-Spec-3	ccccggctttttgggccagggattattcctggaagagggttcattggctggcaccaagcagttt
For amplifying the DNA fragment to generate Δ*qmcA*::*zeo* strain	Up.ΔqmcA-Zeo-5	caattttgctgattatggacggtacaacaggaggtttttccgttctggatccgagcacgtgttgac
	Down.ΔqmcA-Zeo-3	gactgagccagaaaatatgtggatgaacgaccattaactccatctcagtcctgctcctcggccacg
For assembling the pUC18y*qiK-gfp-aphAIII* plasmid	Up.GFP_pUC18yqiK-gfp-5	caccccttaacctcaacaactcccgtcgaagaaaaagcagagggcggcggcggcagcatgagtaaaggtgaagaac
	Down.GFP_pUC18yqiK-gfp-3	caaaatctttctgtcgttatttgtagagttcatccatg
	Up.KmFRT_pUC18yqiK-gfp-5	cgacagaaagattttgggagcaaatgatgtgtgtaggc
	Down.KmFRT_pUC18yqiK-gfp-3	ccccggctttttgggccagggattattcctggaagagggtcagggtttactcatatgaatatc
	Up.linearized pUC18_pUC18yqiK-gfp-5	gcccaaaaagccgggggaattcgtaatcatggtcatag
	Down.linearized pUC18_pUC18yqiK-gfp-5	gttgaggttaagggtgggatcctctagagtcgacctg
For amplifying the DNA fragment to generate YqiK-GFP strain	Up.yqiK_YqiK-GFP-KmFRT-5	Same as Up.GFP_pUC18yqiK-gfp-5
	Down.KmFRT_YqiK-GFP-KmFRT-3	Same as Down.KmFRT_pUC18yqiK-gfp-3
For assembling the pUC18TM^{QmcA}-*QmcA-gfp-aphAIII* plasmid	Up.qmcA-gfp_pUC18qmcA-gfp-5	ccgagctggtgaaagacagcgccaacaaacggactcagccaggcggcggcggcagcatgagtaaaggtgaagaactgttc
	Down.qmcA-gfp_pUC18qmcA-gfp-3	ggctgagtccgtttgttggcttatttgtagagttcatccatgccgtgcgtgatacctgctg
	Up.ybbJ_pUC18qmcA-gfp-5	cagcaggtatcacgcacggcatggatgaactctacaaataagccaacaaacggactcagcc
	Down.ybbJ_pUC18qmcA-gfp-3	caaaatctttctgtcgttaagacgagacggctcggatatg
	Up.KmFRT_pUC18qmcA-gfp-5	cgacagaaagattttgggagcaaatgatgtgtgtaggc

	Down.KmFRT_pUC18qmcA-gfp-3	gaaaatctctccggctgctgtcatcatcgggcagggtgatcagggtttactcatatgaatatc
	Up.linearized pUC18_pUC18qmcA-gfp-5	cagccggagagattttcgaattcgtaatcatggtcatag
	Down.linearized pUC18_pUC18qmcA-gfp-5	ctttcaccagctcggggatcctctagagtcgacctg
For amplifying the DNA fragment to generate TM^{qmcA}-QmcA-GFP strain	Up.qmcA_QmcA-GFP-KmFRT-5	Same as Up.qmcA-gfp_pUC18qmcA-gfp-5
	Down.KmFRT_QmcA-GFP-KmFRT-3	Down.KmFRT_pUC18qmcA-gfp-3
For amplifying the DNA fragment to generate TM^{qmcA}-SPFH^{qmcA}-GFP strain	UP.SPFHqmcA_SPFHqmcA-GFP-KmFRT-5	gcggaagggatccgtcaggcggaaatcctcaaagccgaaggtgaatttaacaacaacatgagtaaagg
	Down.KmFRT_SPFHqmcA-GFP-KmFRT-3	gaaaatctctccggctgctgtcatcatcgggcagggtgatcagggtttactcatatgaatatc
For amplifying the DNA fragment to generate TM^{qmcA}-GFP strain	Up.TMqmcA_TMqmcA-GFP-KmFRT-5	ctcatttttgtcgcgctggtcattgtcggcgcgggtgtcaaaatcgtagaatttaacaacaacatgagtaaagg
	Down.KmFRT_TMqmcA-GFP-KmFRT-3	Same sequence as Down.KmFRT_SPFHqmcA-GFP-KmFRT-3
For assembling the pZS*12*TM*^{pf3}-*gfp* plasmid	Up.pf3-GFP_pZS*12pf3-gfp-5	gtgagcggataacaagatacatgcaatccgtgattactg
	Down.pf3-GFP_pZS*12pf3-gfp-3	ctttactcatgttgttgttaaattcaaagaattg
	Up.GFP _pZS*12pf3-gfp-5	taacaacaacatgagtaaaggtgaagaac
	Down.GFP_pZS*12pf3-gfp-3	tctagactcagctaattaagtcatttgtagagttcatcc
	Up.linearized pZS*12 pZS*12pf3-gfp-5	ctacaaatgacttaattagctgagtctagag
	Down.linearized pZS*12pZS*12pf3-gfp-3	cggattgcatgtatcttgttatccgctc
For assembling the pZS*12*TM*^{pf3}-*gfp-aphAIII* plasmid	Up.KmFRT _pZS*12pf3-gfp-KmFRT-5	cgaaaggctcagtcgaacgacagaaagattttgggag
	Down.KmFRT_pZS*12pf3-gfp-KmFRT-3	cgaaaggcccagtcttcagggtttactcatatgaatatcc
	Up. linearized pZS*12_pZS*12pf3-gfp-KmFRT-5	ggatattcatatgagtaaaccctgaagactgggcctttcg
	Down. linearized pZS*12_pZS*12pf3-gfp-KmFRT-3	ctttctgtcgttcgactgagcctttcgttttatttg
For assembling the pZS*12*TM*^{pf3}-*qmcA-gfp-aphAIII* plasmid	Up. qmcA_pZS*12pf3-qmcA-gfp-KmFRT-5	tggatcaaagcgcaattctttatcgtaccgcagggctatc
	Down. qmcA_pZS*12pf3-qmcA-gfp-KmFRT-3	actcatgttgttgttaaattctggctgagtccgtttgttggc

	Up. linearized pZS*12_pZS*12pf3-gfp-Kmfrt-5	ggatattcatatgagtaaaccctgaagactgggcctttcg
	Down. linearized pZS*12_pZS*12pf3-gfp-Kmfrt-3	ggtacgataaagaattgcgctttgatcc
For assembling the pZS*12*TM^pf3^-SPFH^QmcA^ -gfp-aphAIII* plasmid	Up. SPFHqmcA_pZS*12pf3-SPFHqmcA-gfp-KmFRT-5	Same as Up. qmcA_pZS*12pf3-qmcA-gfp-KmFRT-5
	Down. SPFHqmcA_pZS*12pf3-SPFHqmcA-gfp-KmFRT-3	actcatgttgttgttaaattcaccttcggctttgaggatttcc
	Up. Linearised pZS*12_pZS*12pf3-SPFHqmcA-gfp-KmFRT-5	caaagccgaaggtgaatttaacaacaacatgagtaaagg
	Down. Linearised pZS*12_pZS*12pf3-SPFHqmcA-gfp-KmFRT-3	Same as Down. linearized pZS*12_pZS*12pf3-gfp-Kmfrt-3
For amplifying the DNA fragments to generate TM^{Pf3}-QmcA-GFP, TM^{Pf3}-$SPFH^{QmcA}$-GFP, and TM^{Pf3}-GFP strains	Up.Pf3_pf3-(QmcA)-GFP-KmFRT-5	caattttgctgattatggacggtacaacaggaggtttttccgatgcaatccgtgattactgatg
	Down.KmFRT_pf3-(QmcA)-GFP-KmFRT-3	Same sequence as Down.KmFRT_SPFHqmcA-GFP-KmFRT-3
For assembling the pUC18*TM^{HflC}-HflC-mCherry-aphAIII* plasmid	Up.hflC-mCherry_pUC18hflC-mCherry-KmFRT-5	gatttcttccgctacatgaagacgccgacttccgcaacgcgtggcggcggcggcagcatggtttccaagggcgaggag
	Down. hflC-mCherry_pUC18hflC-mCherry-KmFRT-3	caaaatctttctgtcgttatttgtacagctcatccatg
	Up.KmFRT_pUC18hflC-mCherry-KmFRT-5	cgacagaaagattttgggagcaaatgatgtgtgtaggc
	Down.KmFRT_pUC18hflC-mCherry-KmFRT-3	gaggatgcggtggctttattgacctgtaccgcagtcgttatatcagggtttactcatatgaatatc
	Up.linearized pUC18_ pUC18hflC-mCherry-KmFRT-5	caataaagccaccgcatcctcgaattcgtaatcatggtcatag
	Down.linearized pUC18_ pUC18hflC-mCherry-KmFRT-3	catgtagcggaagaaatcggatcctctagagtcgacctg
For amplifying the DNA fragment to generate TM^{HflC}-HflC-mCherry strain	Up.hflC_HflC-mCherry-KmFRT-5	Same as Up.hflC-mCherry_pUC18hflC-mCherry-5
	Down.KmFRT_HflC-mCherry-KmFRT-3	gaggatgcggtggctttattgacctgtaccgcagtcgttatatcagggtttactcatatgaatatc
For amplifying the DNA fragment to generate TM^{hflC}-$SPFH^{hflC}$-mCherry strain	Up.SPFHhflC_SPFHhflC-mCherry-CmFRT-5	gagcgtgaagcggtagcgcgtcgtcaccgttcacaaggtcaggaatttaacaacaacatggtttccaagg
	Down.CmFRT_SPFHhflC-mCherry-CmFRT-3	ggatgcggtggctttattgacctgtaccgcagtcgttatacatatgaatatcctccttagttcctattcc
For amplifying the DNA fragment to generate TM^{hflC}-mCherry strain	Up.TMhflC_TMhflC-mCherry-KmFRT-5	catcgtgctggtagtgctttacatgtctgtctttgtcgtcggcggcggcggcagcatg
	Down.KmFRT_TMhflC-mCherry-KmFRT-3	gaggatgcggtggctttattgacctgtaccgcagtcgttatatcagggtttactcatatgaatatc
	Up.cmi-pZS*12cmi-mcherry-5	gaaaggtaccatgaaagtgattagcatgaaatttatttttattc

For assembling the pZS*12*TM*^{cmi}-*mCherry* plasmid	Down.cmi-pZS*12cmi-mCherry-3	tggaaaccatgttgttgttaaattcgcc
	Up.mCherry_pZS*12cmi-mCherry-5	taacaacaacatggtttccaagggcgag
	Down.mCherry_pZS*12cmi-mCherry-3	ctaattaagcttatttgtacagctcatccatgc
	Up.linearized pZS*12_pZS12cmi-mCherry-5	gtacaaataagcttaattagctgagtctagagg
	Down.linearized pZS*12_pZS12cmi-mCherry-3	tcactttcatggtacctttctcctctttaatg
For assembling the pZS*12*TM*^{cmi}-*mCherry-cat* plasmid	Up.CmFRT_pZS*12cmi-mCherry-CmFRT-5	gctcagtcgagtgtaggctggagctgcttc
	Down. CmFRT_pZS*12cmi-mCherry-CmFRT-5	gcccagtcttcatatgaatatcctccttagttcctattcc
	Up.linearized pZS*12_ pZS*12cmi-mCherry-CmFRT-5	tattcatatgaagactgggcctttcgttttatc
	Down.linearized pZS*12_ pZS*12cmi-mCherry-CmFRT-3	cagcctacactcgactgagcctttcgttttatttg
For assembling the pZS*12*TM*^{cmi}-*hflC-mCherry-cat* plasmid	Up.hflC_ pZS*12cmi-mCherry-CmFRT-5	agataaaggcgtcgtcaaagaaggtgagcgc
	Down.hflC_ pZS*12cmi-mCherry-CmFRT-3	tgttaaattcacgcgttgcggaagtcgg
	Up.linearized pZS*12_ pZS*12cmi-hflC-mCherry-CmFRT-5	cgcaacgcgtgaatttaacaacaacatggtttcc
	Down.linearized pZS*12_ pZS*12cmi-hflC-mCherry-CmFRT-3	ctttgacgacgcctttatcttcgctccaaaaaaac
For assembling the pZS*12*TM*^{cmi}-*SPFH*^{HflC}-*mCherry-cat* plasmid	Up. SPFHhflC_ pZS*12cmi-SPFHhflC-mCherry-CmFRT-5	Same as Up.hflC_ pZS*12cmi-mCherry-CmFRT-5
	Down. SPFHhflC_ pZS*12cmi-SPFHhflC-mCherry-CmFRT-3	tgttaaattcctgaccttgtgaacggtg
	Up.linearized pZS*12_ pZS*12cmi-SPFHhflC-mCherry-CmFRT-5	acaaggtcaggaatttaacaacaacatggtttcc
	Down.linearized pZS*12_ pZS*12cmi-SPFHhflC-mCherry-CmFRT-5	Same as Down.linearized pZS*12_ pZS*12cmi-hflC-mCherry-CmFRT-3
For amplifying the DNA fragments to generate TM^{cmi}-HflC-mCherry, TM^{cmi}-SPFH^{HflC}-mCherry, TM^{cmi}-mCherry	Up.cmi_cmi-(HflC)-mCherry-CmFRT-5	ccaacgcgcagcgtaacgactaccagcgtcagggggaataacgatgaaagtgattagcatgaa
	Down.CmFRT_cmi-(HflC)-mCherry-CmFRT-3	ggatgcggtggctttattgacctgtaccgcagtcgttatacatatgaatatcctccttag
For amplifying the DNA fragment to generate AcrA-GFP strain	Up.acrA_AcrA-GFP-KmFRT-5	ccagcaagccgcaagcggtgctcagcctgaacagtccaagtctgaatttaacaacaacatgagtaaaggtgaagaac
	Down.KmFRT_AcrA-GFP-KmFRT-3	cgggcgatcgataaagaaattaggcatgtcttaacggctcctgtttaagtcagggtttactcatatgaatatcctcc

For assembling the pZS12**hflKC* plasmid	Up.hflKC_ pZS*12-HflKC-5	cattaaagaggagaaaggtaccatggcgtggaatcagcccggtaataacgg
	Down.hflKC_ pZS*12-HflKC -3	gatgcctctagactcagctaattaagcttttaacgcgttgcggaagtcggcg
	Up.linearized pZS*12_ pZS*12 -5	aagcttaattagctgagtctag
	Down.linearized pZS*12_ pZS*12-3	ggtacctttctcctctttaatgaattcgg
