# Supplementary material for: Escherichia coli SPFH Membrane Microdomain Proteins HflKC Contribute to Aminoglycoside and Oxidative Stress Tolerance
Source: Microbiol Spectr. 2023 Jun 22;11(4):e01767-23. doi: 10.1128/spectrum.01767-23 (PMC10434171; doi:10.1128/spectrum.01767-23)
Supplement: Supplemental file 2 — Supplemental material. Download spectrum.01767-23-s0002.pdf, PDF file, 7.3 MB [file spectrum.01767-23-s0002.pdf]

BIOLOG

Phenotype MicroArrays™

PM1 MicroPlate™ Carbon Sources

A1<br>Negative Control	A2<br>L-Arabinose	A3<br>N-Acetyl-D-Glucosamine	A4<br>D-Saccharic Acid	A5<br>Succinic Acid	A6<br>D-Galactose	A7<br>L-Aspartic Acid	A8<br>L-Proline	A9<br>D-Alanine	A10<br>D-Trehalose	A11<br>D-Mannose	A12<br>Dulcitol
B1<br>D-Serine	B2<br>D-Sorbitol	B3<br>Glycerol	B4<br>L-Fucose	B5<br>D-Glucuronic Acid	B6<br>D-Gluconic Acid	B7<br>D,L-α-Glycerol-Phosphate	B8<br>D-Xylose	B9<br>L-Lactic Acid	B10<br>Formic Acid	B11<br>D-Mannitol	B12<br>L-Glutamic Acid
C1<br>D-Glucose-6-Phosphate	C2<br>D-Galactonic Acid-γ-Lactone	C3<br>D,L-Malic Acid	C4<br>D-Ribose	C5<br>Tween 20	C6<br>L-Rhamnose	C7<br>D-Fructose	C8<br>Acetic Acid	C9<br>α-D-Glucose	C10<br>Maltose	C11<br>D-Melibiose	C12<br>Thymidine
D-1<br>L-Asparagine	D2<br>D-Aspartic Acid	D3<br>D-Glucosaminic Acid	D4<br>1,2-Propanediol	D5<br>Tween 40	D6<br>α-Keto-Glutaric Acid	D7<br>α-Keto-Butyric Acid	D8<br>α-Methyl-D-Galactoside	D9<br>α-D-Lactose	D10<br>Lactulose	D11<br>Sucrose	D12<br>Uridine
E1<br>L-Glutamine	E2<br>M-Tartaric Acid	E3<br>D-Glucose-1-Phosphate	E4<br>D-Fructose-6-Phosphate	E5<br>Tween 80	E6<br>α-Hydroxy Glutaric Acid-γ-Lactone	E7<br>α-Hydroxy Butyric Acid	E8<br>β-Methyl-D-Glucoside	E9<br>Adonitol	E10<br>Maltotriose	E11<br>2-Deoxy Adenosine	E12<br>Adenosine
F1<br>Glycyl-L-Aspartic Acid	F2<br>Citric Acid	F3<br>M-Inositol	F4<br>D-Threonine	F5<br>Fumaric Acid	F6<br>Bromo Succinic Acid	F7<br>Propionic Acid	F8<br>Mucic Acid	F9<br>Glycolic Acid	F10<br>Glyoxylic Acid	F11<br>D-Cellobiose	F12<br>Inosine
G1<br>Glycyl-L-Glutamic Acid	G2<br>Tricarballylic Acid	G3<br>L-Serine	G4<br>L-Threonine	G5<br>L-Alanine	G6<br>L-Alanyl-Glycine	G7<br>Acetoacetic Acid	G8<br>N-Acetyl-β-D-Mannosamine	G9<br>Mono Methyl Succinate	G10<br>Methyl Pyruvate	G11<br>D-Malic Acid	G12<br>L-Malic Acid
H1<br>Glycyl-L-Proline	H2<br>p-Hydroxy Phenyl Acetic Acid	H3<br>m-Hydroxy Phenyl Acetic Acid	H4<br>Tyramine	H5<br>D-Psicose	H6<br>L-Lyxose	H7<br>Glucuronamide	H8<br>Pyruvic Acid	H9<br>L-Galactonic Acid-γ-Lactone	H10<br>D-Galacturonic Acid	H11<br>Phenylethyl-amine	H12<br>2-Aminoethanol

PM2A MicroPlate™ Carbon Sources

A1<br>Negative Control	A2<br>Chondroitin Sulfate C	A3<br>α-Cyclodextrin	A4<br>β-Cyclodextrin	A5<br>γ-Cyclodextrin	A6<br>Dextrin	A7<br>Gelatin	A8<br>Glycogen	A9<br>Inulin	A10<br>Laminarin	A11<br>Mannan	A12<br>Pectin
B1<br>N-Acetyl-D-Galactosamine	B2<br>N-Acetyl-Neuraminic Acid	B3<br>β-D-Allose	B4<br>Amygdalin	B5<br>D-Arabinose	B6<br>D-Arabitol	B7<br>L-Arabitol	B8<br>Arbutin	B9<br>2-Deoxy-D-Ribose	B10<br>I-Erythritol	B11<br>D-Fucose	B12<br>3-0-β-D-Galacto-pyranosyl-D-Arabinose
C1<br>Gentiobiose	C2<br>L-Glucose	C3<br>Lactitol	C4<br>D-Melezitose	C5<br>Maltitol	C6<br>α-Methyl-D-Glucoside	C7<br>β-Methyl-D-Galactoside	C8<br>3-Methyl Glucose	C9<br>β-Methyl-D-Glucuronic Acid	C10<br>α-Methyl-D-Mannoside	C11<br>β-Methyl-D-Xyloside	C12<br>Palatinose
D1<br>D-Raffinose	D2<br>Salicin	D3<br>Sedoheptulosa n	D4<br>L-Sorbose	D5<br>Stachyose	D6<br>D-Tagatose	D7<br>Turanose	D8<br>Xylitol	D9<br>N-Acetyl-D-Glucosaminitol	D10<br>γ-Amino Butyric Acid	D11<br>δ-Amino Valeric Acid	D12<br>Butyric Acid
E1<br>Capric Acid	E2<br>Caproic Acid	E3<br>Citraconic Acid	E4<br>Citramalic Acid	E5<br>D-Glucosamine	E6<br>2-Hydroxy Benzoic Acid	E7<br>4-Hydroxy Benzoic Acid	E8<br>β-Hydroxy Butyric Acid	E9<br>γ-Hydroxy Butyric Acid	E10<br>α-Keto Valeric Acid	E11<br>Itaconic Acid	E12<br>5-Keto-D-Gluconic Acid
F1<br>D-Lactic Acid Methyl Ester	F2<br>Malonic Acid	F3<br>Melibionic Acid	F4<br>Oxalic Acid	F5<br>Oxalomalic Acid	F6<br>Quinic Acid	F7<br>D-Ribono-1,4-Lactone	F8<br>Sebacic Acid	F9<br>Sorbic Acid	F10<br>Succinamic Acid	F11<br>D-Tartaric Acid	F12<br>L-Tartaric Acid
G1<br>Acetamide	G2<br>L-Alaninamide	G3<br>N-Acetyl-L-Glutamic Acid	G4<br>L-Arginine	G5<br>Glycine	G6<br>L-Histidine	G7<br>L-Homoserine	G8<br>Hydroxy-L-Proline	G9<br>L-Isoleucine	G10<br>L-Leucine	G11<br>L-Lysine	G12<br>L-Methionine
H1<br>L-Ornithine	H2<br>L-Phenylalanine	H3<br>L-Pyroglutamic Acid	H4<br>L-Valine	H5<br>D,L-Carnitine	H6<br>Sec-Butylamine	H7<br>D.L-Octopamine	H8<br>Putrescine	H9<br>Dihydroxy Acetone	H10<br>2,3-Butanediol	H11<br>2,3-Butanone	H12<br>3-Hydroxy 2-Butanone

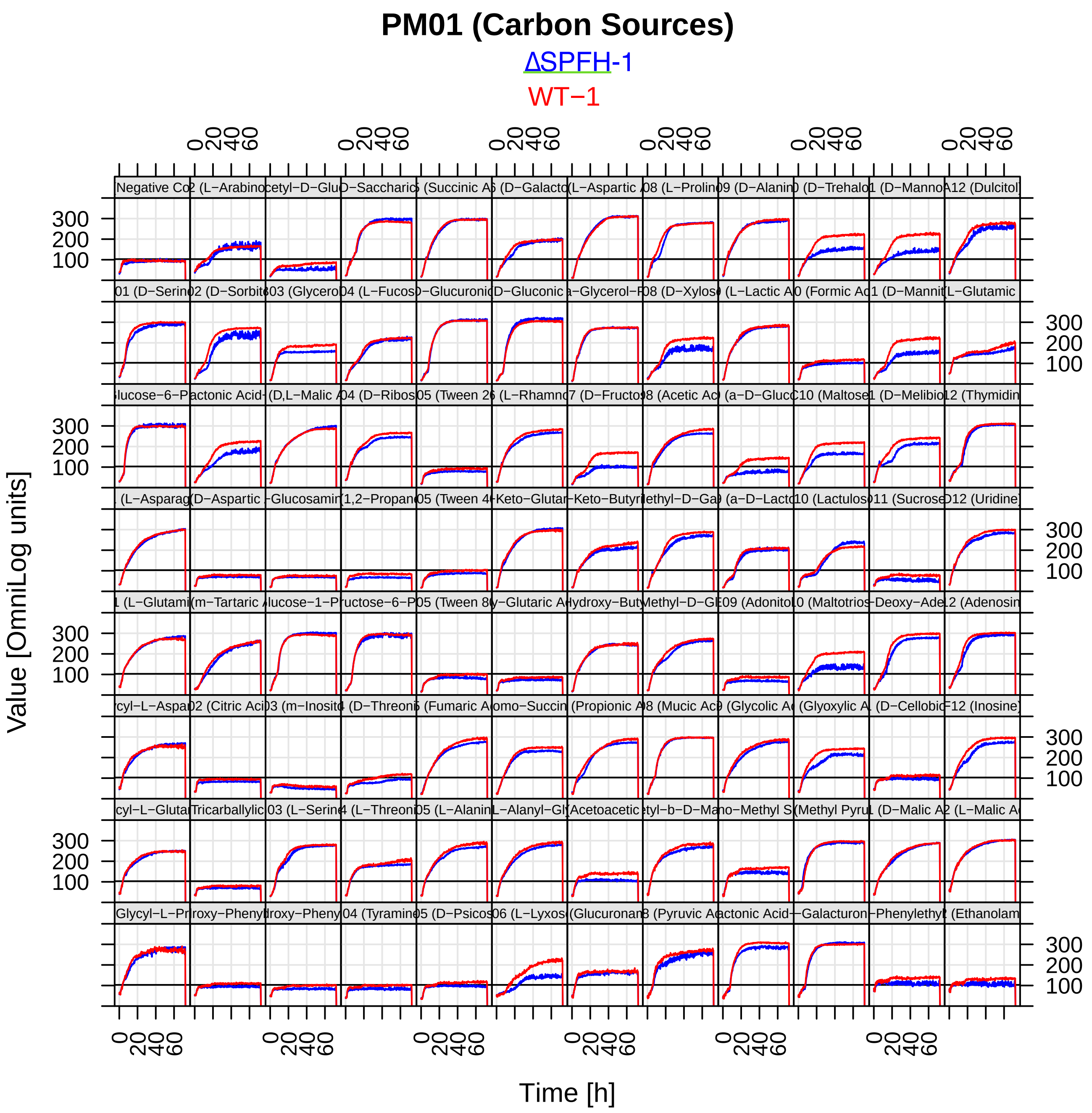
PM01 (Carbon Sources)
ΔSPFH-1
WT−1
Value [OmniLog units]
Time [h]

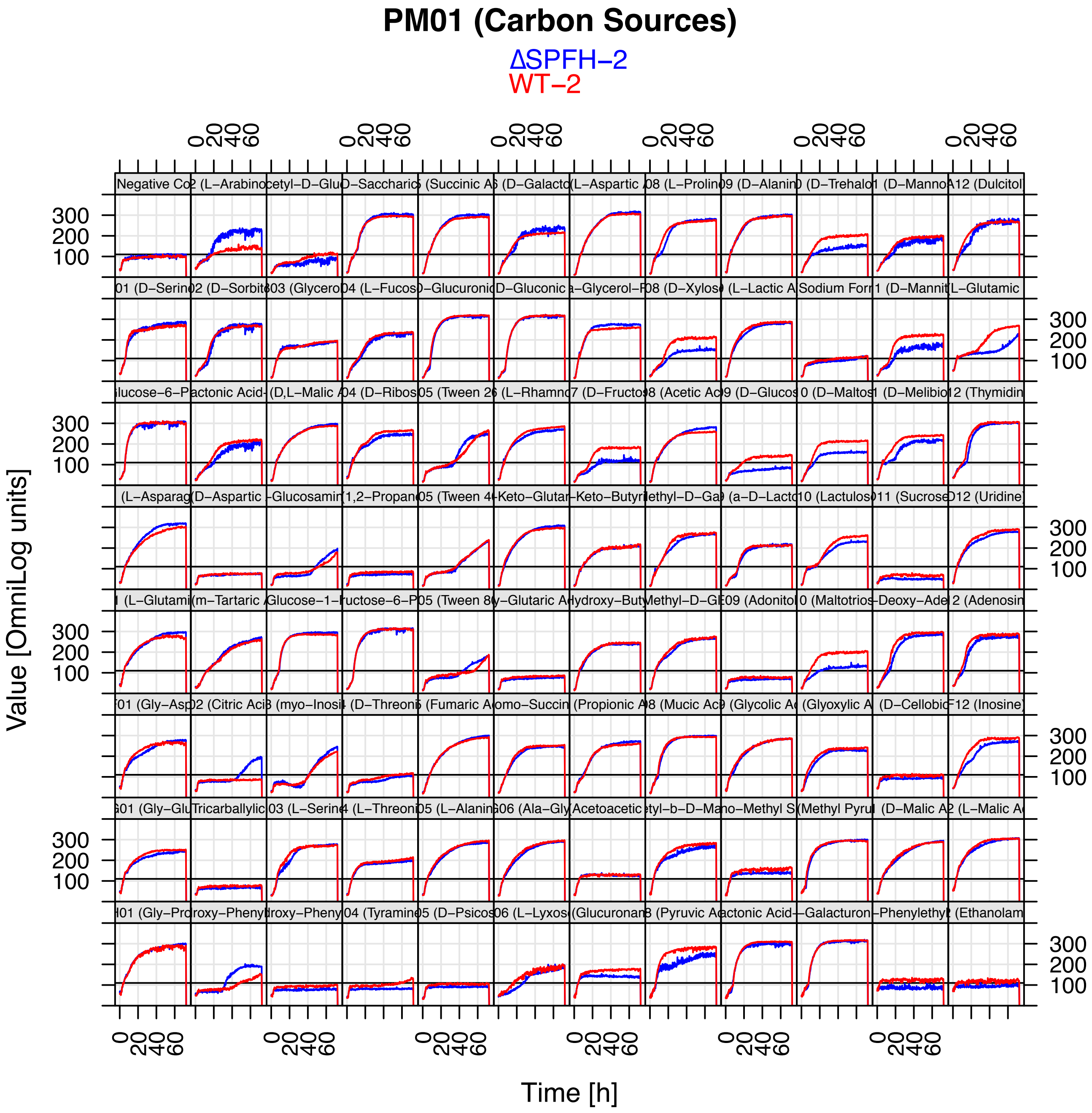
PM01 (Carbon Sources)
ΔSPFH−2
WT−2
Value [OmniLog units]
Time [h]
0
20
40
60
100
200
300
Negative Co
2 (L−Arabino
cetyl−D−Glu
D−Saccharic
(Succinic A
6 (D−Galacto
(L−Aspartic
08 (L−Prolin
09 (D−Alanin
0 (D−Trehalo
1 (D−Manno
A12 (Dulcitol
01 (D−Serin
02 (D−Sorbit
03 (Glycero
04 (L−Fucos
D−Glucuronic
D−Gluconic
a−Glycerol−P
08 (D−Xylos
(L−Lactic A
Sodium Forr
1 (D−Mannit
(L−Glutamic
lucose−6−P
actonic Acid
(D,L−Malic A
04 (D−Ribos
05 (Tween 2
6 (L−Rhamno
7 (D−Fructo
8 (Acetic Ac
9 (D−Glucos
0 (D−Maltos
1 (D−Melibio
12 (Thymidin
(L−Asparag
(D−Aspartic
Glucosamin
1,2−Propan
05 (Tween 4
Keto−Glutar
Keto−Butyr
ethyl−D−Ga
(a−D−Lacto
10 (Lactulos
011 (Sucrose
D12 (Uridine
(L−Glutami
(m−Tartaric
Glucose−1−
ructose−6−P
05 (Tween 8
y−Glutaric A
ydroxy−Buty
ethyl−D−Gl
E09 (Adonitol
0 (Maltotrios
Deoxy−Ade
2 (Adenosin
F01 (Gly−Asp
2 (Citric Aci
(myo−Inosi
(D−Threoni
(Fumaric A
omo−Succin
(Propionic A
8 (Mucic Ac
(Glycolic A
(Glyoxylic A
(D−Cellobio
F12 (Inosine
G01 (Gly−Glu
Tricarballylic
03 (L−Serin
4 (L−Threoni
05 (L−Alanin
G06 (Ala−Gly
Acetoacetic
tyl−b−D−Ma
no−Methyl S
(Methyl Pyru
(D−Malic A
2 (L−Malic A
H01 (Gly−Pro
roxy−Pheny
droxy−Pheny
04 (Tyramine
5 (D−Psicos
06 (L−Lyxos
(Glucuronam
8 (Pyruvic A
actonic Acid
Galacturon
Phenylethy
2 (Ethanolam

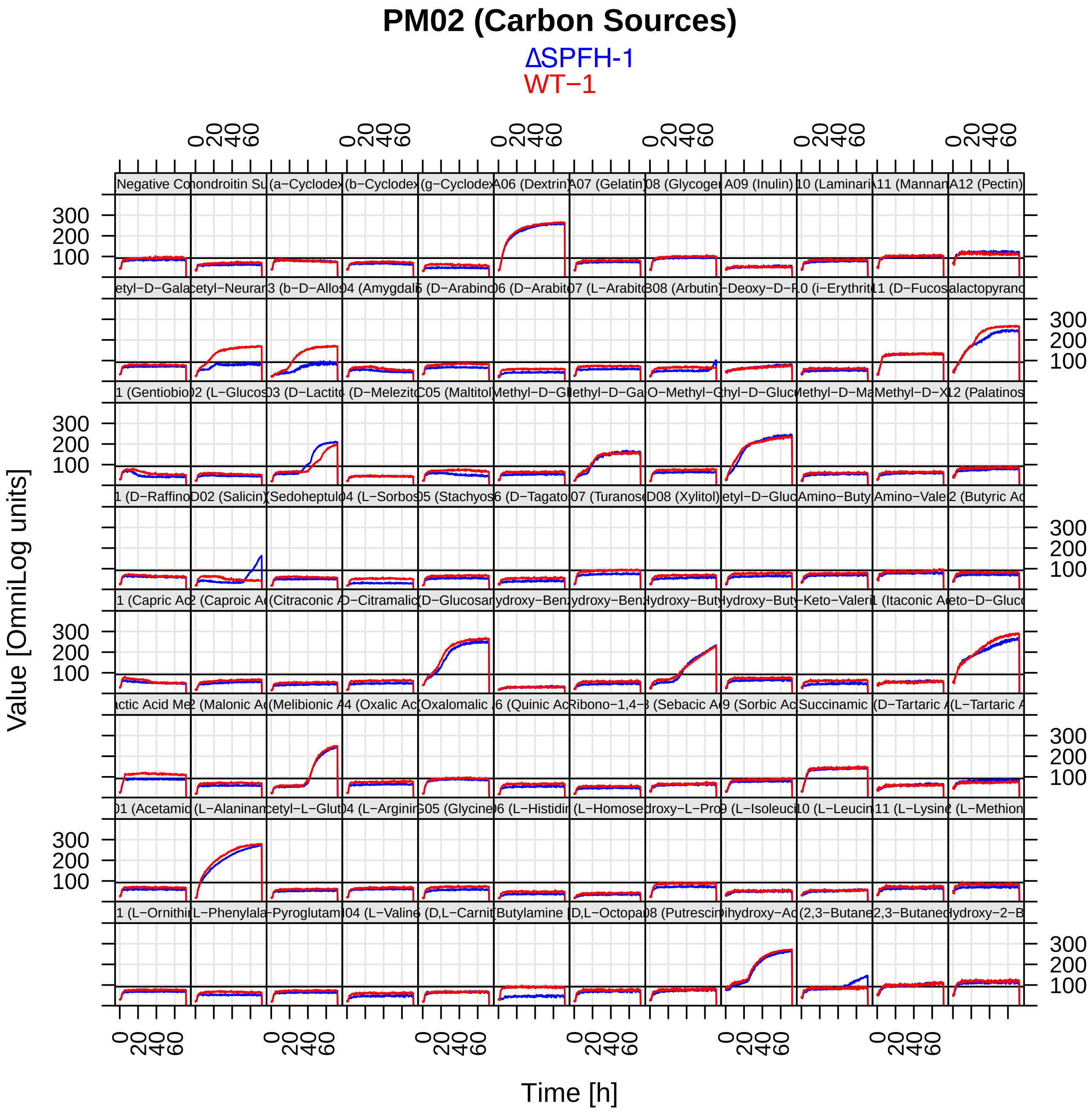
PM02 (Carbon Sources)
ΔSPFH-1
WT−1
Value [OmniLog units]
Time [h]

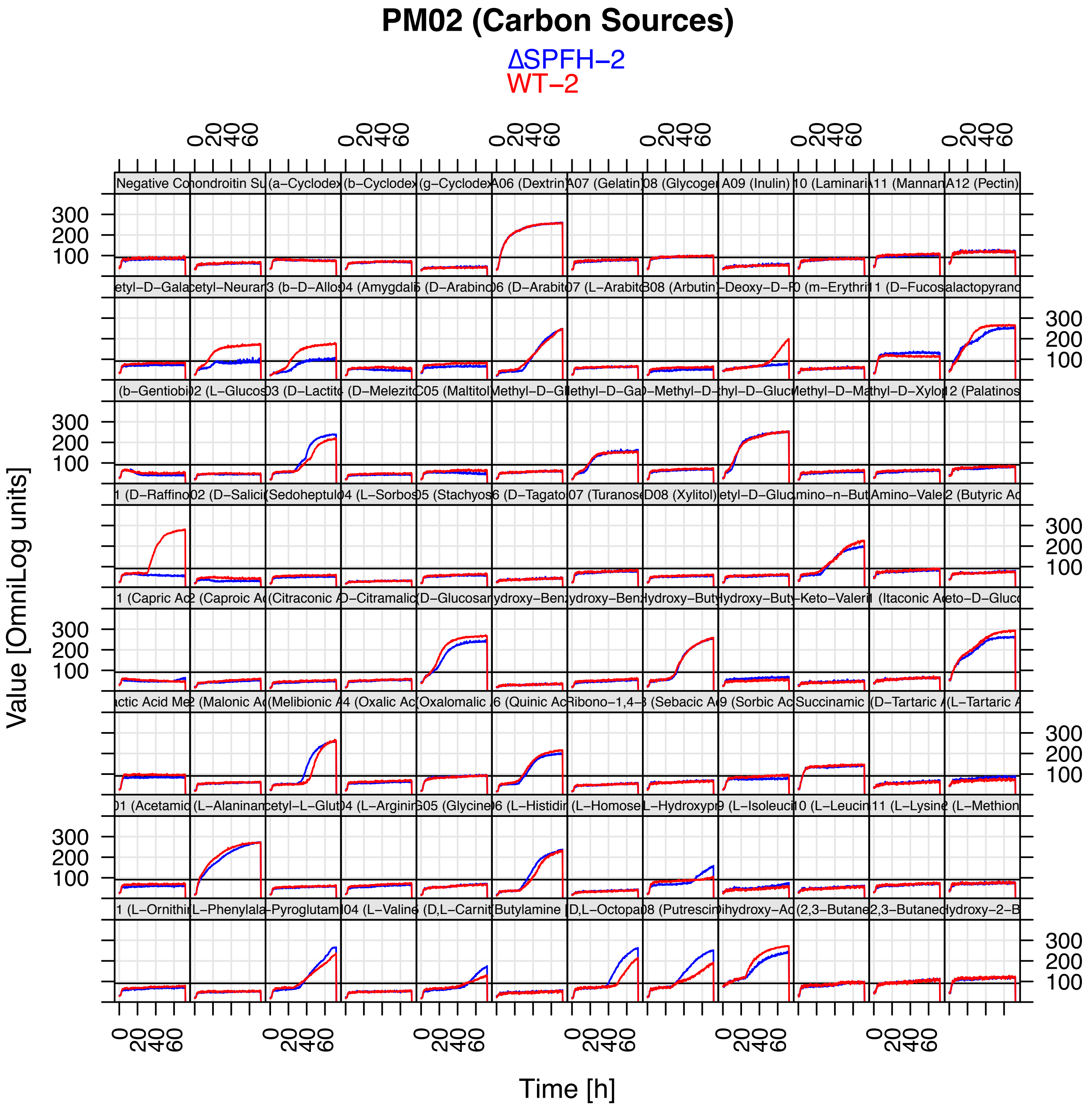
PM02 (Carbon Sources)
ΔSPFH−2
WT−2
Negative Co
ondroitin Su
(a−Cyclodex
(b−Cyclodex
(g−Cyclodex
A06 (Dextrin)
A07 (Gelatin)
08 (Glycogen
A09 (Inulin)
10 (Laminari
11 (Mannan
A12 (Pectin)
etyl−D−Gala
cetyl−Neuran
3 (b−D−Allo
04 (Amygdali
5 (D−Arabino
06 (D−Arabit
07 (L−Arabit
B08 (Arbutin)
−Deoxy−D−R
0 (m−Erythrit
1 (D−Fucos
alactopyrano
(b−Gentiobi
02 (L−Glucos
03 (D−Lactit
(D−Melezit
C05 (Maltitol
Methyl−D−Gl
ethyl−D−Ga
−Methyl−D−
thyl−D−Gluc
ethyl−D−Ma
thyl−D−Xylop
2 (Palatinos
1 (D−Raffino
02 (D−Salici
Sedoheptul
04 (L−Sorbos
5 (Stachyos
6 (D−Tagato
07 (Turanose
D08 (Xylitol)
etyl−D−Gluc
mino−n−But
Amino−Vale
2 (Butyric Ac
1 (Capric Ac
2 (Caproic Ac
(Citraconic A
D−Citramalic
(D−Glucosan
ydroxy−Benz
ydroxy−Benz
Hydroxy−Buty
ydroxy−Buty
Keto−Valeri
(Itaconic A
eto−D−Gluc
actic Acid Me
2 (Malonic Ac
(Melibionic A
4 (Oxalic Ac
Oxalomalic
6 (Quinic Ac
Ribono−1,4−
8 (Sebacic Ac
9 (Sorbic Ac
Succinamic
(D−Tartaric A
(L−Tartaric A
01 (Acetamid
(L−Alaninam
cetyl−L−Glut
04 (L−Argini
G05 (Glycine
6 (L−Histidi
(L−Homose
L−Hydroxypr
9 (L−Isoleuci
0 (L−Leucin
11 (L−Lysine
(L−Methion
1 (L−Ornithi
L−Phenylala
−Pyroglutam
04 (L−Valine
(D,L−Carnit
Butylamine
(D,L−Octopa
08 (Putresci
ihydroxy−Ac
(2,3−Butane
2,3−Butane
ydroxy−2−B
Value [OmniLog units]
Time [h]
0 20 40 60
100 200 300

BIOLOG

Phenotype MicroArrays™

PM3B MicroPlate™ Nitrogen Sources

A1 Negative Control	A2 Ammonia	A3 Nitrite	A4 Nitrate	A5 Urea	A6 Biuret	A7 L-Alanine	A8 L-Arginine	A9 L-Asparagine	A10 L-Aspartic Acid	A11 L-Cysteine	A12 L-Glutamic Acid
B1 L-Glutamine	B2 Glycine	B3 L-Histidine	B4 L-Isoleucine	B5 L-Leucine	B6 L-Lysine	B7 L-Methionine	B8 L-Phenylalanine	B9 L-Proline	B10 L-Serine	B11 L-Threonine	B12 L-Tryptophan
C1 L-Tyrosine	C2 L-Valine	C3 D-Alanine	C4 D-Asparagine	C5 D-Aspartic Acid	C6 D-Glutamic Acid	C7 D-Lysine	C8 D-Serine	C9 D-Valine	C10 L-Citrulline	C11 L-Homoserine	C12 L-Ornithine
D-1 N-Acetyl-D,L-Glutamic Acid	D2 N-Phthaloyl-L-Glutamic Acid	D3 L-Pyroglutamic Acid	D4 Hydroxylamine	D5 Methylamine	D6 N-Amylamine	D7 N-Butylamine	D8 Ethylamine	D9 Ethanolamine	D10 Ethylenediamine	D11 Putrescine	D12 Agmatine
E1 Histamine	E2 β-Phenylethyl-amine	E3 Tyramine	E4 Acetamide	E5 Formamide	E6 Glucuronamide	E7 D,L-Lactamide	E8 D-Glucosamine	E9 D-Galactosamine	E10 D-Mannosamine	E11 N-Acetyl-D-Glucosamine	E12 N-Acetyl-D-Galactosamine
F1 N-Acetyl-D-Mannosamine	F2 Adenine	F3 Adenosine	F4 Cytidine	F5 Cytosine	F6 Guanine	F7 Guanosine	F8 Thymine	F9 Thymidine	F10 Uracil	F11 Uridine	F12 Inosine
G1 Xanthine	G2 Xanthosine	G3 Uric Acid	G4 Alloxan	G5 Allantoin	G6 Parabanic Acid	G7 D,L-α-Amino-N-Butyric Acid	G8 γ-Amino-N-Butyric Acid	G9 ε-Amino-N-Caproic Acid	G10 D,L-α-Amino-Caprylic Acid	G11 δ-Amino-N-Valeric Acid	G12 α-Amino-N-Valeric Acid
H1 Ala-Asp	H2 Ala-Gln	H3 Ala-Glu	H4 Ala-Gly	H5 Ala-His	H6 Ala-Leu	H7 Ala-Thr	H8 Gly-Asn	H9 Gly-Gln	H10 Gly-Glu	H11 Gly-Met	H12 Met-Ala

PM4A MicroPlate™ Phosphorus and Sulfur Sources

A1 Negative Control	A2 Phosphate	A3 Pyrophosphate	A4 Trimeta-phosphate	A5 Tripoly-phosphate	A6 Triethyl Phosphate	A7 Hypophosphite	A8 Adenosine- 2'-monophosphate	A9 Adenosine- 3'-monophosphate	A10 Adenosine- 5'-monophosphate	A11 Adenosine-2',3'-cyclic monophosphate	A12 Adenosine-3',5'-cyclic monophosphate
B1 Thiophosphate	B2 Dithiophosphate	B3 D,L-α-Glycerol Phosphate	B4 β-Glycerol Phosphate	B5 Carbamyl Phosphate	B6 D-2-Phospho-Glyceric Acid	B7 D-3-Phospho-Glyceric Acid	B8 Guanosine- 2'-monophosphate	B9 Guanosine- 3'-monophosphate	B10 Guanosine- 5'-monophosphate	B11 Guanosine-2',3'-cyclic monophosphate	B12 Guanosine-3',5'-cyclic monophosphate
C1 Phosphoenol Pyruvate	C2 Phospho-Glycolic Acid	C3 D-Glucose-1-Phosphate	C4 D-Glucose-6-Phosphate	C5 2-Deoxy-D-Glucose 6-Phosphate	C6 D-Glucosamine-6-Phosphate	C7 6-Phospho-Gluconic Acid	C8 Cytidine- 2'-monophosphate	C9 Cytidine- 3'-monophosphate	C10 Cytidine- 5'-monophosphate	C11 Cytidine- 2',3'-cyclic monophosphate	C12 Cytidine- 3',5'-cyclic monophosphate
D1 D-Mannose-1-Phosphate	D2 D-Mannose-6-Phosphate	D3 Cysteamine-S-Phosphate	D4 Phospho-L-Arginine	D5 O-Phospho-D-Serine	D6 O-Phospho-L-Serine	D7 O-Phospho-L-Threonine	D8 Uridine- 2'-monophosphate	D9 Uridine- 3'-monophosphate	D10 Uridine- 5'-monophosphate	D11 Uridine- 2',3'-cyclic monophosphate	D12 Uridine- 3',5'-cyclic monophosphate
E1 O-Phospho-D-Tyrosine	E2 O-Phospho-L-Tyrosine	E3 Phosphocreatine	E4 Phosphoryl Choline	E5 O-Phosphoryl-Ethanolamine	E6 Phosphono Acetic Acid	E7 2-Aminoethyl Phosphonic Acid	E8 Methylene Diphosphonic Acid	E9 Thymidine- 3'-monophosphate	E10 Thymidine- 5'-monophosphate	E11 Inositol Hexaphosphate	E12 Thymidine 3',5'- cyclic monophosphate
F1 Negative Control	F2 Sulfate	F3 Thiosulfate	F4 Tetrathionate	F5 Thiophosphate	F6 Dithiophosphate	F7 L-Cysteine	F8 D-Cysteine	F9 L-Cysteinyl-Glycine	F10 L-Cysteic Acid	F11 Cysteamine	F12 L-Cysteine Sulfinic Acid
G1 N-Acetyl-L-Cysteine	G2 S-Methyl-L-Cysteine	G3 Cystathionine	G4 Lanthionine	G5 Glutathione	G6 D,L-Ethionine	G7 L-Methionine	G8 D-Methionine	G9 Glycyl-L-Methionine	G10 N-Acetyl-D,L-Methionine	G11 L- Methionine Sulfoxide	G12 L-Methionine Sulfone
H1 L-Djenkolic Acid	H2 Thiourea	H3 1-Thio-β-D-Glucose	H4 D,L-Lipoamide	H5 Taurocholic Acid	H6 Taurine	H7 Hypotaurine	H8 p-Amino Benzene Sulfonic Acid	H9 Butane Sulfonic Acid	H10 2-Hydroxyethane Sulfonic Acid	H11 Methane Sulfonic Acid	H12 Tetramethylene Sulfone

PM03 (Nitrogen Sources)

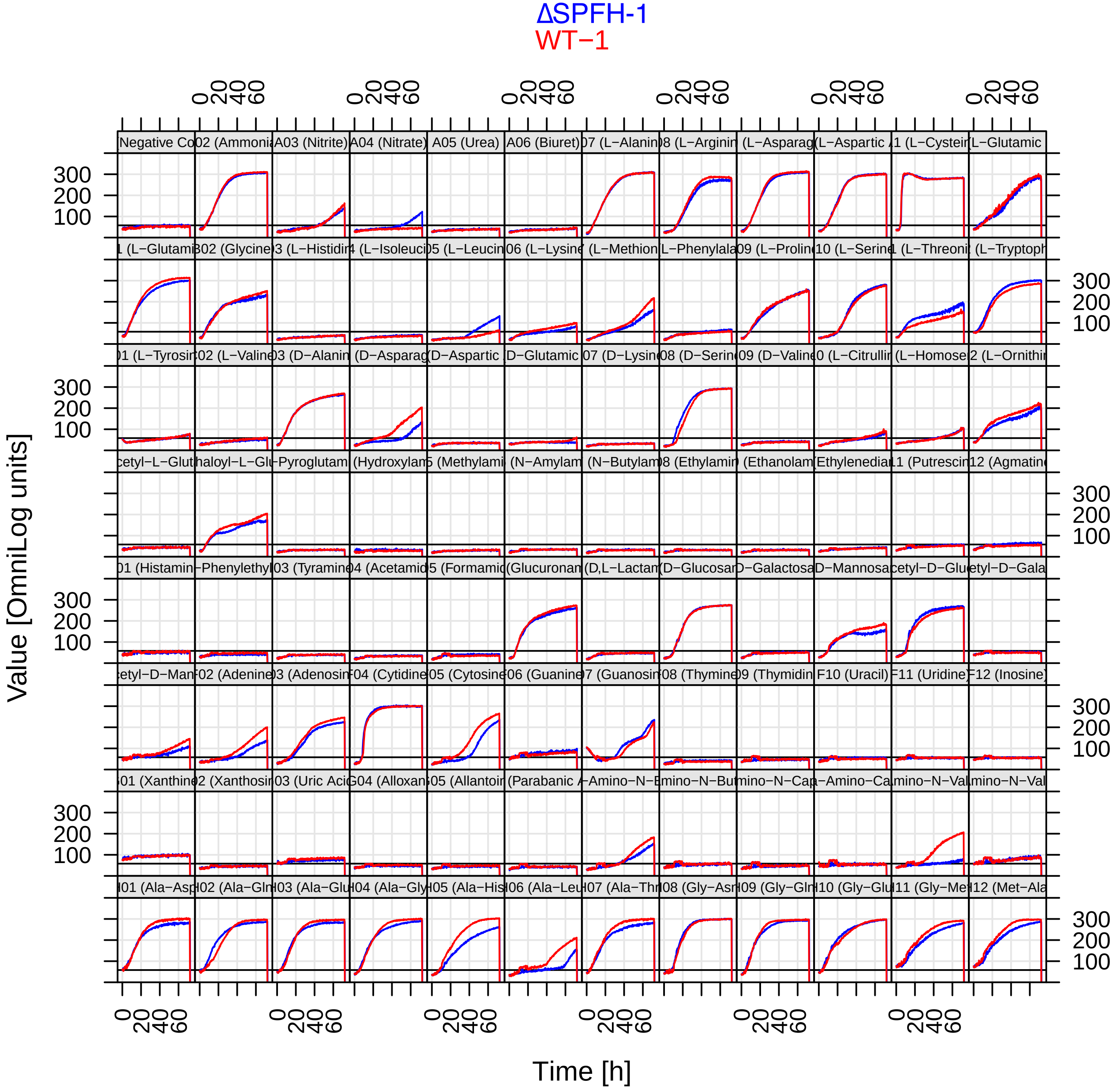

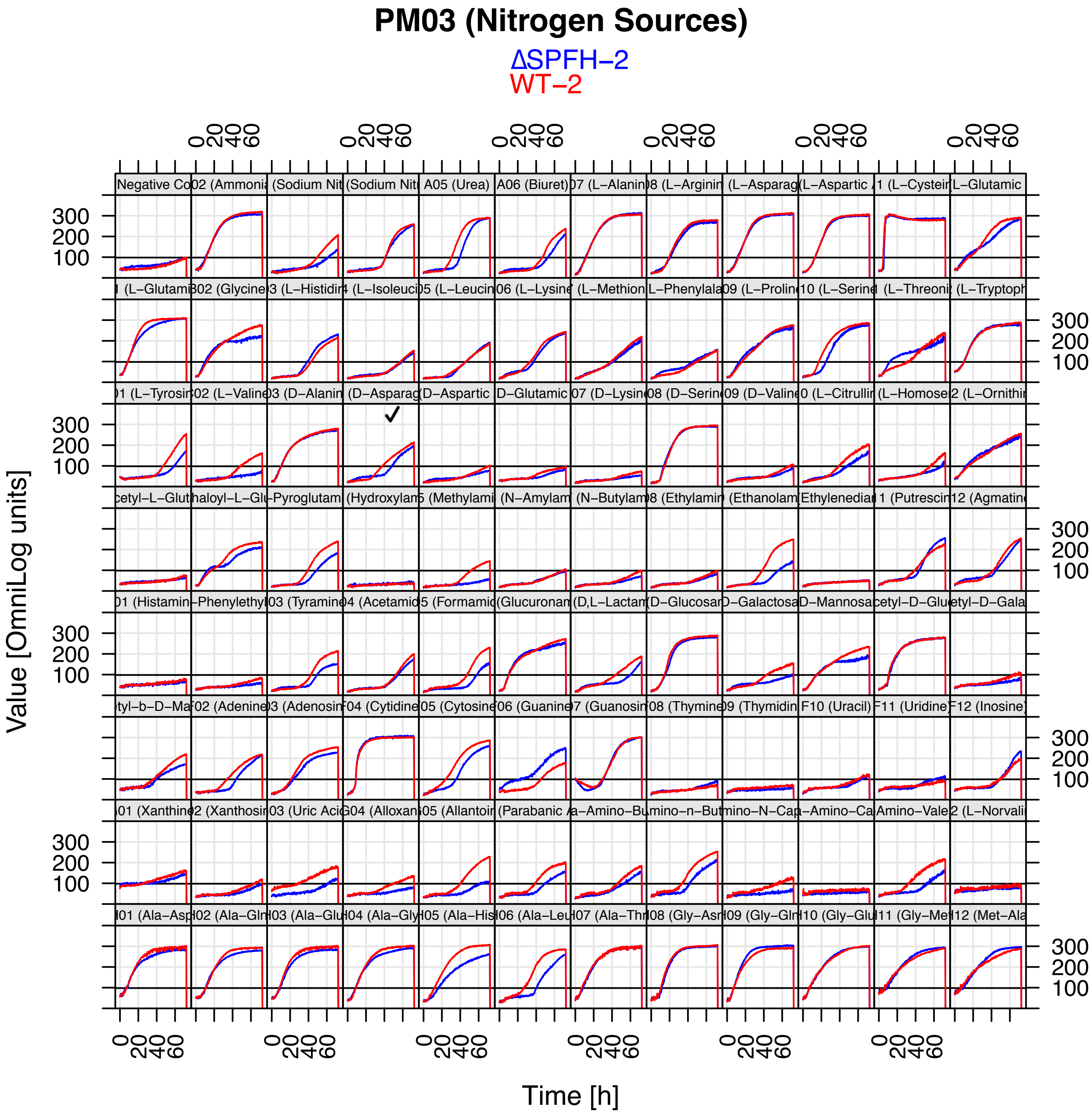
PM03 (Nitrogen Sources)
ΔSPFH−2
WT−2
Value [OmniLog units]
Time [h]

Phenotype MicroArrays™

PM9 MicroPlate™ Osmolytes

A1 NaCl 1%	A2 NaCl 2%	A3 NaCl 3%	A4 NaCl 4%	A5 NaCl 5%	A6 NaCl 5.5%	A7 NaCl 6%	A8 NaCl 6.5%	A9 NaCl 7%	A10 NaCl 8%	A11 NaCl 9%	A12 NaCl 10%
B1 NaCl 6%	B2 NaCl 6% + Betaine	B3 NaCl 6% + N-N Dimethyl glycine	B4 NaCl 6% + Sarcosine	B5 NaCl 6% + Dimethyl sulphonyl propionate	B6 NaCl 6% + MOPS	B7 NaCl 6% + Ectoine	B8 NaCl 6% + Choline	B9 NaCl 6% + Phosphoryl choline	B10 NaCl 6% + Creatine	B11 NaCl 6% + Creatinine	B12 NaCl 6% + L- Carnitine
C1 NaCl 6% + KCl	C2 NaCl 6% + L-proline	C3 NaCl 6% + N-Acethyl L-glutamine	C4 NaC1 6% + β-Glutamic acid	C5 NaC1 6% + γ–Amino -n-butyric acid	C6 NaC1 6% + Glutathione	C7 NaCl 6% + Glycerol	C8 NaC1 6% + Trehalose	C9 NaC1 6% + Trimethylamine -N-oxide	C10 NaC1 6% + Trimethylamine	C11 NaCl 6% + Octopine	C12 NaC1 6% + Trigonelline
D-1 Potassium chloride 3%	D2 Potassium chloride 4%	D3 Potassium chloride 5%	D4 Potassium chloride 6%	D5 Sodium sulfate 2%	D6 Sodium sulfate 3%	D7 Sodium sulfate 4%	D8 Sodium sulfate 5%	D9 Ethylene glycol 5%	D10 Ethylene glycol 10%	D11 Ethylene glycol 15%	D12 Ethylene glycol 20%
E1 Sodium formate 1%	E2 Sodium formate 2%	E3 Sodium formate 3%	E4 Sodium formate 4%	E5 Sodium formate 5%	E6 Sodium formate 6%	E7 Urea 2%	E8 Urea 3%	E9 Urea 4%	E10 Urea 5%	E11 Urea 6%	E12 Urea 7%
F1 Sodium Lactate 1%	F2 Sodium Lactate 2%	F3 Sodium Lactate 3%	F4 Sodium Lactate 4%	F5 Sodium Lactate 5%	F6 Sodium Lactate 6%	F7 Sodium Lactate 7%	F8 Sodium Lactate 8%	F9 Sodium Lactate 9%	F10 Sodium Lactate 10%	F11 Sodium Lactate 11%	F12 Sodium Lactate 12%
G1 Sodium Phosphate pH 7 20mM	G2 Sodium Phosphate pH 7 50mM	G3 Sodium Phosphate pH 7 100mM	G4 Sodium Phosphate pH 7 200mM	G5 Sodium Benzoate pH 5.2 20mM	G6 Sodium Benzoate pH 5.2 50mM	G7 Sodium Benzoate pH5.2 100mM	G8 Sodium Benzoate pH 5.2 200mM	G9 Ammonium sulfate pH8 10mM	G10 Ammonium sulfate pH 8 20mM	G11 Ammonium sulfate pH 8 50mM	G12 Ammonium sulfate pH8 100mM
H1 Sodium Nitrate 10mM	H2 Sodium Nitrate 20mM	H3 Sodium Nitrate 40mM	H4 Sodium Nitrate 60mM	H5 Sodium Nitrate 80mM	H6 Sodium Nitrate 100mM	H7 Sodium Nitrite 10mM	H8 Sodium Nitrite 20mM	H9 Sodium Nitrite 40mM	H10 Sodium Nitrite 60mM	H11 Sodium Nitrite 80mM	H12 Sodium Nitrite 100mM

PM10 MicroPlate™ pH

A1 pH 3.5	A2 pH 4	A3 pH 4.5	A4 pH 5	A5 pH 5.5	A6 pH 6	A7 pH 7	A8 pH 8	A9 pH 8.5	A10 pH 9	A11 pH 9.5	A12 pH 10
B1 pH 4.5	B2 pH 4.5 + L-Alanine	B3 pH 4.5 + L-Arginine	B4 pH 4.5 + L-Asparagine	B5 pH 4.5 + L-Aspartic Acid	B6 pH 4.5 + L-Glutamic Acid	B7 pH 4.5 + L-Glutamine	B8 pH 4.5 + Glycine	B9 pH 4.5 + L-Histidine	B10 pH 4.5 + L-Isoleucine	B11 pH 4.5 + L-Leucine	B12 pH 4.5 + L-Lysine
C1 pH 4.5 + L-Methionine	C2 pH 4.5 + L-Phenylalanine	C3 pH 4.5 + L-Proline	C4 pH 4.5 + L-Serine	C5 pH 4.5 + L-Threonine	C6 pH 4.5 + L-Tryptophan	C7 pH 4.5 + L-Tyrosine	C8 pH 4.5 + L-Valine	C9 pH 4.5 + Hydroxy-L-Proline	C10 pH 4.5 + L-Ornithine	C11 pH 4.5 + L-Homoarginine	C12 pH 4.5 + L-Homoserine
D-1 pH 4.5 + Anthranilic acid	D2 pH 4.5 + L-Norleucine	D3 pH 4.5 + L-Norvaline	D4 pH 4.5 + α- Amino-N-butyric acid	D5 pH 4.5 + p-Aminobenzoate	D6 pH 4.5 + L-Cysteic acid	D7 pH 4.5 + D-Lysine	D8 pH 4.5 + 5-Hydroxy Lysine	D9 pH 4.5 + 5-Hydroxy Tryptophan	D10 pH 4.5 + D,L-Diamino pimelic acid	D11 pH 4.5 + Trimethyl amine-N-oxide	D12 pH 4.5 + Urea
E1 pH 9.5	E2 pH 9.5 + L-Alanine	E3 pH 9.5 + L-Arginine	E4 pH 9.5 + L-Asparagine	E5 pH 9.5 + L-Aspartic Acid	E6 pH 9.5 + L-Glutamic Acid	E7 pH 9.5 + L-Glutamine	E8 pH 9.5 + Glycine	E9 pH 9.5 + L-Histidine	E10 pH 9.5 + L-Isoleucine	E11 pH 9.5 + L-Leucine	E12 pH 9.5 + L-Lysine
F1 pH 9.5 + L-Methionine	F2 pH 9.5 + L-Phenylalanine	F3 pH 9.5 + L-Proline	F4 pH 9.5 + L-Serine	F5 pH 9.5 + L-Threonine	F6 pH 9.5 + L-Tryptophan	F7 pH 9.5 + L-Tyrosine	F8 pH 9.5 + L-Valine	F9 pH 9.5 + Hydroxy-L-Proline	F10 pH 9.5 + L-Ornithine	F11 pH 9.5 + L-Homoarginine	F12 pH 9.5 + L-Homoserine
G1 pH 9.5 + Anthranilic acid	G2 pH 9.5 + L-Norleucine	G3 pH 9.5 + L-Norvaline	G4 pH 9.5 + Agmatine	G5 pH 9.5 + Cadaverine	G6 pH 9.5 + Putrescine	G7 pH 9.5 + Histamine	G8 pH 9.5 + Phenylethylamine	G9 pH 9.5 + Tyramine	G10 pH 9.5 + Creatine	G11 pH 9.5 + Trimethyl amine-N-oxide	G12 pH 9.5 + Urea
H1 X-Caprylate	H2 X–α-D-Glucoside	H3 X-β-D-Glucoside	H4 X-α-D-Galactoside	H5 X-β-D-Galactoside	H6 X-α- D-Glucuronide	H7 X-β- D-Glucuronide	H8 X-β-D-Glucosaminide	H9 X-β-D-Galactosaminide	H10 X-α-D-Mannoside	H11 X-PO4	H12 X-SO4

PM09 (Osmolytes)

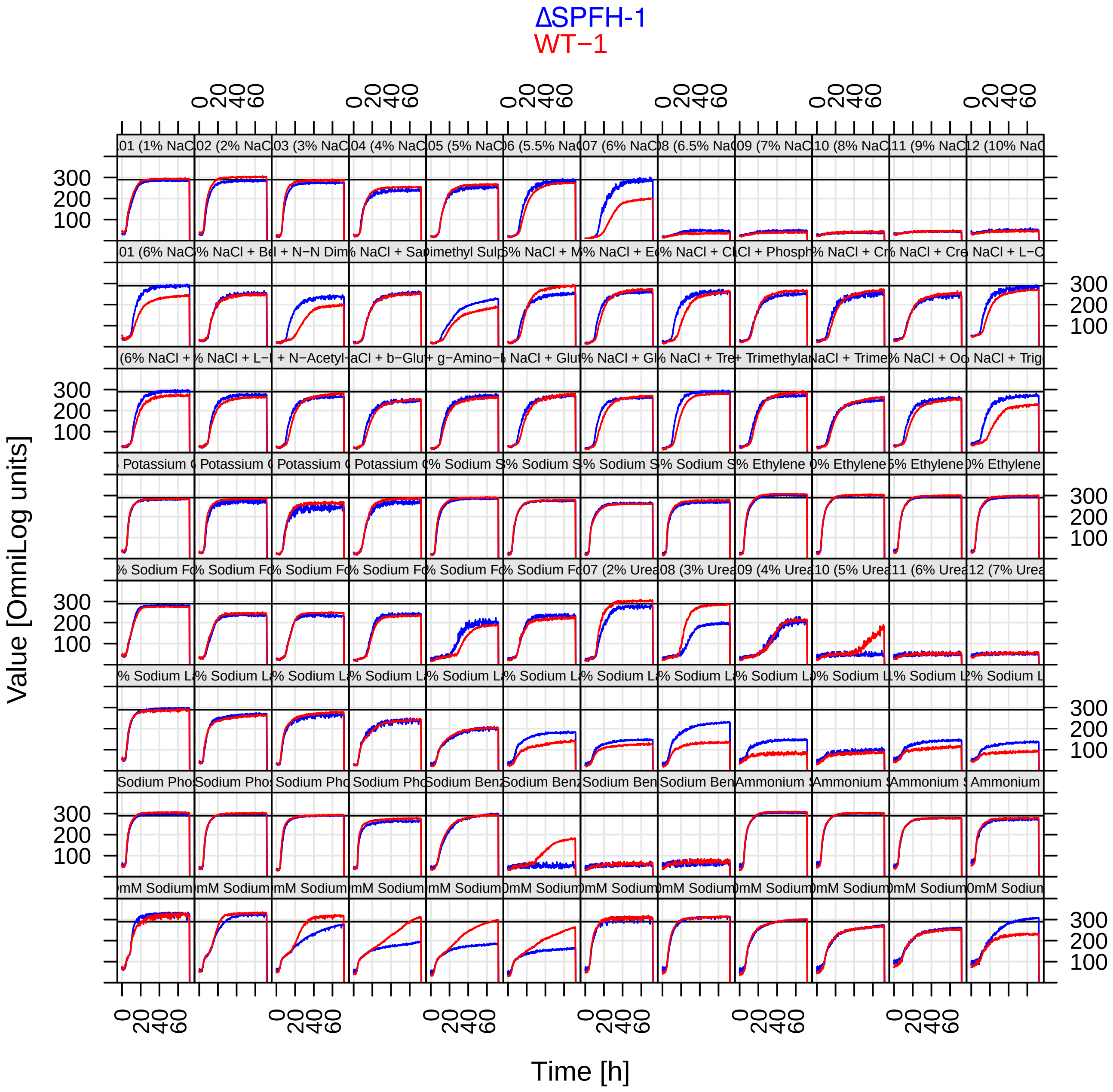

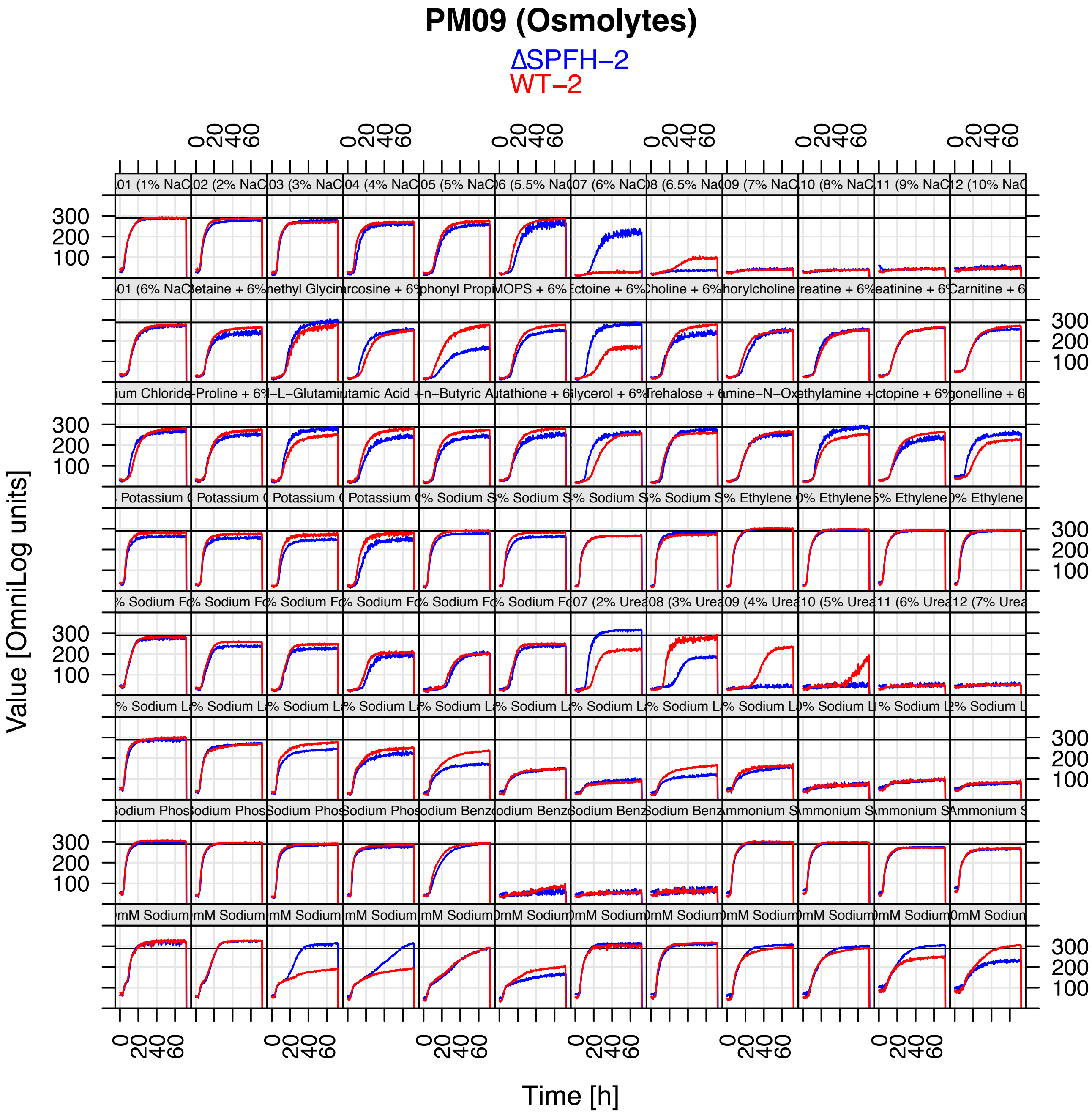
PM09 (Osmolytes)
ΔSPFH−2
WT−2
Value [OmniLog units]
Time [h]

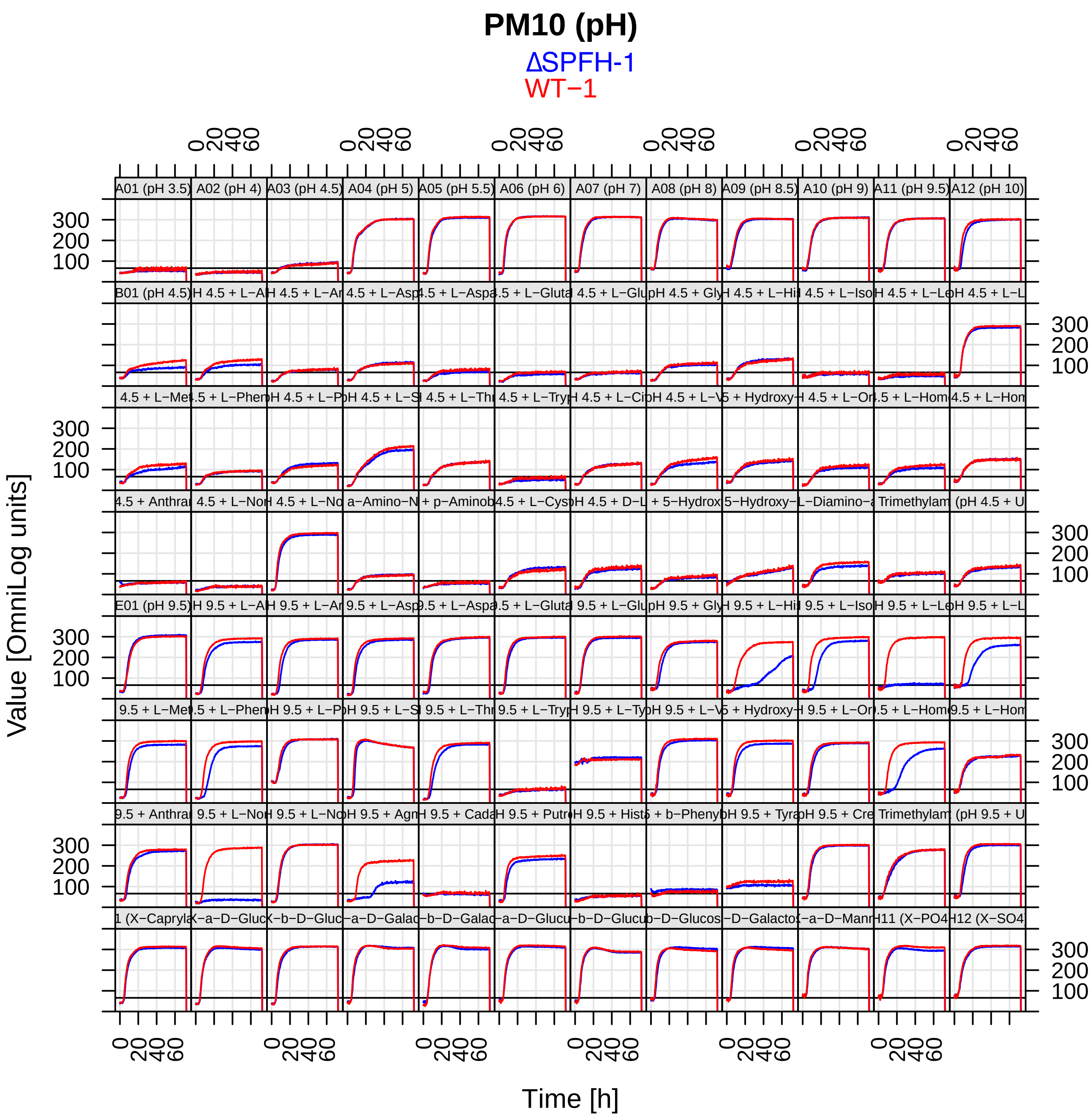
PM10 (pH)
ΔSPFH-1
WT−1
Value [OmniLog units]
Time [h]

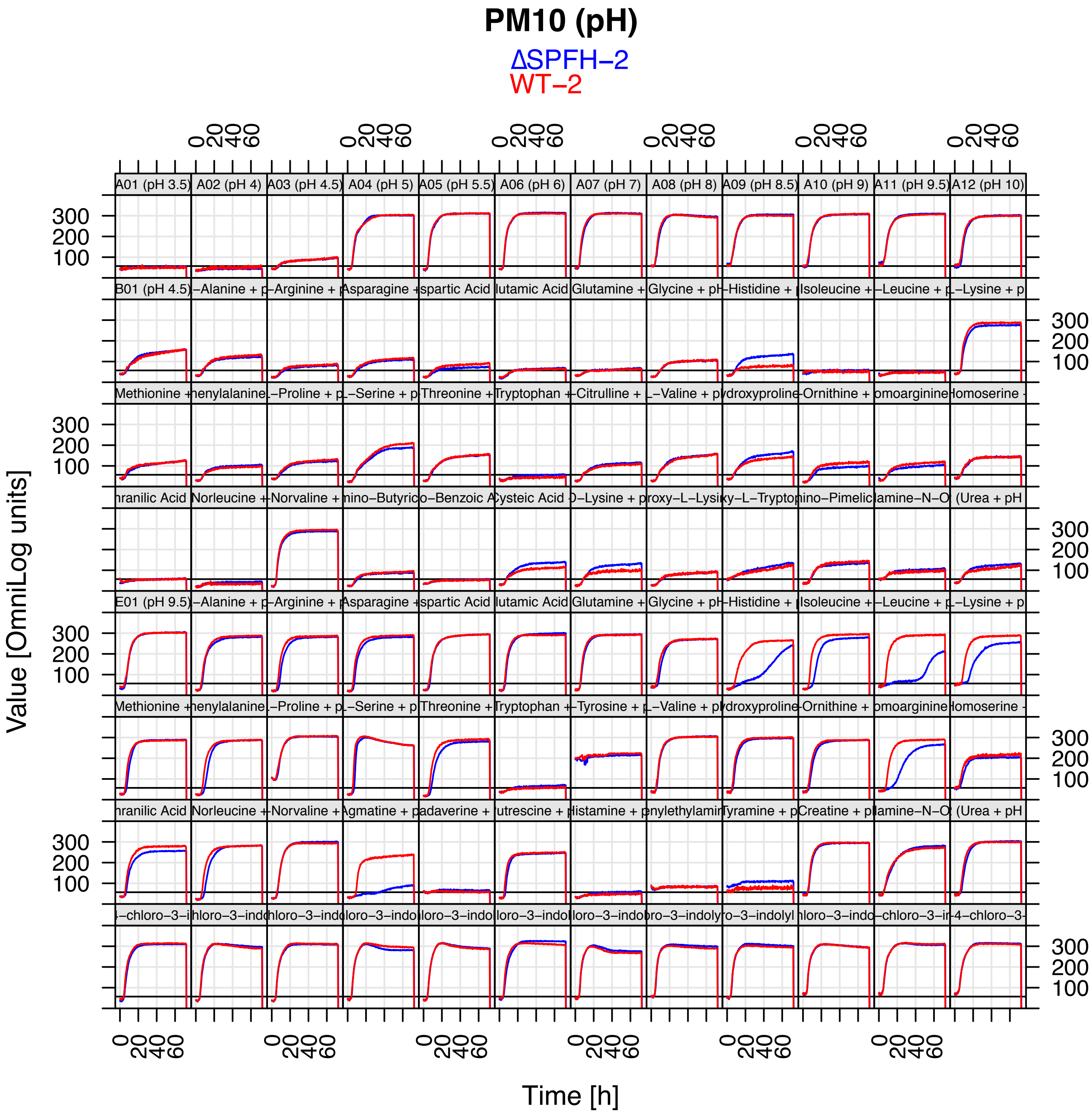
PM10 (pH)
ΔSPFH−2
WT−2
A01 (pH 3.5)
A02 (pH 4)
A03 (pH 4.5)
A04 (pH 5)
A05 (pH 5.5)
A06 (pH 6)
A07 (pH 7)
A08 (pH 8)
A09 (pH 8.5)
A10 (pH 9)
A11 (pH 9.5)
A12 (pH 10)
B01 (pH 4.5)
E01 (pH 9.5)
Value [OmniLog units]
Time [h]
0
20
40
60
100
200
300

PM11C MicroPlate™

A1<br>Amikacin<br>1	A2<br>Amikacin<br>2	A3<br>Amikacin<br>3	A4<br>Amikacin<br>4	A5<br>Chlortetracycline<br>1	A6<br>Chlortetracycline<br>2	A7<br>Chlortetracycline<br>3	A8<br>Chlortetracycline<br>4	A9<br>Lincomycin<br>1	A10<br>Lincomycin<br>2	A11<br>Lincomycin<br>3	A12<br>Lincomycin<br>4
B1<br>Amoxicillin<br>1	B2<br>Amoxicillin<br>2	B3<br>Amoxicillin<br>3	B4<br>Amoxicillin<br>4	B5<br>Cloxacillin<br>1	B6<br>Cloxacillin<br>2	B7<br>Cloxacillin<br>3	B8<br>Cloxacillin<br>4	B9<br>Lomefloxacin<br>1	B10<br>Lomefloxacin<br>2	B11<br>Lomefloxacin<br>3	B12<br>Lomefloxacin<br>4
C1<br>Bleomycin<br>1	C2<br>Bleomycin<br>2	C3<br>Bleomycin<br>3	C4<br>Bleomycin<br>4	C5<br>Colistin<br>1	C6<br>Colistin<br>2	C7<br>Colistin<br>3	C8<br>Colistin<br>4	C9<br>Minocycline<br>1	C10<br>Minocycline<br>2	C11<br>Minocycline<br>3	C12<br>Minocycline<br>4
D1<br>Capreomycin<br>1	D2<br>Capreomycin<br>2	D3<br>Capreomycin<br>3	D4<br>Capreomycin<br>4	D5<br>Demeclocycline<br>1	D6<br>Demeclocycline<br>2	D7<br>Demeclocycline<br>3	D8<br>Demeclocycline<br>4	D9<br>Nafcillin<br>1	D10<br>Nafcillin<br>2	D11<br>Nafcillin<br>3	D12<br>Nafcillin<br>4
E1<br>Cefazolin<br>1	E2<br>Cefazolin<br>2	E3<br>Cefazolin<br>3	E4<br>Cefazolin<br>4	E5<br>Enoxacin<br>1	E6<br>Enoxacin<br>2	E7<br>Enoxacin<br>3	E8<br>Enoxacin<br>4	E9<br>Nalidixic acid<br>1	E10<br>Nalidixic acid<br>2	E11<br>Nalidixic acid<br>3	E12<br>Nalidixic acid<br>4
F1<br>Chloramphenicol<br>1	F2<br>Chloramphenicol<br>2	F3<br>Chloramphenicol<br>3	F4<br>Chloramphenicol<br>4	F5<br>Erythromycin<br>1	F6<br>Erythromycin<br>2	F7<br>Erythromycin<br>3	F8<br>Erythromycin<br>4	F9<br>Neomycin<br>1	F10<br>Neomycin<br>2	F11<br>Neomycin<br>3	F12<br>Neomycin<br>4
G1<br>Ceftriaxone<br>1	G2<br>Ceftriaxone<br>2	G3<br>Ceftriaxone<br>3	G4<br>Ceftriaxone<br>4	G5<br>Gentamicin<br>1	G6<br>Gentamicin<br>2	G7<br>Gentamicin<br>3	G8<br>Gentamicin<br>4	G9<br>Potassium tellurite<br>1	G10<br>Potassium tellurite<br>2	G11<br>Potassium tellurite<br>3	G12<br>Potassium tellurite<br>4
H1<br>Cephalothin<br>1	H2<br>Cephalothin<br>2	H3<br>Cephalothin<br>3	H4<br>Cephalothin<br>4	H5<br>Kanamycin<br>1	H6<br>Kanamycin<br>2	H7<br>Kanamycin<br>3	H8<br>Kanamycin<br>4	H9<br>Ofloxacin<br>1	H10<br>Ofloxacin<br>2	H11<br>Ofloxacin<br>3	H12<br>Ofloxacin<br>4

PM12B MicroPlate™

A1<br>Penicillin G<br>1	A2<br>Penicillin G<br>2	A3<br>Penicillin G<br>3	A4<br>Penicillin G<br>4	A5<br>Tetracycline<br>1	A6<br>Tetracycline<br>2	A7<br>Tetracycline<br>3	A8<br>Tetracycline<br>4	A9<br>Carbenicillin<br>1	A10<br>Carbenicillin<br>2	A11<br>Carbenicillin<br>3	A12<br>Carbenicillin<br>4
B1<br>Oxacillin<br>1	B2<br>Oxacillin<br>2	B3<br>Oxacillin<br>3	B4<br>Oxacillin<br>4	B5<br>Penimepicycline<br>1	B6<br>Penimepicycline<br>2	B7<br>Penimepicycline<br>3	B8<br>Penimepicycline<br>4	B9<br>Polymyxin B<br>1	B10<br>Polymyxin B<br>2	B11<br>Polymyxin B<br>3	B12<br>Polymyxin B<br>4
C1<br>Paromomycin<br>1	C2<br>Paromomycin<br>2	C3<br>Paromomycin<br>3	C4<br>Paromomycin<br>4	C5<br>Vancomycin<br>1	C6<br>Vancomycin<br>2	C7<br>Vancomycin<br>3	C8<br>Vancomycin<br>4	C9<br>D,L-Serine hydroxamate<br>1	C10<br>D,L-Serine hydroxamate<br>2	C11<br>D,L-Serine hydroxamate<br>3	C12<br>D,L-Serine hydroxamate<br>4
D1<br>Sisomicin<br>1	D2<br>Sisomicin<br>2	D3<br>Sisomicin<br>3	D4<br>Sisomicin<br>4	D5<br>Sulfamethazine<br>1	D6<br>Sulfamethazine<br>2	D7<br>Sulfamethazine<br>3	D8<br>Sulfamethazine<br>4	D9<br>Novobiocin<br>1	D10<br>Novobiocin<br>2	D11<br>Novobiocin<br>3	D12<br>Novobiocin<br>4
E1<br>2,4-Diamino-6,7-diisopropyl-pteridine<br>1	E2<br>2,4-Diamino-6,7-diisopropyl-pteridine<br>2	E3<br>2,4-Diamino-6,7-diisopropyl-pteridine<br>3	E4<br>2,4-Diamino-6,7-diisopropyl-pteridine<br>4	E5<br>Sulfadiazine<br>1	E6<br>Sulfadiazine<br>2	E7<br>Sulfadiazine<br>3	E8<br>Sulfadiazine<br>4	E9<br>Benzethonium chloride<br>1	E10<br>Benzethonium chloride<br>2	E11<br>Benzethonium chloride<br>3	E12<br>Benzethonium chloride<br>4
F1<br>Tobramycin<br>1	F2<br>Tobramycin<br>2	F3<br>Tobramycin<br>3	F4<br>Tobramycin<br>4	F5<br>Sulfathiazole<br>1	F6<br>Sulfathiazole<br>2	F7<br>Sulfathiazole<br>3	F8<br>Sulfathiazole<br>4	F9<br>5-Fluoroorotic acid<br>1	F10<br>5-Fluoroorotic acid<br>2	F11<br>5-Fluoroorotic acid<br>3	F12<br>5-Fluoroorotic acid<br>4
G1<br>Spectinomycin<br>1	G2<br>Spectinomycin<br>2	G3<br>Spectinomycin<br>3	G4<br>Spectinomycin<br>4	G5<br>Sulfa-methoxazole<br>1	G6<br>Sulfa-methoxazole<br>2	G7<br>Sulfa-methoxazole<br>3	G8<br>Sulfa-methoxazole<br>4	G9<br>L-Aspartic-β-hydroxamate<br>1	G10<br>L-Aspartic-β-hydroxamate<br>2	G11<br>L-Aspartic-β-hydroxamate<br>3	G12<br>L-Aspartic-β-hydroxamate<br>4
H1<br>Spiramycin<br>1	H2<br>Spiramycin<br>2	H3<br>Spiramycin<br>3	H4<br>Spiramycin<br>4	H5<br>Rifampicin<br>1	H6<br>Rifampicin<br>2	H7<br>Rifampicin<br>3	H8<br>Rifampicin<br>4	H9<br>Dodecyltrimethyl ammonium bromide<br>1	H10<br>Dodecyltrimethyl ammonium bromide<br>2	H11<br>Dodecyltrimethyl ammonium bromide<br>3	H12<br>Dodecyltrimethyl ammonium bromide<br>4

PM11 (Chemical Sensitivity Bacteria)

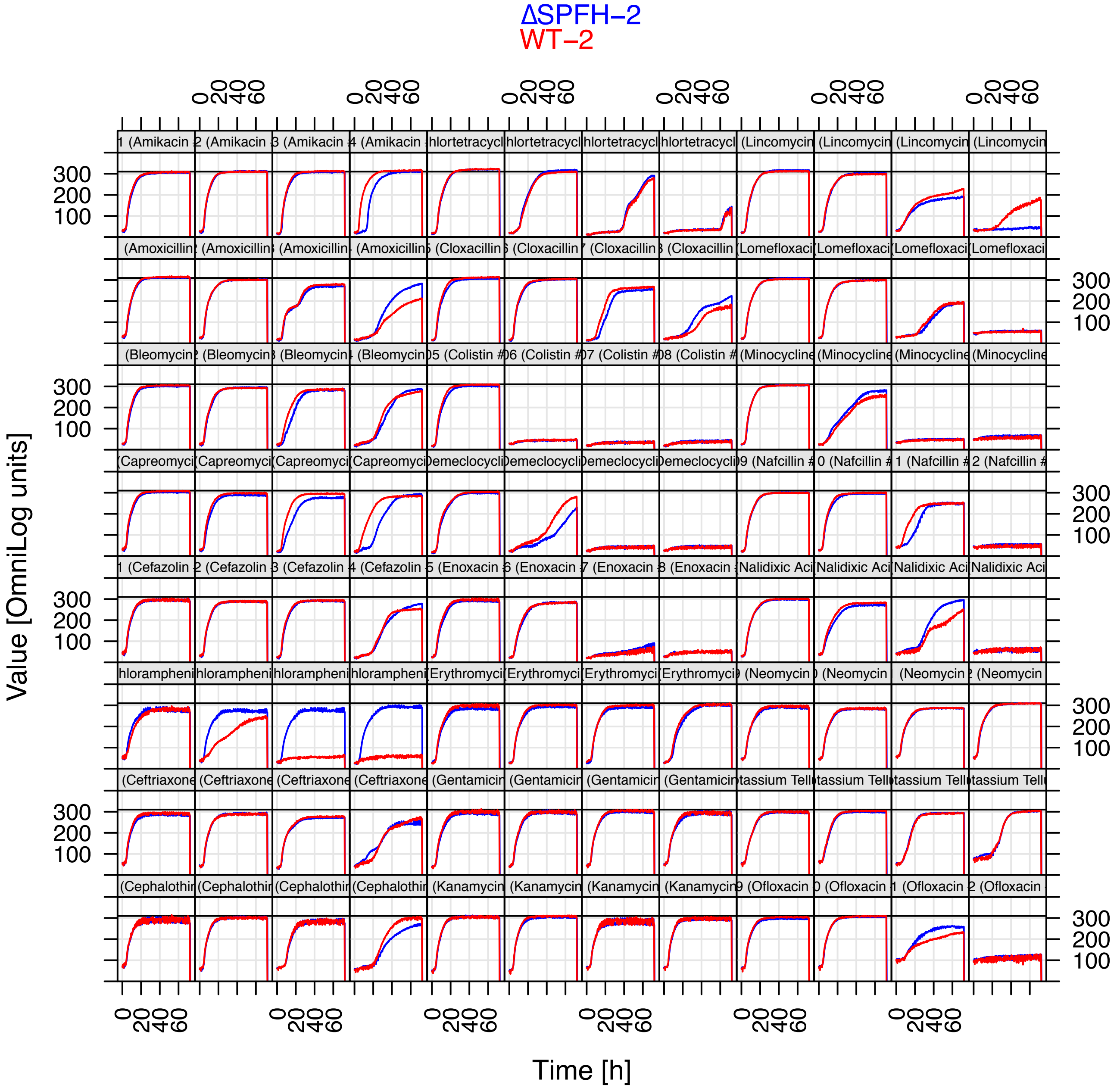

PM11 (Chemicals)

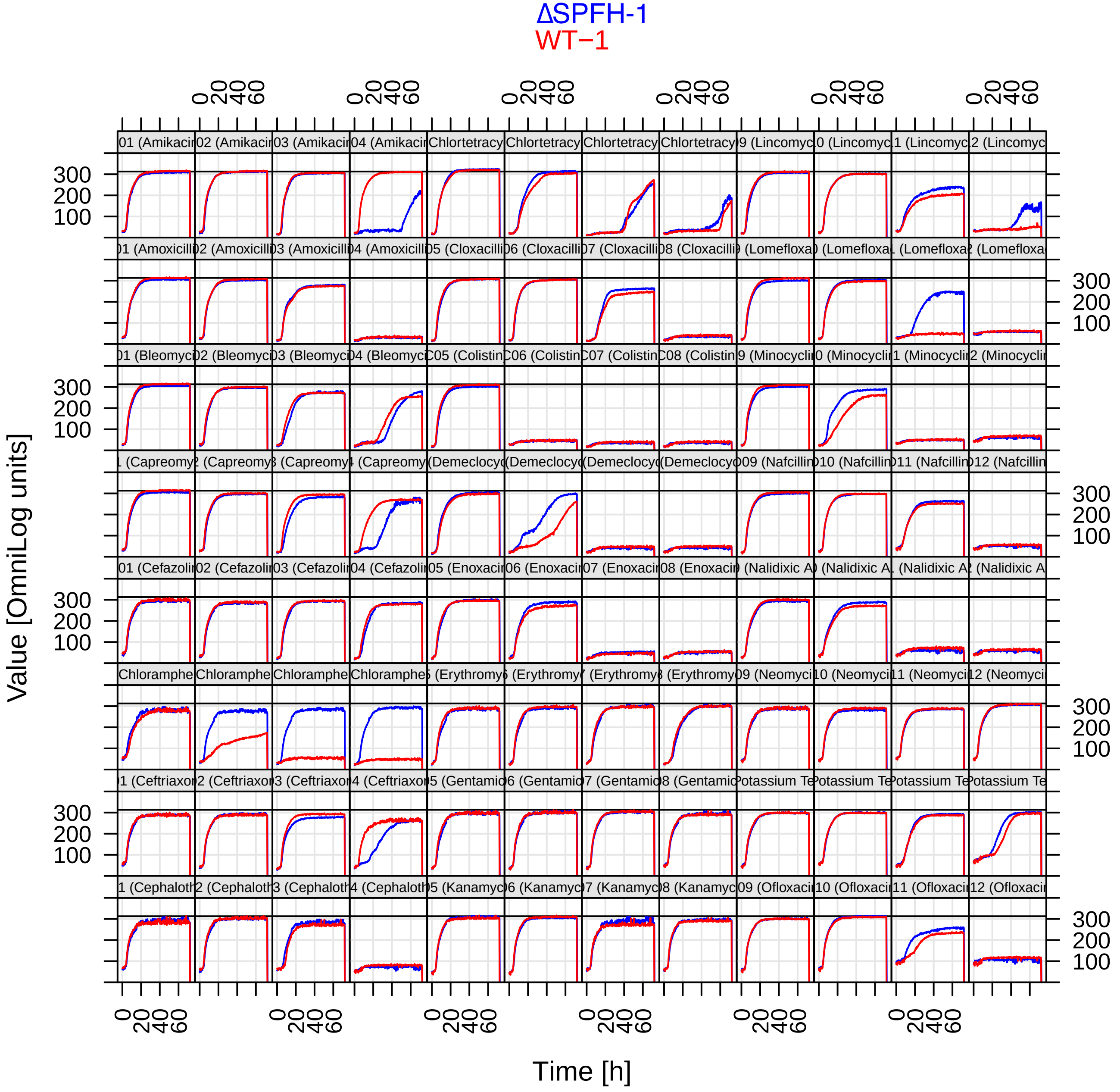

PM12 (Chemical Sensitivity Bacteria)

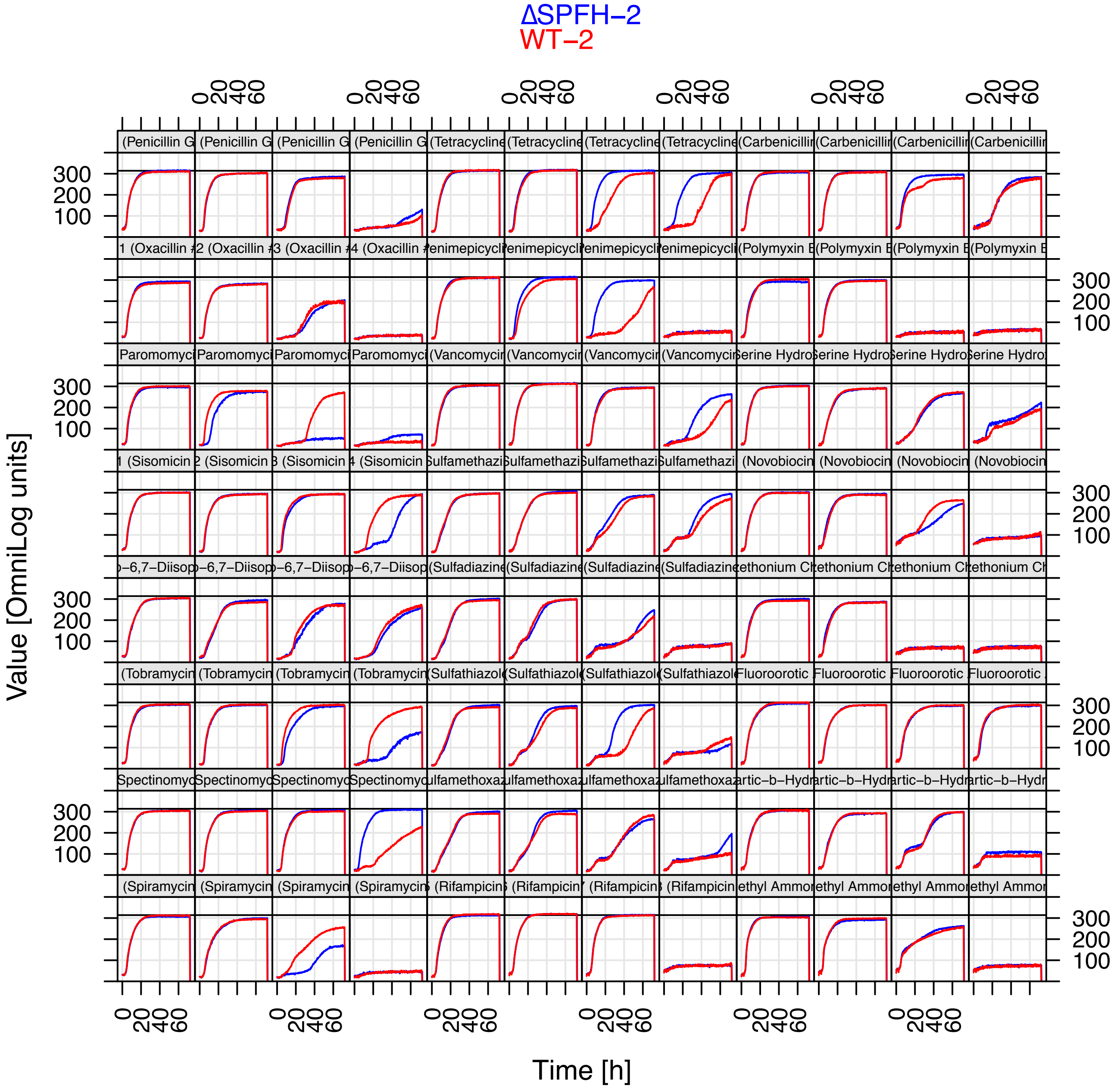

PM12 (Chemicals)

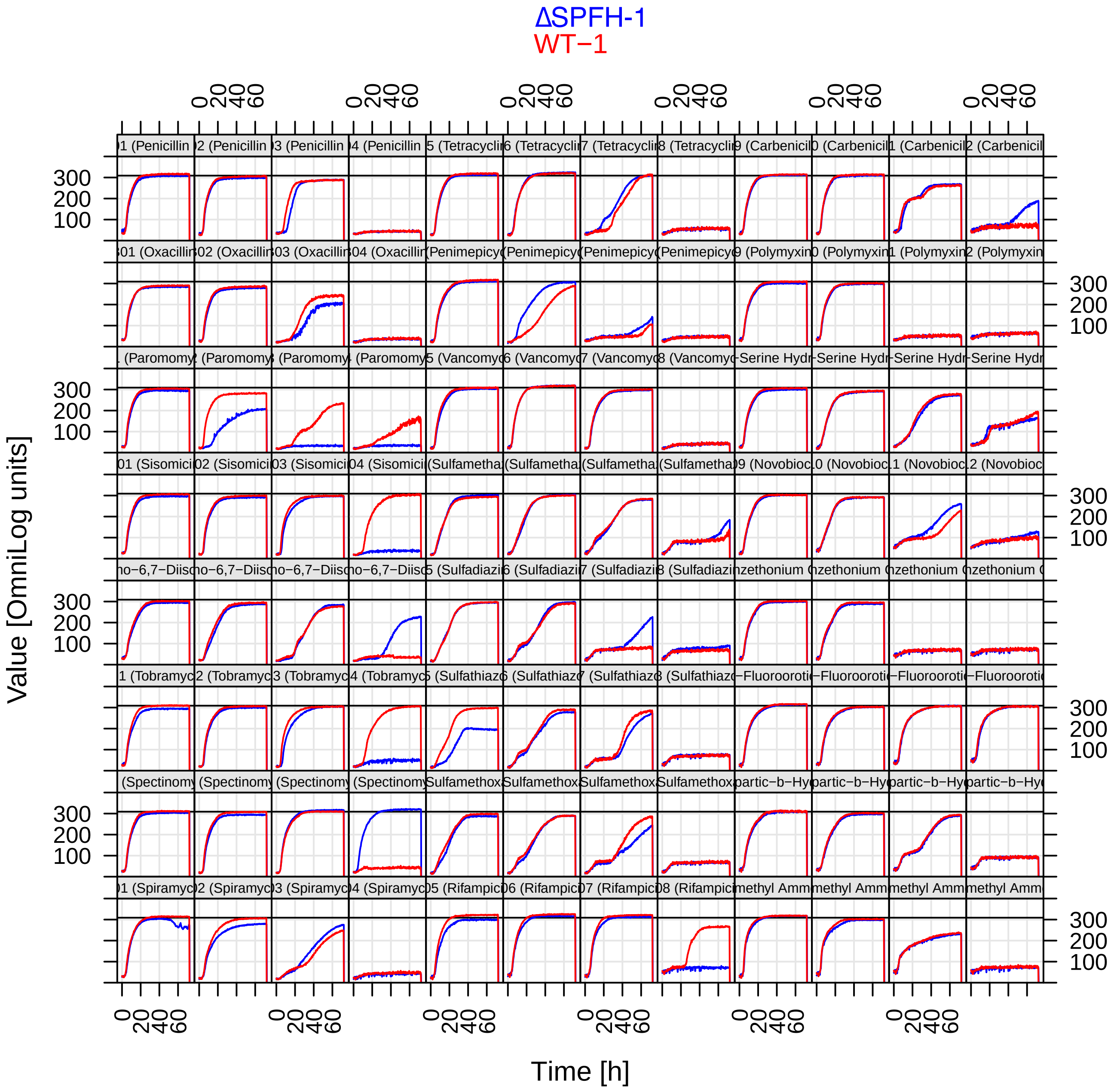

PM13B MicroPlate™

A1<br>Ampicillin<br>1	A2<br>Ampicillin<br>2	A3<br>Ampicillin<br>3	A4<br>Ampicillin<br>4	A5<br>Dequalinium chloride<br>1	A6<br>Dequalinium chloride<br>2	A7<br>Dequalinium chloride<br>3	A8<br>Dequalinium chloride<br>4	A9<br>Nickel chloride<br>1	A10<br>Nickel chloride<br>2	A11<br>Nickel chloride<br>3	A12<br>Nickel chloride<br>4
B1<br>Azlocillin<br>1	B2<br>Azlocillin<br>2	B3<br>Azlocillin<br>3	B4<br>Azlocillin<br>4	B5<br>2, 2'-Dipyridyl<br>1	B6<br>2, 2'-Dipyridyl<br>2	B7<br>2, 2'-Dipyridyl<br>3	B8<br>2, 2'-Dipyridyl<br>4	B9<br>Oxolinic acid<br>1	B10<br>Oxolinic acid<br>2	B11<br>Oxolinic acid<br>3	B12<br>Oxolinic acid<br>4
C1<br>6-Mercapto-<br>purine<br>1	C2<br>6-Mercapto-<br>purine<br>2	C3<br>6-Mercapto-<br>purine<br>3	C4<br>6-Mercapto-<br>purine<br>4	C5<br>Doxycycline<br>1	C6<br>Doxycycline<br>2	C7<br>Doxycycline<br>3	C8<br>Doxycycline<br>4	C9<br>Potassium chromate<br>1	C10<br>Potassium chromate<br>2	C11<br>Potassium chromate<br>3	C12<br>Potassium chromate<br>4
D1<br>Cefuroxime<br>1	D2<br>Cefuroxime<br>2	D3<br>Cefuroxime<br>3	D4<br>Cefuroxime<br>4	D5<br>5-Fluorouracil<br>1	D6<br>5-Fluorouracil<br>2	D7<br>5-Fluorouracil<br>3	D8<br>5-Fluorouracil<br>4	D9<br>Rolitetracycline<br>1	D10<br>Rolitetracycline<br>2	D11<br>Rolitetracycline<br>3	D12<br>Rolitetracycline<br>4
E1<br>Cytosine-1-beta-<br>D-arabino-<br>furanoside<br>1	E2<br>Cytosine-1-beta-<br>D-arabino-<br>furanoside<br>2	E3<br>Cytosine-1-beta-<br>D-arabino-<br>furanoside<br>3	E4<br>Cytosine-1-beta-<br>D-arabino-<br>furanoside<br>4	E5<br>Geneticin (G418)<br>1	E6<br>Geneticin (G418)<br>2	E7<br>Geneticin (G418)<br>3	E8<br>Geneticin (G418)<br>4	E9<br>Ruthenium red<br>1	E10<br>Ruthenium red<br>2	E11<br>Ruthenium red<br>3	E12<br>Ruthenium red<br>4
F1<br>Cesium chloride<br>1	F2<br>Cesium chloride<br>2	F3<br>Cesium chloride<br>3	F4<br>Cesium chloride<br>4	F5<br>Glycine<br>1	F6<br>Glycine<br>2	F7<br>Glycine<br>3	F8<br>Glycine<br>4	F9<br>Thallium (I) acetate<br>1	F10<br>Thallium (I) acetate<br>2	F11<br>Thallium (I) acetate<br>3	F12<br>Thallium (I) acetate<br>4
G1<br>Cobalt chloride<br>1	G2<br>Cobalt chloride<br>2	G3<br>Cobalt chloride<br>3	G4<br>Cobalt chloride<br>4	G5<br>Manganese chloride<br>1	G6<br>Manganese chloride<br>2	G7<br>Manganese chloride<br>3	G8<br>Manganese chloride<br>4	G9<br>Trifluoperazine<br>1	G10<br>Trifluoperazine<br>2	G11<br>Trifluoperazine<br>3	G12<br>Trifluoperazine<br>4
H1<br>Cupric chloride<br>1	H2<br>Cupric chloride<br>2	H3<br>Cupric chloride<br>3	H4<br>Cupric chloride<br>4	H5<br>Moxalactam<br>1	H6<br>Moxalactam<br>2	H7<br>Moxalactam<br>3	H8<br>Moxalactam<br>4	H9<br>Tylosin<br>1	H10<br>Tylosin<br>2	H11<br>Tylosin<br>3	H12<br>Tylosin<br>4

PM14A MicroPlate™

A1<br>Acriflavine<br>1	A2<br>Acriflavine<br>2	A3<br>Acriflavine<br>3	A4<br>Acriflavine<br>4	A5<br>Furaltadone<br>1	A6<br>Furaltadone<br>2	A7<br>Furaltadone<br>3	A8<br>Furaltadone<br>4	A9<br>Sanguinarine<br>1	A10<br>Sanguinarine<br>2	A11<br>Sanguinarine<br>3	A12<br>Sanguinarine<br>4
B1<br>9-Aminoacridine<br>1	B2<br>9-Aminoacridine<br>2	B3<br>9-Aminoacridine<br>3	B4<br>9-Aminoacridine<br>4	B5<br>Fusaric acid<br>1	B6<br>Fusaric acid<br>2	B7<br>Fusaric acid<br>3	B8<br>Fusaric acid<br>4	B9<br>Sodium arsenate<br>1	B10<br>Sodium arsenate<br>2	B11<br>Sodium arsenate<br>3	B12<br>Sodium arsenate<br>4
C1<br>Boric Acid<br>1	C2<br>Boric Acid<br>2	C3<br>Boric Acid<br>3	C4<br>Boric Acid<br>4	C5<br>1-Hydroxy-<br>pyridine -2-<br>thione<br>1	C6<br>1-Hydroxy-<br>pyridine -2-<br>thione<br>2	C7<br>1-Hydroxy-<br>pyridine -2-<br>thione<br>3	C8<br>1-Hydroxy-<br>pyridine -2-<br>thione<br>4	C9<br>Sodium cyanate<br>1	C10<br>Sodium cyanate<br>2	C11<br>Sodium cyanate<br>3	C12<br>Sodium cyanate<br>4
D1<br>Cadmium chloride<br>1	D2<br>Cadmium chloride<br>2	D3<br>Cadmium chloride<br>3	D4<br>Cadmium chloride<br>4	D5<br>Iodoacetate<br>1	D6<br>Iodoacetate<br>2	D7<br>Iodoacetate<br>3	D8<br>Iodoacetate<br>4	D9<br>Sodium dichromate<br>1	D10<br>Sodium dichromate<br>2	D11<br>Sodium dichromate<br>3	D12<br>Sodium dichromate<br>4
E1<br>Cefoxitin<br>1	E2<br>Cefoxitin<br>2	E3<br>Cefoxitin<br>3	E4<br>Cefoxitin<br>4	E5<br>Nitrofurantoin<br>1	E6<br>Nitrofurantoin<br>2	E7<br>Nitrofurantoin<br>3	E8<br>Nitrofurantoin<br>4	E9<br>Sodium metaborate<br>1	E10<br>Sodium metaborate<br>2	E11<br>Sodium metaborate<br>3	E12<br>Sodium metaborate<br>4
F1<br>Chloramphenicol<br>1	F2<br>Chloramphenicol<br>2	F3<br>Chloramphenicol<br>3	F4<br>Chloramphenicol<br>4	F5<br>Piperacillin<br>1	F6<br>Piperacillin<br>2	F7<br>Piperacillin<br>3	F8<br>Piperacillin<br>4	F9<br>Sodium metavanadate<br>1	F10<br>Sodium metavanadate<br>2	F11<br>Sodium metavanadate<br>3	F12<br>Sodium metavanadate<br>4
G1<br>Chelerythrine<br>1	G2<br>Chelerythrine<br>2	G3<br>Chelerythrine<br>3	G4<br>Chelerythrine<br>4	G5<br>Carbenicillin<br>1	G6<br>Carbenicillin<br>2	G7<br>Carbenicillin<br>3	G8<br>Carbenicillin<br>4	G9<br>Sodium nitrite<br>1	G10<br>Sodium nitrite<br>2	G11<br>Sodium nitrite<br>3	G12<br>Sodium nitrite<br>4
H1<br>EGTA<br>1	H2<br>EGTA<br>2	H3<br>EGTA<br>3	H4<br>EGTA<br>4	H5<br>Promethazine<br>1	H6<br>Promethazine<br>2	H7<br>Promethazine<br>3	H8<br>Promethazine<br>4	H9<br>Sodium orthovanadate<br>1	H10<br>Sodium orthovanadate<br>2	H11<br>Sodium orthovanadate<br>3	H12<br>Sodium orthovanadate<br>4

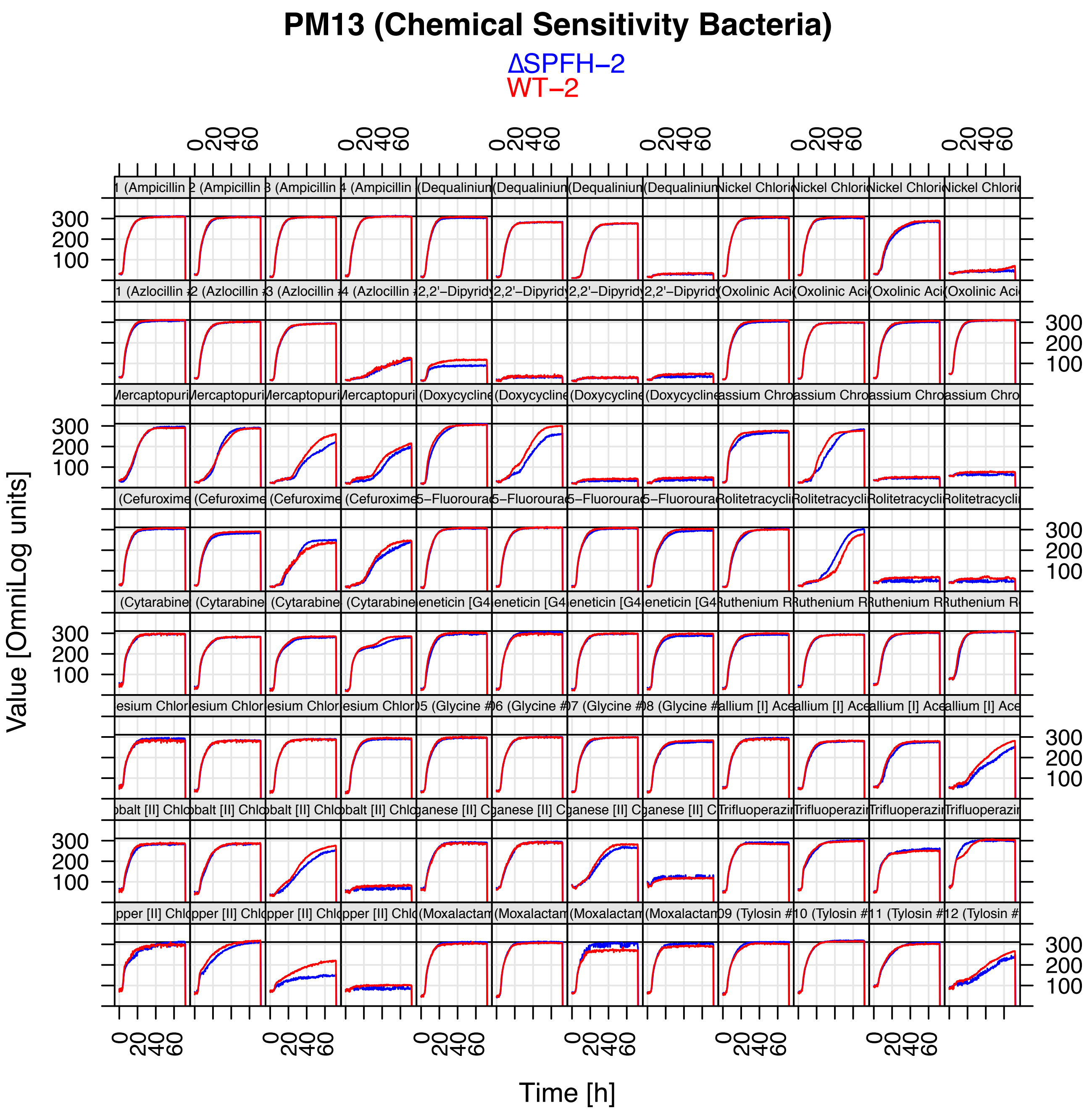
PM13 (Chemical Sensitivity Bacteria)
ΔSPFH−2
WT−2
Value [OmniLog units]
Time [h]
1 (Ampicillin
2 (Ampicillin
3 (Ampicillin
4 (Ampicillin
(Dequaliniun
Nickel Chloric
1 (Azlocillin
2 (Azlocillin
3 (Azlocillin
4 (Azlocillin
2,2'−Dipyrid
Oxolinic Aci
Mercaptopuri
(Doxycycline
assium Chro
(Cefuroxime
5−Fluoroura
Rolitetracycli
(Cytarabine
eneticin [G4
Ruthenium R
esium Chlor
5 (Glycine #
6 (Glycine #
7 (Glycine #
8 (Glycine #
allium [I] Ace
balt [II] Chlo
ganese [II] C
Trifluoperazir
pper [II] Chlo
(Moxalactam
09 (Tylosin #
10 (Tylosin #
11 (Tylosin #
12 (Tylosin #

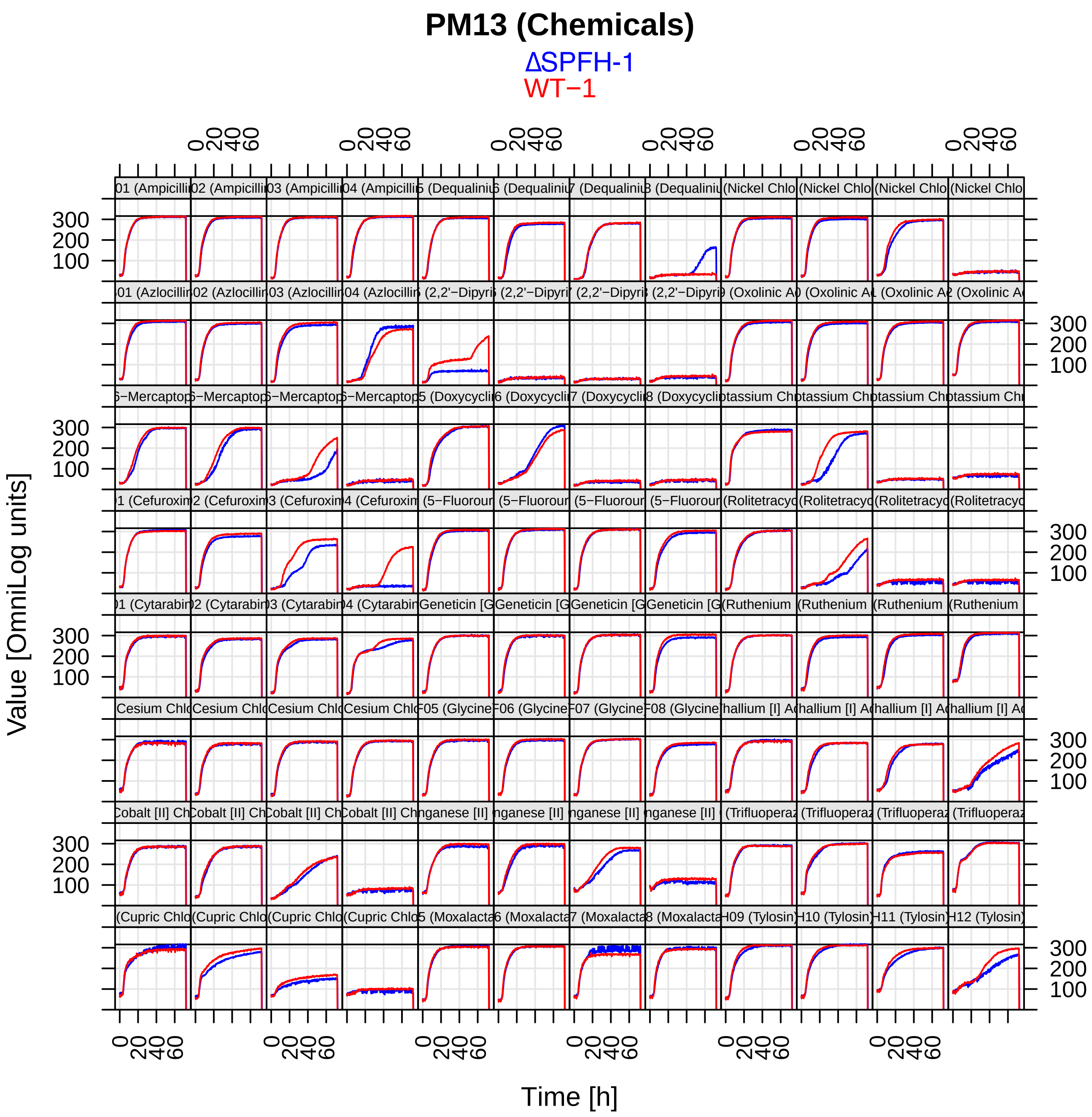
PM13 (Chemicals)
ΔSPFH-1
WT−1
Value [OmniLog units]
Time [h]
01 (Ampicilli
02 (Ampicilli
03 (Ampicilli
04 (Ampicilli
5 (Dequaliniu
6 (Dequaliniu
7 (Dequaliniu
8 (Dequaliniu
(Nickel Chlo
01 (Azlocillin
02 (Azlocillin
03 (Azlocillin
04 (Azlocillin
(2,2'−Dipyri
(Oxolinic A
5 (Doxycycli
6 (Doxycycli
7 (Doxycycli
8 (Doxycycli
(Cefuroxim
(5−Fluorour
(Rolitetracyc
(Cytarabin
Geneticin [G
(Ruthenium
Cesium Chlo
F05 (Glycine
F06 (Glycine
F07 (Glycine
F08 (Glycine
hallium [I] Ac
Cobalt [II] Ch
nganese [II]
(Trifluoperaz
(Cupric Chlo
5 (Moxalacta
6 (Moxalacta
7 (Moxalacta
8 (Moxalacta
H09 (Tylosin)
H10 (Tylosin)
H11 (Tylosin)
H12 (Tylosin)

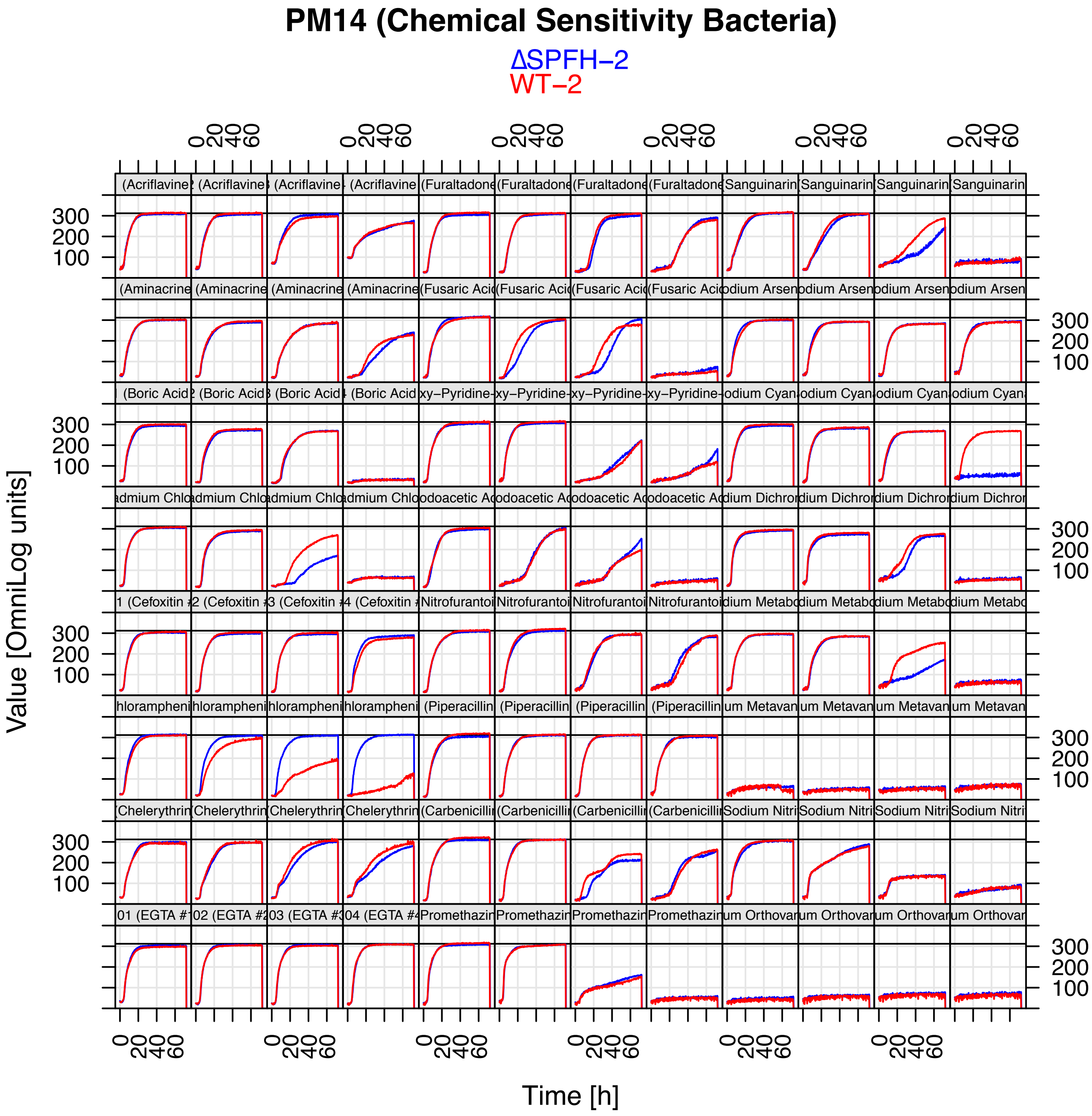
PM14 (Chemical Sensitivity Bacteria)
ΔSPFH−2
WT−2
Value [OmniLog units]
Time [h]
300
200
100
0
20
40
60

PM14 (Chemicals)

ΔSPFH-1
WT−1

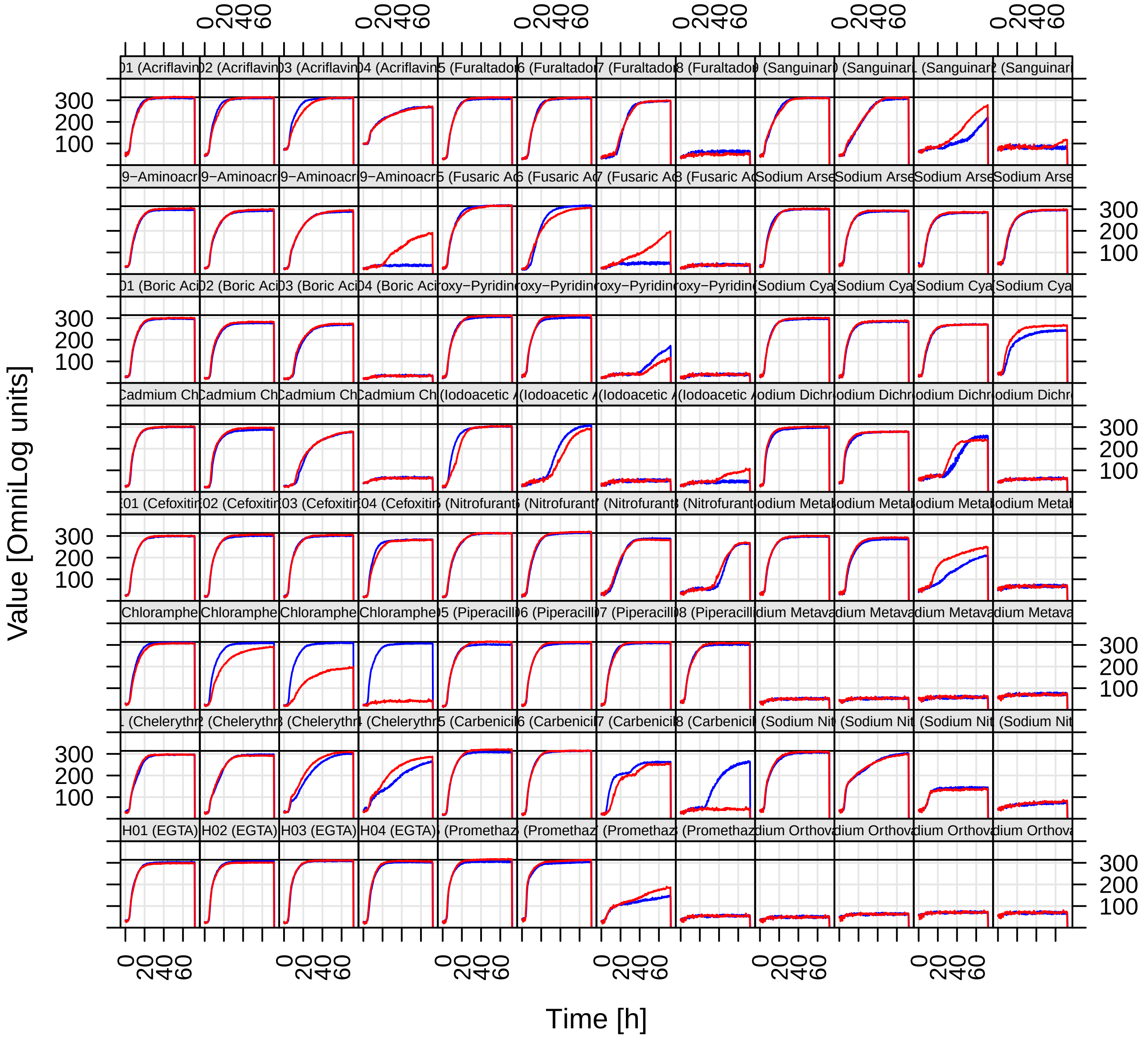

PM15B MicroPlate™

A1<br>Procaine<br>1	A2<br>Procaine<br>2	A3<br>Procaine<br>3	A4<br>Procaine<br>4	A5<br>Guanidine hydrochloride<br>1	A6<br>Guanidine hydrochloride<br>2	A7<br>Guanidine hydrochloride<br>3	A8<br>Guanidine hydrochloride<br>4	A9<br>Cefmetazole<br>1	A10<br>Cefmetazole<br>2	A11<br>Cefmetazole<br>3	A12<br>Cefmetazole<br>4
B1<br>D-Cycloserine<br>1	B2<br>D-Cycloserine<br>2	B3<br>D-Cycloserine<br>3	B4<br>D-Cycloserine<br>4	B5<br>EDTA<br>1	B6<br>EDTA<br>2	B7<br>EDTA<br>3	B8<br>EDTA<br>4	B9<br>5,7-Dichloro- 8-hydroxy-quinaldine<br>1	B10<br>5,7-Dichloro- 8-hydroxy-quinaldine<br>2	B11<br>5,7-Dichloro- 8-hydroxy-quinaldine<br>3	B12<br>5,7-Dichloro- 8-hydroxy-quinaldine<br>4
C1<br>5,7-Dichloro-8-hydroxyquinoline<br>1	C2<br>5,7-Dichloro-8-hydroxyquinoline<br>2	C3<br>5,7-Dichloro-8-hydroxyquinoline<br>3	C4<br>5,7-Dichloro-8-hydroxyquinoline<br>4	C5<br>Fusidic acid<br>1	C6<br>Fusidic acid<br>2	C7<br>Fusidic acid<br>3	C8<br>Fusidic acid<br>4	C9<br>1,10-Phenanthroline<br>1	C10<br>1,10-Phenanthroline<br>2	C11<br>1,10-Phenanthroline<br>3	C12<br>1,10-Phenanthroline<br>4
D1<br>Phleomycin<br>1	D2<br>Phleomycin<br>2	D3<br>Phleomycin<br>3	D4<br>Phleomycin<br>4	D5<br>Domiphen bromide<br>1	D6<br>Domiphen bromide<br>2	D7<br>Domiphen bromide<br>3	D8<br>Domiphen bromide<br>4	D9<br>Nordihydroguaiaretic acid<br>1	D10<br>Nordihydroguaiaretic acid<br>2	D11<br>Nordihydroguaiaretic acid<br>3	D12<br>Nordihydroguaiaretic acid<br>4
E1<br>Alexidine<br>1	E2<br>Alexidine<br>2	E3<br>Alexidine<br>3	E4<br>Alexidine<br>4	E5<br>5-Nitro-2-furaldehyde semicarbazone<br>1	E6<br>5-Nitro-2-furaldehyde semicarbazone<br>2	E7<br>5-Nitro-2-furaldehyde semicarbazone<br>3	E8<br>5-Nitro-2-furaldehyde semicarbazone<br>4	E9<br>Methyl viologen<br>1	E10<br>Methyl viologen<br>2	E11<br>Methyl viologen<br>3	E12<br>Methyl viologen<br>4
F1<br>3, 4-Dimethoxy-benzyl alcohol<br>1	F2<br>3, 4-Dimethoxy-benzyl alcohol<br>2	F3<br>3, 4-Dimethoxy-benzyl alcohol<br>3	F4<br>3, 4-Dimethoxy-benzyl alcohol<br>4	F5<br>Oleandomycin<br>1	F6<br>Oleandomycin<br>2	F7<br>Oleandomycin<br>3	F8<br>Oleandomycin<br>4	F9<br>Puromycin<br>1	F10<br>Puromycin<br>2	F11<br>Puromycin<br>3	F12<br>Puromycin<br>4
G1<br>CCCP<br>1	G2<br>CCCP<br>2	G3<br>CCCP<br>3	G4<br>CCCP<br>4	G5<br>Sodium azide<br>1	G6<br>Sodium azide<br>2	G7<br>Sodium azide<br>3	G8<br>Sodium azide<br>4	G9<br>Menadione<br>1	G10<br>Menadione<br>2	G11<br>Menadione<br>3	G12<br>Menadione<br>4
H1<br>2-Nitroimidazole<br>1	H2<br>2-Nitroimidazole<br>2	H3<br>2-Nitroimidazole<br>3	H4<br>2-Nitroimidazole<br>4	H5<br>Hydroxyurea<br>1	H6<br>Hydroxyurea<br>2	H7<br>Hydroxyurea<br>3	H8<br>Hydroxyurea<br>4	H9<br>Zinc chloride<br>1	H10<br>Zinc chloride<br>2	H11<br>Zinc chloride<br>3	H12<br>Zinc chloride<br>4

PM16A MicroPlate™

A1<br>Cefotaxime<br>1	A2<br>Cefotaxime<br>2	A3<br>Cefotaxime<br>3	A4<br>Cefotaxime<br>4	A5<br>Phosphomycin<br>1	A6<br>Phosphomycin<br>2	A7<br>Phosphomycin<br>3	A8<br>Phosphomycin<br>4	A9<br>5-Chloro-7-iodo-8-hydroxy-quinoline<br>1	A10<br>5-Chloro-7-iodo-8-hydroxy-quinoline<br>2	A11<br>5-Chloro-7-iodo-8-hydroxy-quinoline<br>3	A12<br>5-Chloro-7-iodo-8-hydroxy-quinoline<br>4
B1<br>Norfloxacin<br>1	B2<br>Norfloxacin<br>2	B3<br>Norfloxacin<br>3	B4<br>Norfloxacin<br>4	B5<br>Sulfanilamide<br>1	B6<br>Sulfanilamide<br>2	B7<br>Sulfanilamide<br>3	B8<br>Sulfanilamide<br>4	B9<br>Trimethoprim<br>1	B10<br>Trimethoprim<br>2	B11<br>Trimethoprim<br>3	B12<br>Trimethoprim<br>4
C1<br>Dichlofluanid<br>1	C2<br>Dichlofluanid<br>2	C3<br>Dichlofluanid<br>3	C4<br>Dichlofluanid<br>4	C5<br>Protamine sulfate<br>1	C6<br>Protamine sulfate<br>2	C7<br>Protamine sulfate<br>3	C8<br>Protamine sulfate<br>4	C9<br>Cetylpyridinium chloride<br>1	C10<br>Cetylpyridinium chloride<br>2	C11<br>Cetylpyridinium chloride<br>3	C12<br>Cetylpyridinium chloride<br>4
D1<br>1-Chloro -2,4-dinitrobenzene<br>1	D2<br>1-Chloro -2,4-dinitrobenzene<br>2	D3<br>1-Chloro -2,4-dinitrobenzene<br>3	D4<br>1-Chloro -2,4-dinitrobenzene<br>4	D5<br>Diamide<br>1	D6<br>Diamide<br>2	D7<br>Diamide<br>3	D8<br>Diamide<br>4	D9<br>Cinoxacin<br>1	D10<br>Cinoxacin<br>2	D11<br>Cinoxacin<br>3	D12<br>Cinoxacin<br>4
E1<br>Streptomycin<br>1	E2<br>Streptomycin<br>2	E3<br>Streptomycin<br>3	E4<br>Streptomycin<br>4	E5<br>5-Azacytidine<br>1	E6<br>5-Azacytidine<br>2	E7<br>5-Azacytidine<br>3	E8<br>5-Azacytidine<br>4	E9<br>Rifamycin SV<br>1	E10<br>Rifamycin SV<br>2	E11<br>Rifamycin SV<br>3	E12<br>Rifamycin SV<br>4
F1<br>Potassium tellurite<br>1	F2<br>Potassium tellurite<br>2	F3<br>Potassium tellurite<br>3	F4<br>Potassium tellurite<br>4	F5<br>Sodium selenite<br>1	F6<br>Sodium selenite<br>2	F7<br>Sodium selenite<br>3	F8<br>Sodium selenite<br>4	F9<br>Aluminum sulfate<br>1	F10<br>Aluminum sulfate<br>2	F11<br>Aluminum sulfate<br>3	F12<br>Aluminum sulfate<br>4
G1<br>Chromium chloride<br>1	G2<br>Chromium chloride<br>2	G3<br>Chromium chloride<br>3	G4<br>Chromium chloride<br>4	G5<br>Ferric chloride<br>1	G6<br>Ferric chloride<br>2	G7<br>Ferric chloride<br>3	G8<br>Ferric chloride<br>4	G9<br>L-Glutamic-g-hydroxamate<br>1	G10<br>L-Glutamic-g-hydroxamate<br>2	G11<br>L-Glutamic-g-hydroxamate<br>3	G12<br>L-Glutamic-g-hydroxamate<br>4
H1<br>Glycine hydroxamate<br>1	H2<br>Glycine hydroxamate<br>2	H3<br>Glycine hydroxamate<br>3	H4<br>Glycine hydroxamate<br>4	H5<br>Chloroxylenol<br>1	H6<br>Chloroxylenol<br>2	H7<br>Chloroxylenol<br>3	H8<br>Chloroxylenol<br>4	H9<br>Sorbic acid<br>1	H10<br>Sorbic acid<br>2	H11<br>Sorbic acid<br>3	H12<br>Sorbic acid<br>4

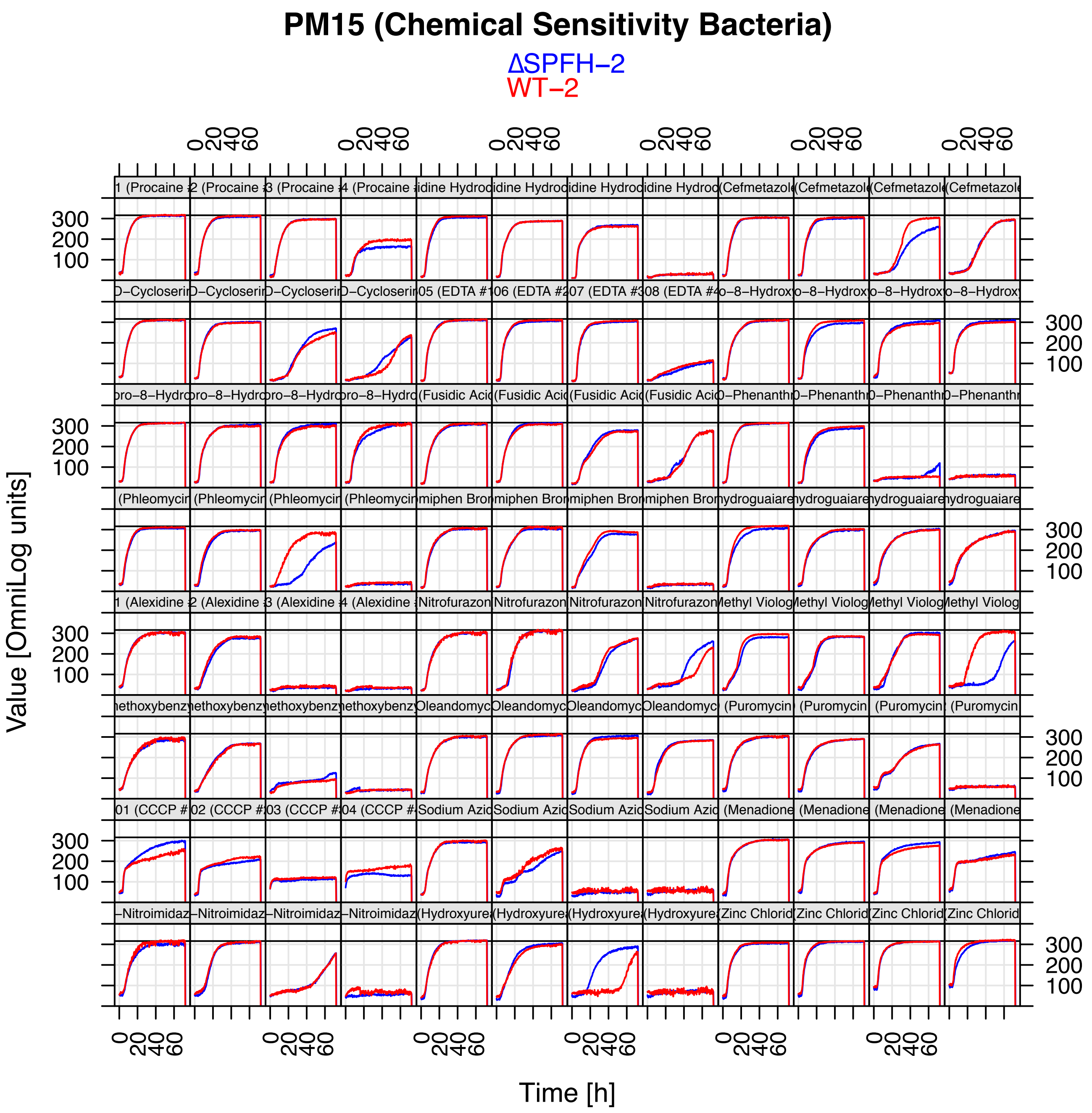
PM15 (Chemical Sensitivity Bacteria)
ΔSPFH−2
WT−2
Value [OmniLog units]
Time [h]

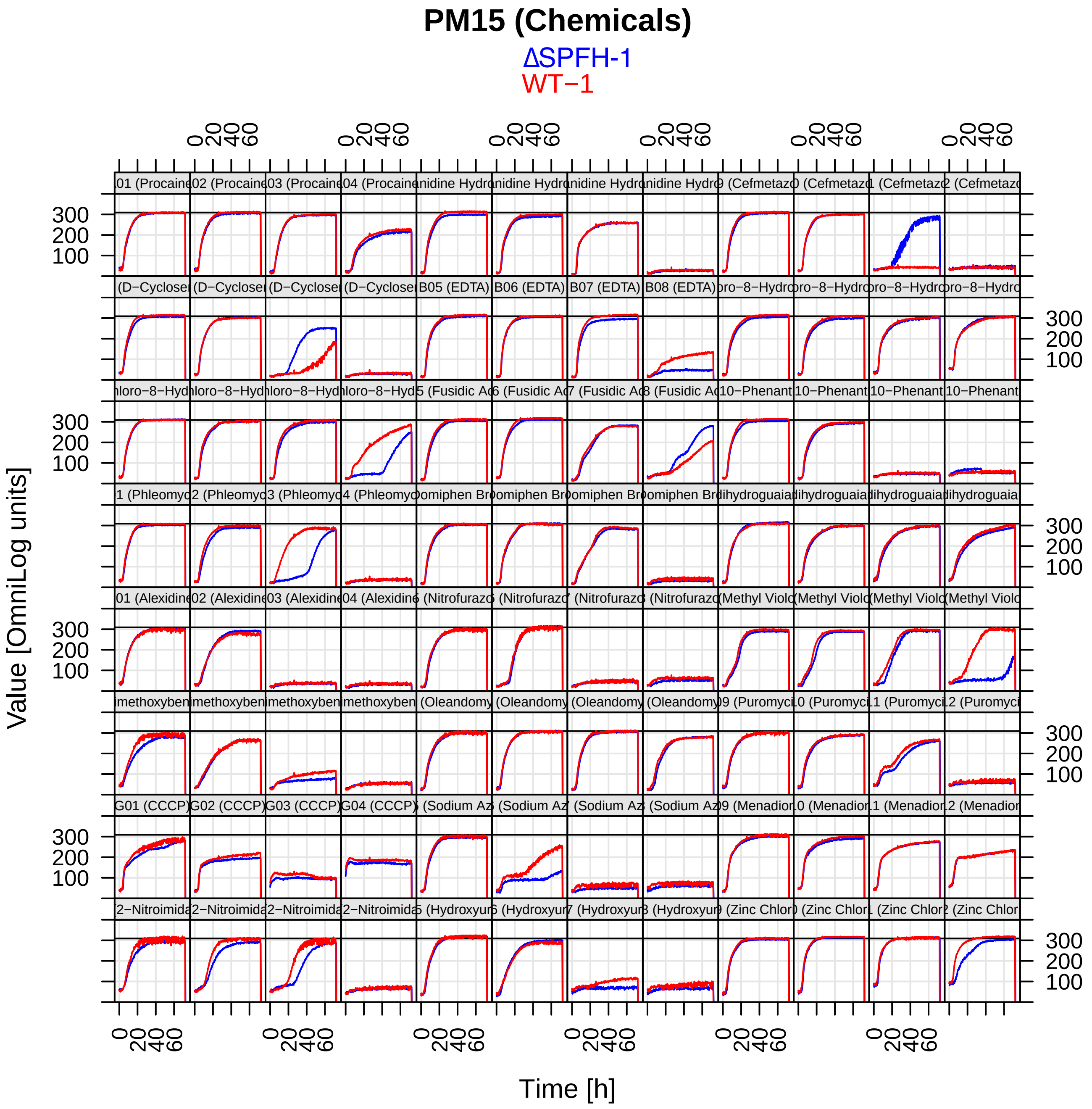
PM15 (Chemicals)
ΔSPFH-1
WT−1
Value [OmniLog units]
Time [h]

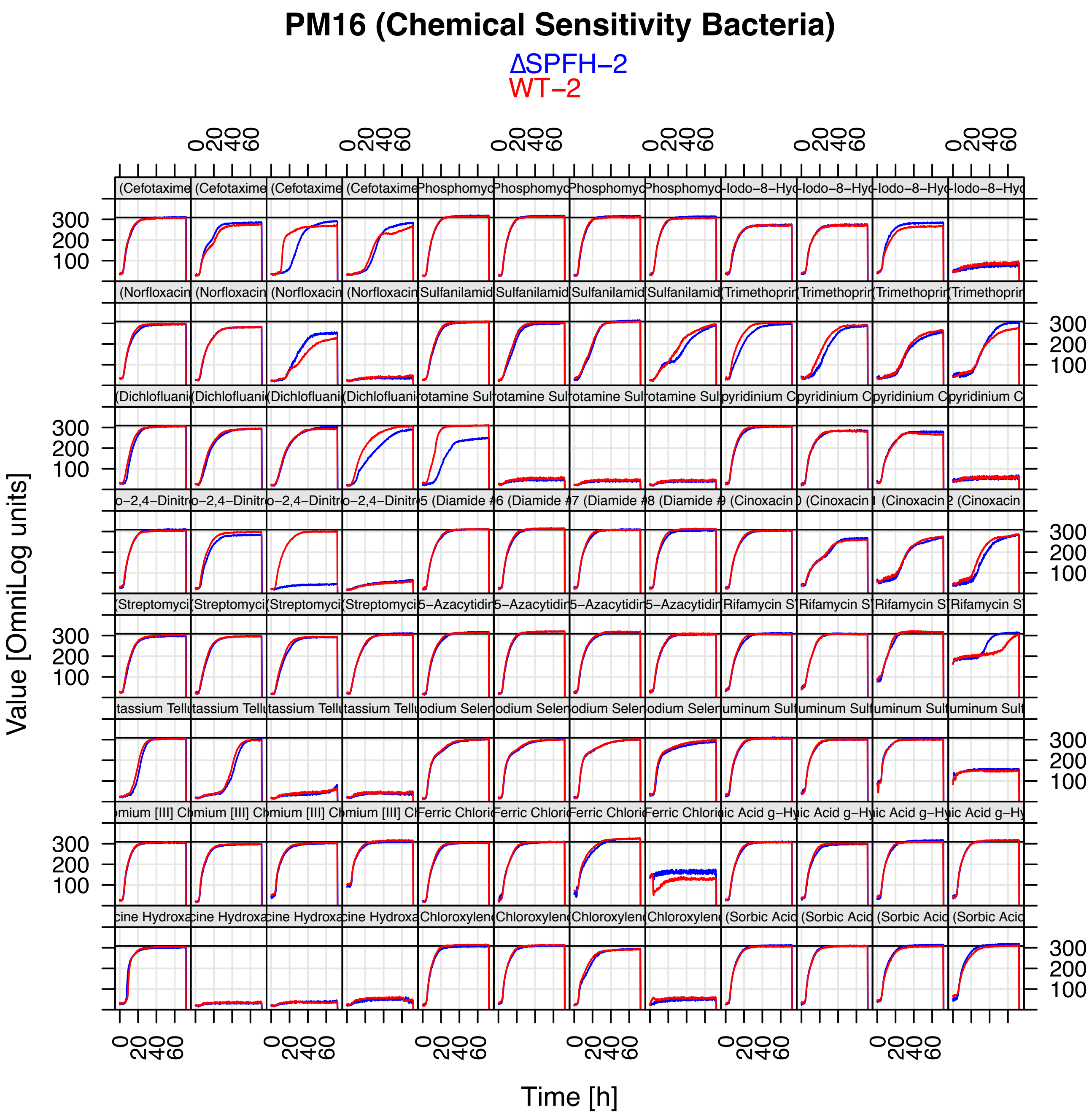
PM16 (Chemical Sensitivity Bacteria)
ΔSPFH−2
WT−2
Value [OmniLog units]
Time [h]

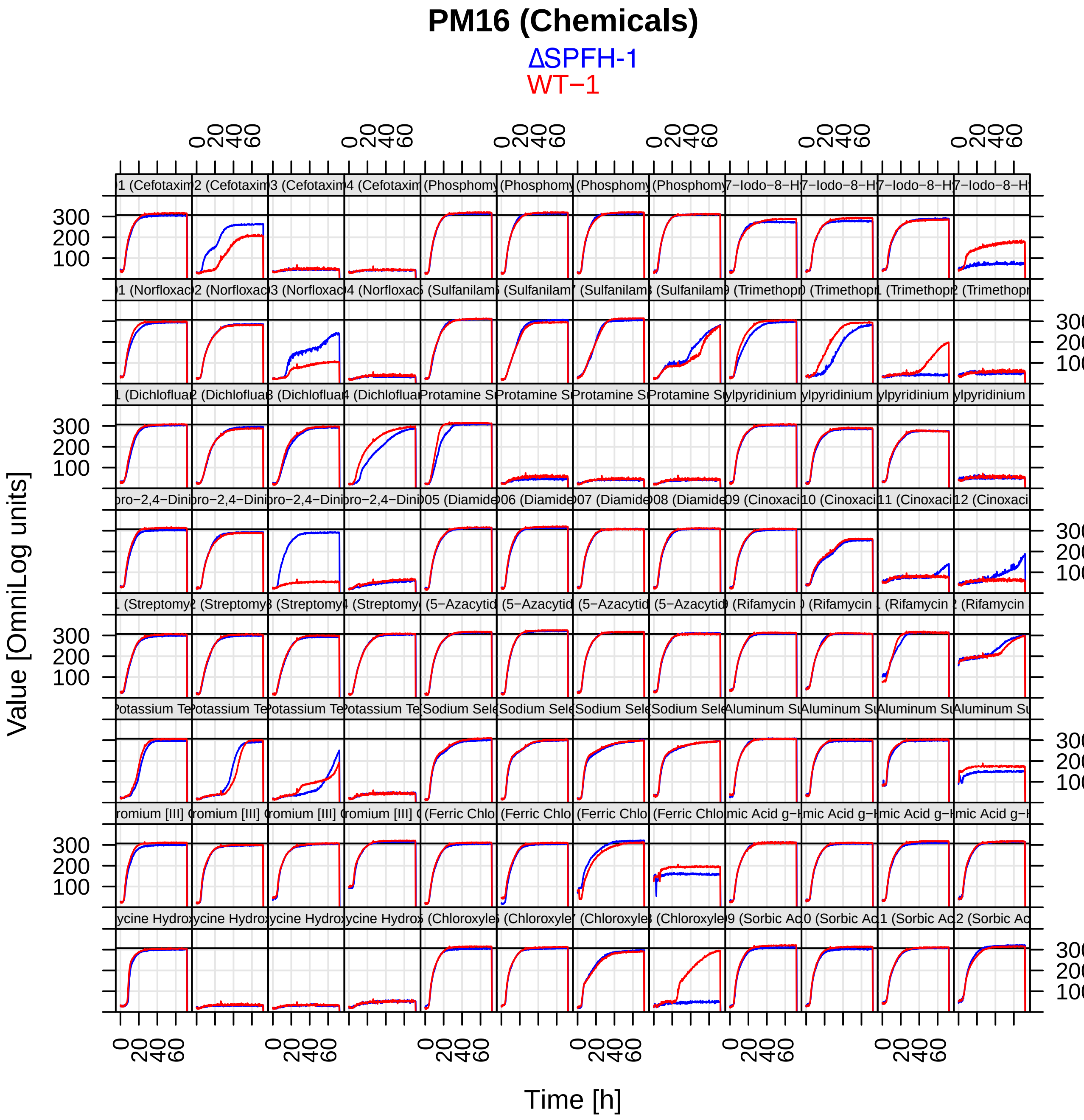

PM16 (Chemicals)
ΔSPFH-1
WT−1
Value [OmniLog units]
Time [h]

PM17A MicroPlate™

A1<br>D-Serine<br>1	A2<br>D-Serine<br>2	A3<br>D-Serine<br>3	A4<br>D-Serine<br>4	A5<br>β-Chloro-L-alanine hydrochloride<br>1	A6<br>β-Chloro-L-alanine hydrochloride<br>2	A7<br>β-Chloro-L-alanine hydrochloride<br>3	A8<br>β-Chloro-L-alanine hydrochloride<br>4	A9<br>Thiosalicylic acid<br>1	A10<br>Thiosalicylic acid<br>2	A11<br>Thiosalicylic acid<br>3	A12<br>Thiosalicylic acid<br>4
B1<br>Sodium salicylate<br>1	B2<br>Sodium salicylate<br>2	B3<br>Sodium salicylate<br>3	B4<br>Sodium salicylate<br>4	B5<br>Hygromycin B<br>1	B6<br>Hygromycin B<br>2	B7<br>Hygromycin B<br>3	B8<br>Hygromycin B<br>4	B9<br>Ethionamide<br>1	B10<br>Ethionamide<br>2	B11<br>Ethionamide<br>3	B12<br>Ethionamide<br>4
C1<br>4-Aminopyridine<br>1	C2<br>4-Aminopyridine<br>2	C3<br>4-Aminopyridine<br>3	C4<br>4-Aminopyridine<br>4	C5<br>Sulfachloro-pyridazine<br>1	C6<br>Sulfachloro-pyridazine<br>2	C7<br>Sulfachloro-pyridazine<br>3	C8<br>Sulfachloro-pyridazine<br>4	C9<br>Sulfamono-methoxine<br>1	C10<br>Sulfamono-methoxine<br>2	C11<br>Sulfamono-methoxine<br>3	C12<br>Sulfamono-methoxine<br>4
D1<br>Oxycarboxin<br>1	D2<br>Oxycarboxin<br>2	D3<br>Oxycarboxin<br>3	D4<br>Oxycarboxin<br>4	D5<br>3-Amino-1,2,4-triazole<br>1	D6<br>3-Amino-1,2,4-triazole<br>2	D7<br>3-Amino-1,2,4-triazole<br>3	D8<br>3-Amino-1,2,4-triazole<br>4	D9<br>Chlorpromazine<br>1	D10<br>Chlorpromazine<br>2	D11<br>Chlorpromazine<br>3	D12<br>Chlorpromazine<br>4
E1<br>Niaproof<br>1	E2<br>Niaproof<br>2	E3<br>Niaproof<br>3	E4<br>Niaproof<br>4	E5<br>Compound 48/80<br>1	E6<br>Compound 48/80<br>2	E7<br>Compound 48/80<br>3	E8<br>Compound 48/80<br>4	E9<br>Sodium tungstate<br>1	E10<br>Sodium tungstate<br>2	E11<br>Sodium tungstate<br>3	E12<br>Sodium tungstate<br>4
F1<br>Lithium chloride<br>1	F2<br>Lithium chloride<br>2	F3<br>Lithium chloride<br>3	F4<br>Lithium chloride<br>4	F5<br>DL-Methionine hydroxamate<br>1	F6<br>DL-Methionine hydroxamate<br>2	F7<br>DL-Methionine hydroxamate<br>3	F8<br>DL-Methionine hydroxamate<br>4	F9<br>Tannic acid<br>1	F10<br>Tannic acid<br>2	F11<br>Tannic acid<br>3	F12<br>Tannic acid<br>4
G1<br>Chlorambucil<br>1	G2<br>Chlorambucil<br>2	G3<br>Chlorambucil<br>3	G4<br>Chlorambucil<br>4	G5<br>Cefamandole nafate<br>1	G6<br>Cefamandole nafate<br>2	G7<br>Cefamandole nafate<br>3	G8<br>Cefamandole nafate<br>4	G9<br>Cefoperazone<br>1	G10<br>Cefoperazone<br>2	G11<br>Cefoperazone<br>3	G12<br>Cefoperazone<br>4
H1<br>Cefsulodin<br>1	H2<br>Cefsulodin<br>2	H3<br>Cefsulodin<br>3	H4<br>Cefsulodin<br>4	H5<br>Caffeine<br>1	H6<br>Caffeine<br>2	H7<br>Caffeine<br>3	H8<br>Caffeine<br>4	H9<br>Phenylarsine oxide<br>1	H10<br>Phenylarsine oxide<br>2	H11<br>Phenylarsine oxide<br>3	H12<br>Phenylarsine oxide<br>4

PM18C MicroPlate™

A1<br>Ketoprofen<br>1	A2<br>Ketoprofen<br>2	A3<br>Ketoprofen<br>3	A4<br>Ketoprofen<br>4	A5<br>Sodium pyrophosphate decahydrate<br>1	A6<br>Sodium pyrophosphate decahydrate<br>2	A7<br>Sodium pyrophosphate decahydrate<br>3	A8<br>Sodium pyrophosphate decahydrate<br>4	A9<br>Thiamphenicol<br>1	A10<br>Thiamphenicol<br>2	A11<br>Thiamphenicol<br>3	A12<br>Thiamphenicol<br>4
B1<br>Trifluorothymidin e<br>1	B2<br>Trifluorothymidin e<br>2	B3<br>Trifluorothymidin e<br>3	B4<br>Trifluorothymidin e<br>4	B5<br>Pipemidic Acid<br>1	B6<br>Pipemidic Acid<br>2	B7<br>Pipemidic Acid<br>3	B8<br>Pipemidic Acid<br>4	B9<br>Azathioprine<br>1	B10<br>Azathioprine<br>2	B11<br>Azathioprine<br>3	B12<br>Azathioprine<br>4
C1<br>Poly-L-lysine<br>1	C2<br>Poly-L-lysine<br>2	C3<br>Poly-L-lysine<br>3	C4<br>Poly-L-lysine<br>4	C5<br>Sulfisoxazole<br>1	C6<br>Sulfisoxazole<br>2	C7<br>Sulfisoxazole<br>3	C8<br>Sulfisoxazole<br>4	C9<br>Pentachloro-phenol<br>1	C10<br>Pentachloro-phenol<br>2	C11<br>Pentachloro-phenol<br>3	C12<br>Pentachloro-phenol<br>4
D1<br>Sodium m-arsenite<br>1	D2<br>Sodium m-arsenite<br>2	D3<br>Sodium m-arsenite<br>3	D4<br>Sodium m-arsenite<br>4	D5<br>Sodium bromate<br>1	D6<br>Sodium bromate<br>2	D7<br>Sodium bromate<br>3	D8<br>Sodium bromate<br>4	D9<br>Lidocane<br>1	D10<br>Lidocane<br>2	D11<br>Lidocane<br>3	D12<br>Lidocane<br>4
E1<br>Sodium metasilicate<br>1	E2<br>Sodium metasilicate<br>2	E3<br>Sodium metasilicate<br>3	E4<br>Sodium metasilicate<br>4	E5<br>Sodium m-periodate<br>1	E6<br>Sodium m-periodate<br>2	E7<br>Sodium m-periodate<br>3	E8<br>Sodium m-periodate<br>4	E9<br>Antimony (III) chloride<br>1	E10<br>Antimony (III) chloride<br>2	E11<br>Antimony (III) chloride<br>3	E12<br>Antimony (III) chloride<br>4
F1<br>Semicarbazide<br>1	F2<br>Semicarbazide<br>2	F3<br>Semicarbazide<br>3	F4<br>Semicarbazide<br>4	F5<br>Tinidazole<br>1	F6<br>Tinidazole<br>2	F7<br>Tinidazole<br>3	F8<br>Tinidazole<br>4	F9<br>Aztreonam<br>1	F10<br>Aztreonam<br>2	F11<br>Aztreonam<br>3	F12<br>Aztreonam<br>4
G1<br>Triclosan<br>1	G2<br>Triclosan<br>2	G3<br>Triclosan<br>3	G4<br>Triclosan<br>4	G5<br>3,5-Diamino-1,2,4-triazole (Guanazole)<br>1	G6<br>3,5-Diamino-1,2,4-triazole (Guanazole)<br>2	G7<br>3,5-Diamino-1,2,4-triazole (Guanazole)<br>3	G8<br>3,5-Diamino-1,2,4-triazole (Guanazole)<br>4	G9<br>Myricetin<br>1	G10<br>Myricetin<br>2	G11<br>Myricetin<br>3	G12<br>Myricetin<br>4
H1<br>5-fluoro-5'-deoxyuridine<br>1	H2<br>5-fluoro-5'-deoxyuridine<br>2	H3<br>5-fluoro-5'-deoxyuridine<br>3	H4<br>5-fluoro-5'-deoxyuridine<br>4	H5<br>2-Phenylphenol<br>1	H6<br>2-Phenylphenol<br>2	H7<br>2-Phenylphenol<br>3	H8<br>2-Phenylphenol<br>4	H9<br>Plumbagin<br>1	H10<br>Plumbagin<br>2	H11<br>Plumbagin<br>3	H12<br>Plumbagin<br>4

PM17 (Chemical Sensitivity Bacteria)

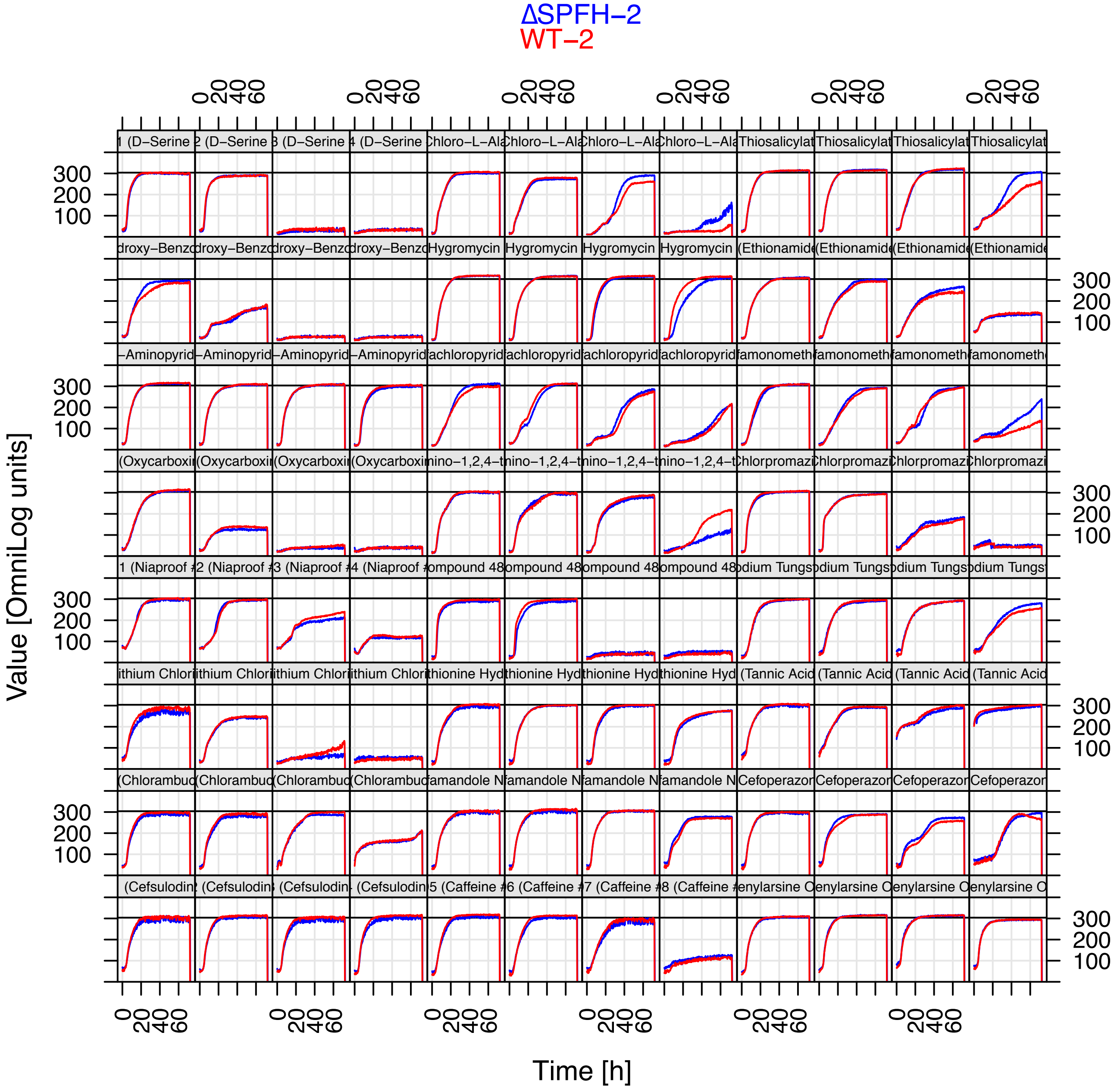

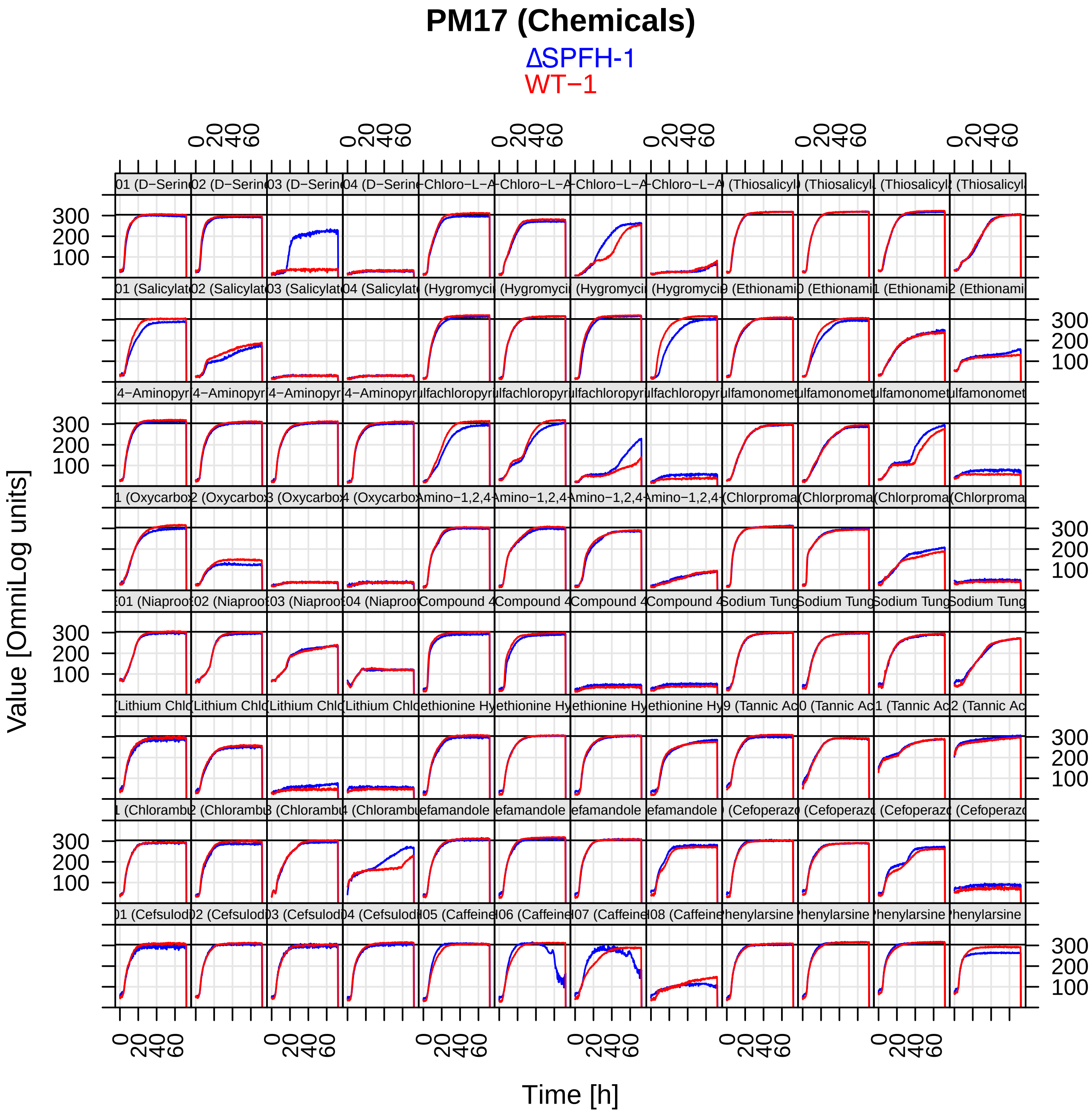
PM17 (Chemicals)
ΔSPFH-1
WT−1
Value [OmniLog units]
Time [h]

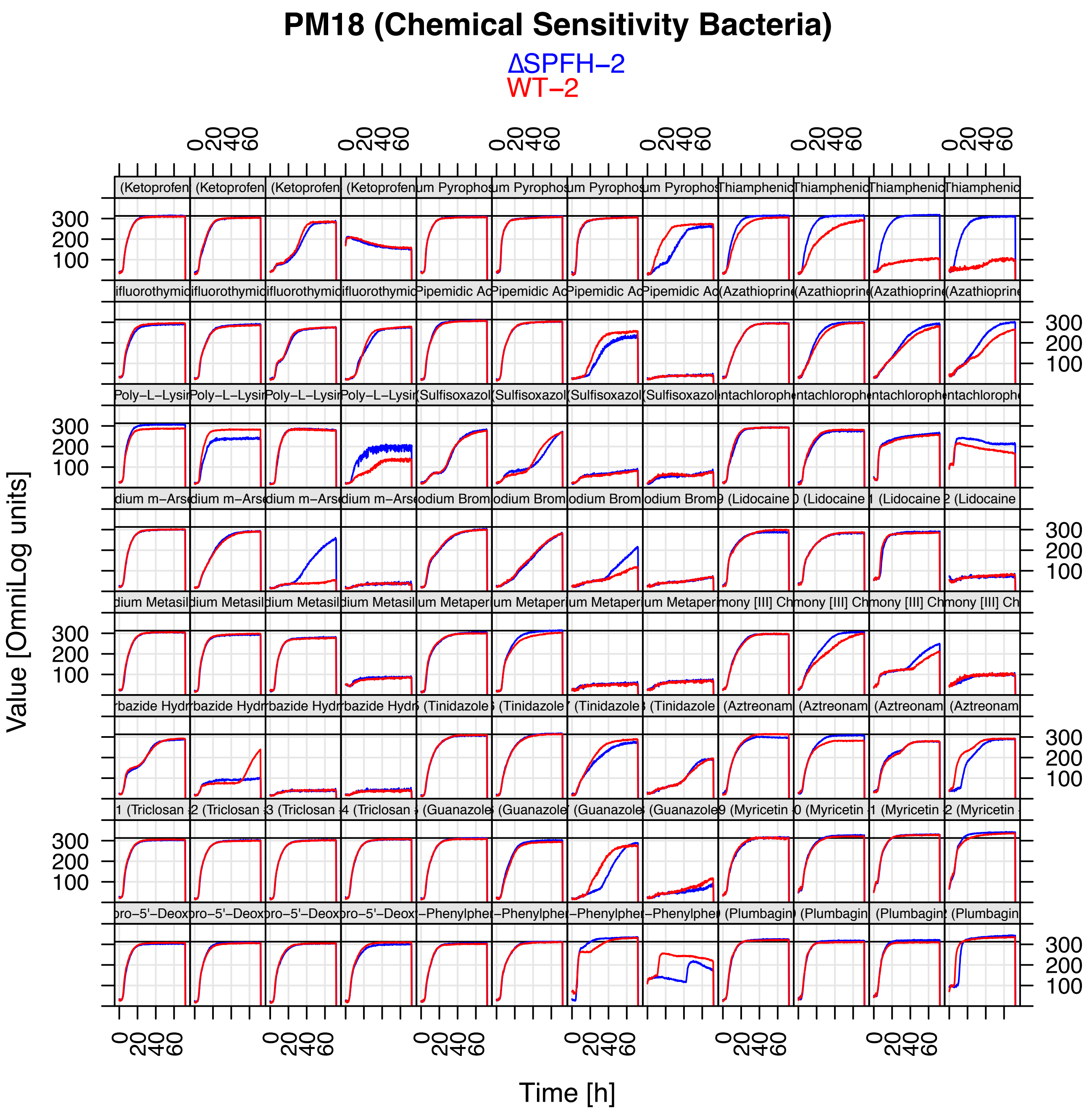
PM18 (Chemical Sensitivity Bacteria)
ΔSPFH−2
WT−2
Value [OmniLog units]
Time [h]

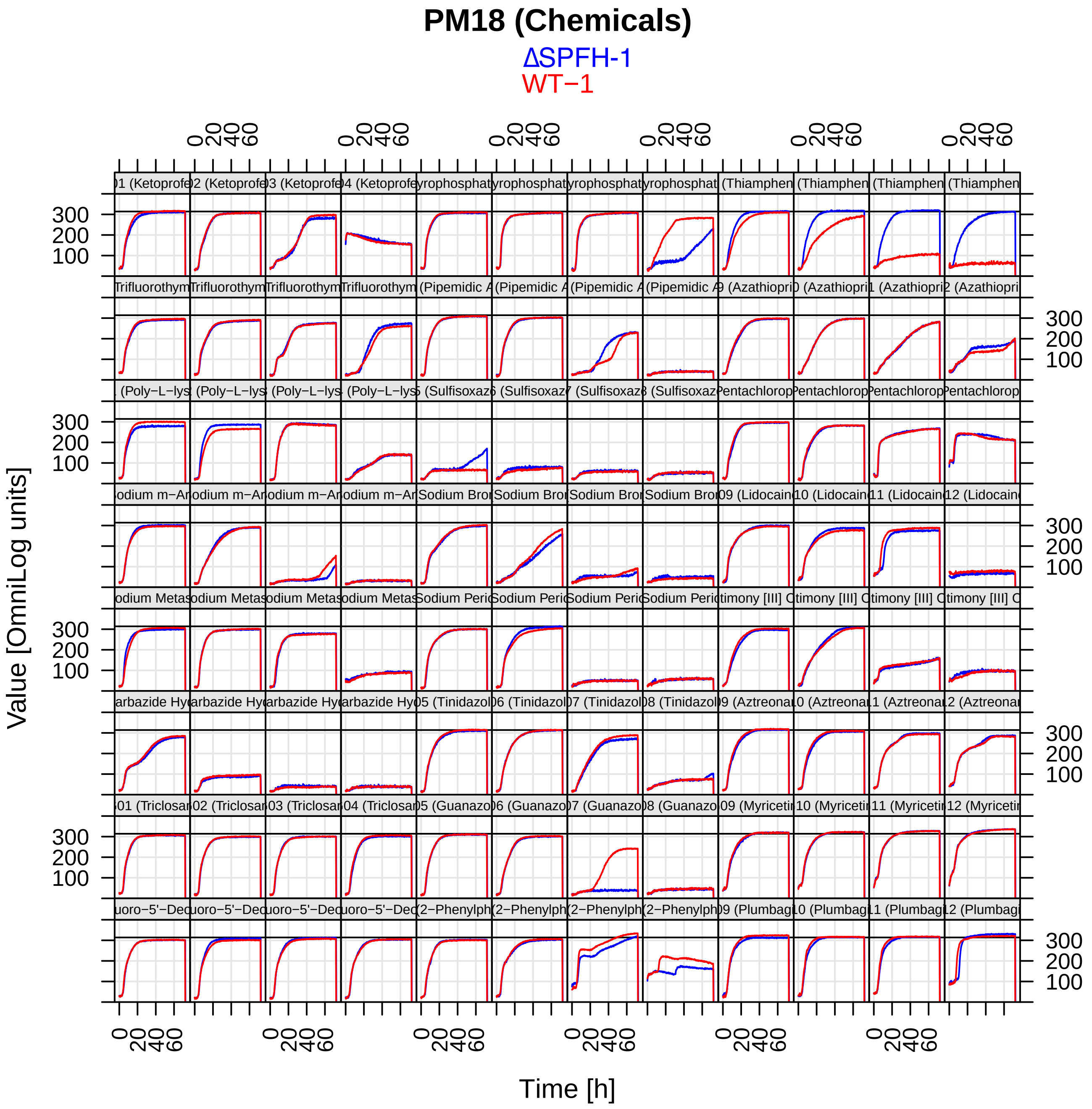
PM18 (Chemicals)
ΔSPFH-1
WT−1
Value [OmniLog units]
Time [h]

PM19 MicroPlate™

A1<br>Josamycin<br>1	A2<br>Josamycin<br>2	A3<br>Josamycin<br>3	A4<br>Josamycin<br>4	A5<br>Gallic acid<br>1	A6<br>Gallic acid<br>2	A7<br>Gallic acid<br>3	A8<br>Gallic acid<br>4	A9<br>Coumarin<br>1	A10<br>Coumarin<br>2	A11<br>Coumarin<br>3	A12<br>Coumarin<br>4
B1<br>Methyltrioctyl-ammonium chloride<br>1	B2<br>Methyltrioctyl-ammonium chloride<br>2	B3<br>Methyltrioctyl-ammonium chloride<br>3	B4<br>Methyltrioctyl-ammonium chloride<br>4	B5<br>Harmane<br>1	B6<br>Harmane<br>2	B7<br>Harmane<br>3	B8<br>Harmane<br>4	B9<br>2,4-Dintrophenol<br>1	B10<br>2,4-Dintrophenol<br>2	B11<br>2,4-Dintrophenol<br>3	B12<br>2,4-Dintrophenol<br>4
C1<br>Chlorhexidine<br>1	C2<br>Chlorhexidine<br>2	C3<br>Chlorhexidine<br>3	C4<br>Chlorhexidine<br>4	C5<br>Umbelliferone<br>1	C6<br>Umbelliferone<br>2	C7<br>Umbelliferone<br>3	C8<br>Umbelliferone<br>4	C9<br>Cinnamic acid<br>1	C10<br>Cinnamic acid<br>2	C11<br>Cinnamic acid<br>3	C12<br>Cinnamic acid<br>4
D1<br>Disulphiram<br>1	D2<br>Disulphiram<br>2	D3<br>Disulphiram<br>3	D4<br>Disulphiram<br>4	D5<br>Iodonitro Tetrazolium Violet<br>1	D6<br>Iodonitro Tetrazolium Violet<br>2	D7<br>Iodonitro Tetrazolium Violet<br>3	D8<br>Iodonitro Tetrazolium Violet<br>4	D9<br>Phenyl- methyl-sulfonyl-fluoride (PMSF)<br>1	D10<br>Phenyl- methyl-sulfonyl-fluoride (PMSF)<br>2	D11<br>Phenyl- methyl-sulfonyl-fluoride (PMSF)<br>3	D12<br>Phenyl- methyl-sulfonyl-fluoride (PMSF)<br>4
E1<br>FCCP<br>1	E2<br>FCCP<br>2	E3<br>FCCP<br>3	E4<br>FCCP<br>4	E5<br>D,L-Thioctic Acid<br>1	E6<br>D,L-Thioctic Acid<br>2	E7<br>D,L-Thioctic Acid<br>3	E8<br>D,L-Thioctic Acid<br>4	E9<br>Lawsone<br>1	E10<br>Lawsone<br>2	E11<br>Lawsone<br>3	E12<br>Lawsone<br>4
F1<br>Phenethicillin<br>1	F2<br>Phenethicillin<br>2	F3<br>Phenethicillin<br>3	F4<br>Phenethicillin<br>4	F5<br>Blasticidin S<br>1	F6<br>Blasticidin S<br>2	F7<br>Blasticidin S<br>3	F8<br>Blasticidin S<br>4	F9<br>Sodium caprylate<br>1	F10<br>Sodium caprylate<br>2	F11<br>Sodium caprylate<br>3	F12<br>Sodium caprylate<br>4
G1<br>Lauryl sulfobetaine<br>1	G2<br>Lauryl sulfobetaine<br>2	G3<br>Lauryl sulfobetaine<br>3	G4<br>Lauryl sulfobetaine<br>4	G5<br>Dihydro-streptomycin<br>1	G6<br>Dihydro-streptomycin<br>2	G7<br>Dihydro-streptomycin<br>3	G8<br>Dihydro-streptomycin<br>4	G9<br>Hydroxylamine<br>1	G10<br>Hydroxylamine<br>2	G11<br>Hydroxylamine<br>3	G12<br>Hydroxylamine<br>4
H1<br>Hexammine cobalt (III) chloride<br>1	H2<br>Hexammine cobalt (III) chloride<br>2	H3<br>Hexammine cobalt (III) chloride<br>3	H4<br>Hexammine cobalt (III) chloride<br>4	H5<br>Thioglycerol<br>1	H6<br>Thioglycerol<br>2	H7<br>Thioglycerol<br>3	H8<br>Thioglycerol<br>4	H9<br>Polymyxin B<br>1	H10<br>Polymyxin B<br>2	H11<br>Polymyxin B<br>3	H12<br>Polymyxin B<br>4

PM20B MicroPlate™

A1<br>Amitriptyline<br>1	A2<br>Amitriptyline<br>2	A3<br>Amitriptyline<br>3	A4<br>Amitriptyline<br>4	A5<br>Apramycin<br>1	A6<br>Apramycin<br>2	A7<br>Apramycin<br>3	A8<br>Apramycin<br>4	A9<br>Benserazide<br>1	A10<br>Benserazide<br>2	A11<br>Benserazide<br>3	A12<br>Benserazide<br>4
B1<br>Orphenadrine<br>1	B2<br>Orphenadrine<br>2	B3<br>Orphenadrine<br>3	B4<br>Orphenadrine<br>4	B5<br>D,L-Propranolol<br>1	B6<br>D,L-Propranolol<br>2	B7<br>D,L-Propranolol<br>3	B8<br>D,L-Propranolol<br>4	B9<br>Tetrazolium violet<br>1	B10<br>Tetrazolium violet<br>2	B11<br>Tetrazolium violet<br>3	B12<br>Tetrazolium violet<br>4
C1<br>Thioridazine<br>1	C2<br>Thioridazine<br>2	C3<br>Thioridazine<br>3	C4<br>Thioridazine<br>4	C5<br>Atropine<br>1	C6<br>Atropine<br>2	C7<br>Atropine<br>3	C8<br>Atropine<br>4	C9<br>Ornidazole<br>1	C10<br>Ornidazole<br>2	C11<br>Ornidazole<br>3	C12<br>Ornidazole<br>4
D1<br>Proflavine<br>1	D2<br>Proflavine<br>2	D3<br>Proflavine<br>3	D4<br>Proflavine<br>4	D5<br>Ciprofloxacin<br>1	D6<br>Ciprofloxacin<br>2	D7<br>Ciprofloxacin<br>3	D8<br>Ciprofloxacin<br>4	D9<br>18-Crown-6 ether<br>1	D10<br>18-Crown-6 ether<br>2	D11<br>18-Crown-6 ether<br>3	D12<br>18-Crown-6 ether<br>4
E1<br>Crystal violet<br>1	E2<br>Crystal violet<br>2	E3<br>Crystal violet<br>3	E4<br>Crystal violet<br>4	E5<br>Dodine<br>1	E6<br>Dodine<br>2	E7<br>Dodine<br>3	E8<br>Dodine<br>4	E9<br>Hexa-chlorophene<br>1	E10<br>Hexa-chlorophene<br>2	E11<br>Hexa-chlorophene<br>3	E12<br>Hexa-chlorophene<br>4
F1<br>4-Hydroxy-coumarin<br>1	F2<br>4-Hydroxy-coumarin<br>2	F3<br>4-Hydroxy-coumarin<br>3	F4<br>4-Hydroxy-coumarin<br>4	F5<br>Oxytetracycline<br>1	F6<br>Oxytetracycline<br>2	F7<br>Oxytetracycline<br>3	F8<br>Oxytetracycline<br>4	F9<br>Pridinol<br>1	F10<br>Pridinol<br>2	F11<br>Pridinol<br>3	F12<br>Pridinol<br>4
G1<br>Captan<br>1	G2<br>Captan<br>2	G3<br>Captan<br>3	G4<br>Captan<br>4	G5<br>3,5-Dinitro-benzene<br>1	G6<br>3,5-Dinitro-benzene<br>2	G7<br>3,5-Dinitro-benzene<br>3	G8<br>3,5-Dinitro-benzene<br>4	G9<br>8-Hydroxy-quinoline<br>1	G10<br>8-Hydroxy-quinoline<br>2	G11<br>8-Hydroxy-quinoline<br>3	G12<br>8-Hydroxy-quinoline<br>4
H1<br>Patulin<br>1	H2<br>Patulin<br>2	H3<br>Patulin<br>3	H4<br>Patulin<br>4	H5<br>Tolylfluanid<br>1	H6<br>Tolylfluanid<br>2	H7<br>Tolylfluanid<br>3	H8<br>Tolylfluanid<br>4	H9<br>Troleandomycin<br>1	H10<br>Troleandomycin<br>2	H11<br>Troleandomycin<br>3	H12<br>Troleandomycin<br>4

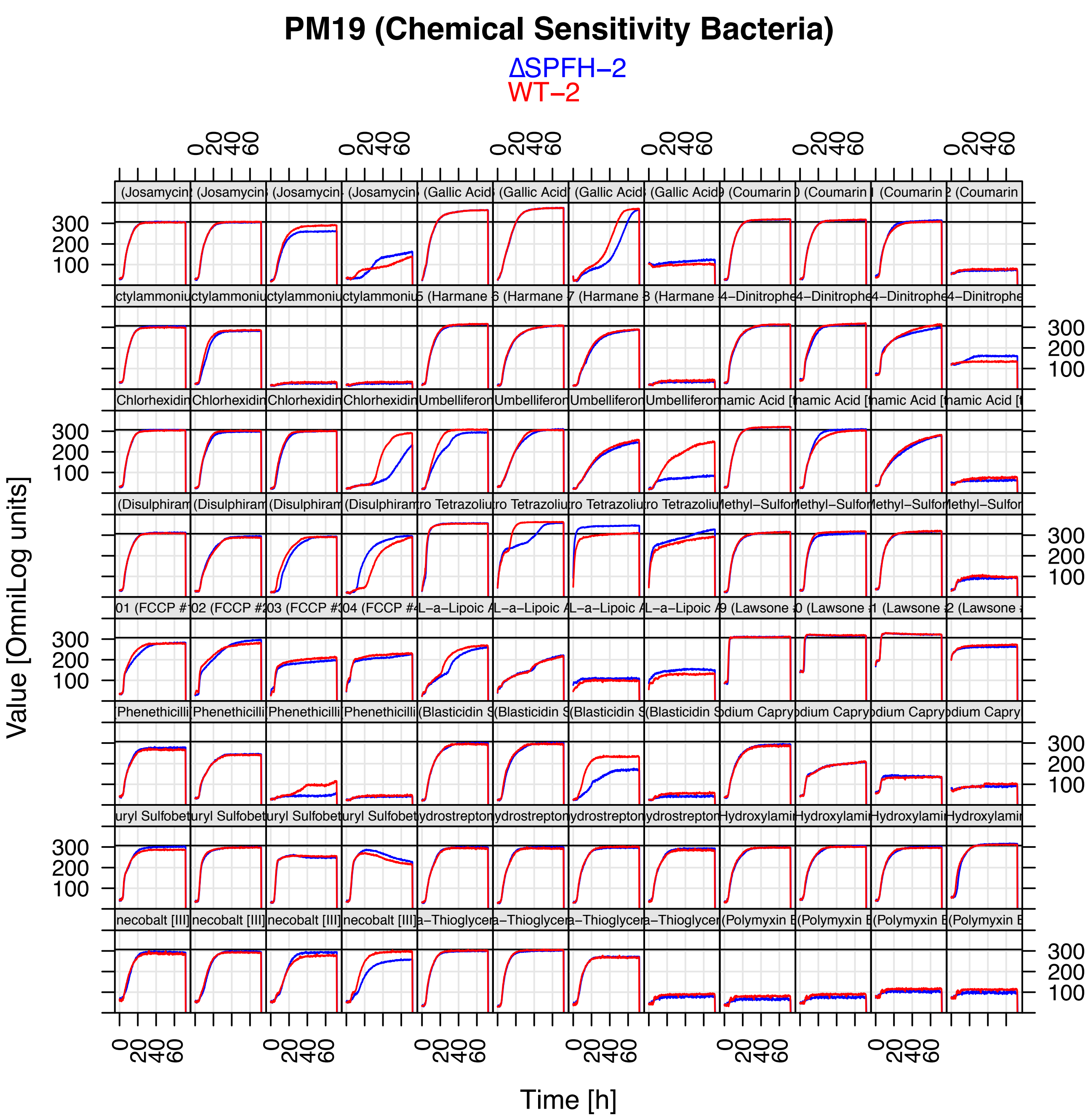

PM19 (Chemical Sensitivity Bacteria)
ΔSPFH−2
WT−2
(Josamycin
(Josamycin
(Josamycin
(Josamycin
(Gallic Acid
(Gallic Acid
(Gallic Acid
(Gallic Acid
(Coumarin
(Coumarin
(Coumarin
(Coumarin
ctylammoniu
ctylammoniu
ctylammoniu
ctylammoniu
(Harmane
(Harmane
(Harmane
(Harmane
4−Dinitrophe
4−Dinitrophe
4−Dinitrophe
4−Dinitrophe
Chlorhexidin
Chlorhexidin
Chlorhexidin
Chlorhexidin
Umbelliferon
Umbelliferon
Umbelliferon
Umbelliferon
namic Acid [
namic Acid [
namic Acid [
namic Acid [
(Disulphiram
(Disulphiram
(Disulphiram
(Disulphiram
ro Tetrazoliu
ro Tetrazoliu
ro Tetrazoliu
ro Tetrazoliu
lethyl−Sulfor
lethyl−Sulfor
lethyl−Sulfor
lethyl−Sulfor
01 (FCCP #1
02 (FCCP #2
03 (FCCP #3
04 (FCCP #4
L−a−Lipoic A
L−a−Lipoic A
L−a−Lipoic A
L−a−Lipoic A
(Lawsone
(Lawsone
(Lawsone
(Lawsone
Phenethicilli
Phenethicilli
Phenethicilli
Phenethicilli
(Blasticidin S
(Blasticidin S
(Blasticidin S
(Blasticidin S
dium Capry
dium Capry
dium Capry
dium Capry
uryl Sulfobet
uryl Sulfobet
uryl Sulfobet
uryl Sulfobet
ydrostrepton
ydrostrepton
ydrostrepton
ydrostrepton
Hydroxylami
Hydroxylami
Hydroxylami
Hydroxylami
necobalt [III]
necobalt [III]
necobalt [III]
necobalt [III]
a−Thioglycer
a−Thioglycer
a−Thioglycer
a−Thioglycer
(Polymyxin E
(Polymyxin E
(Polymyxin E
(Polymyxin E
Value [OmniLog units]
100
200
300
0
20
40
60
Time [h]

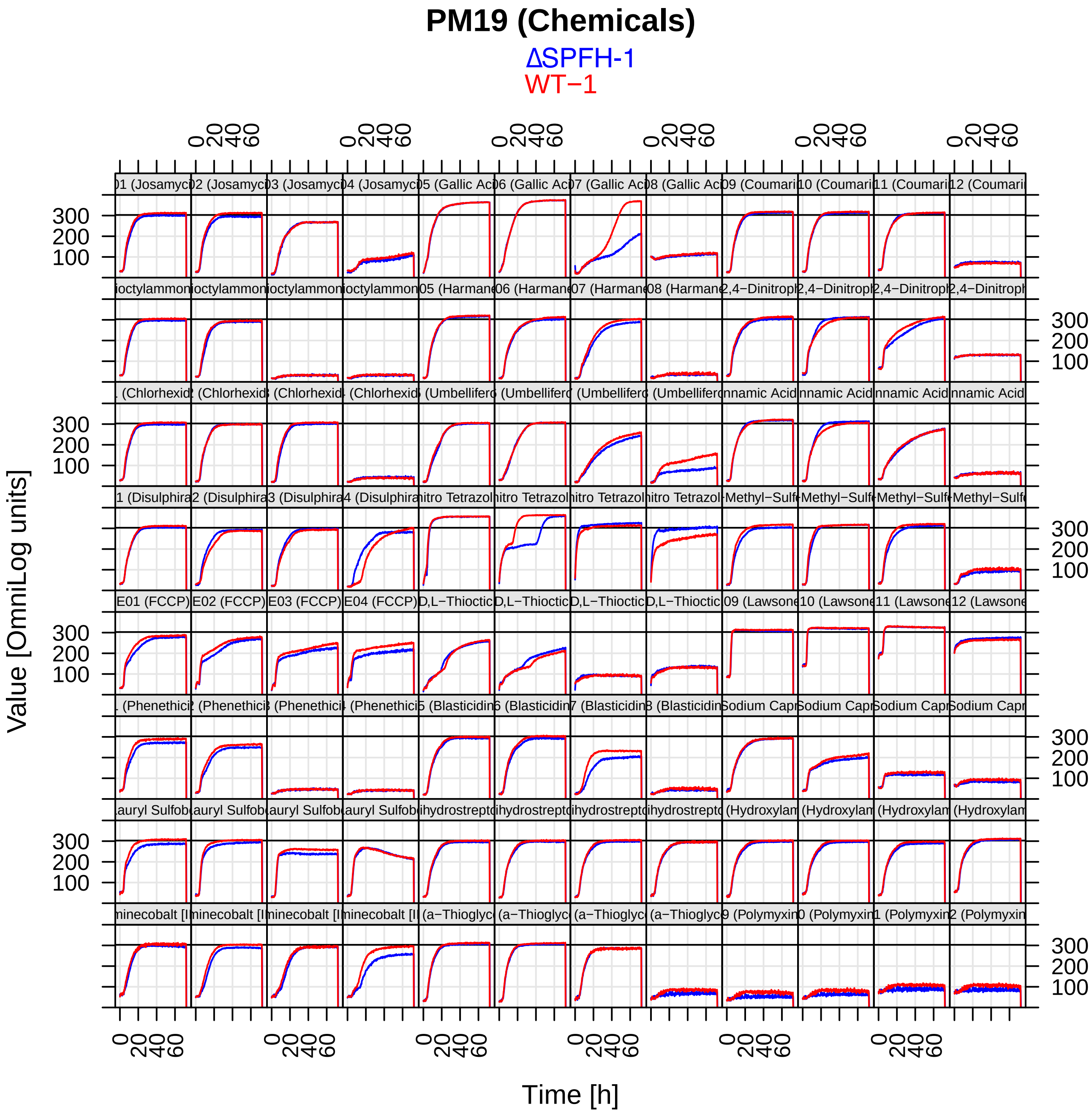
PM19 (Chemicals)
ΔSPFH-1
WT−1
Value [OmniLog units]
Time [h]

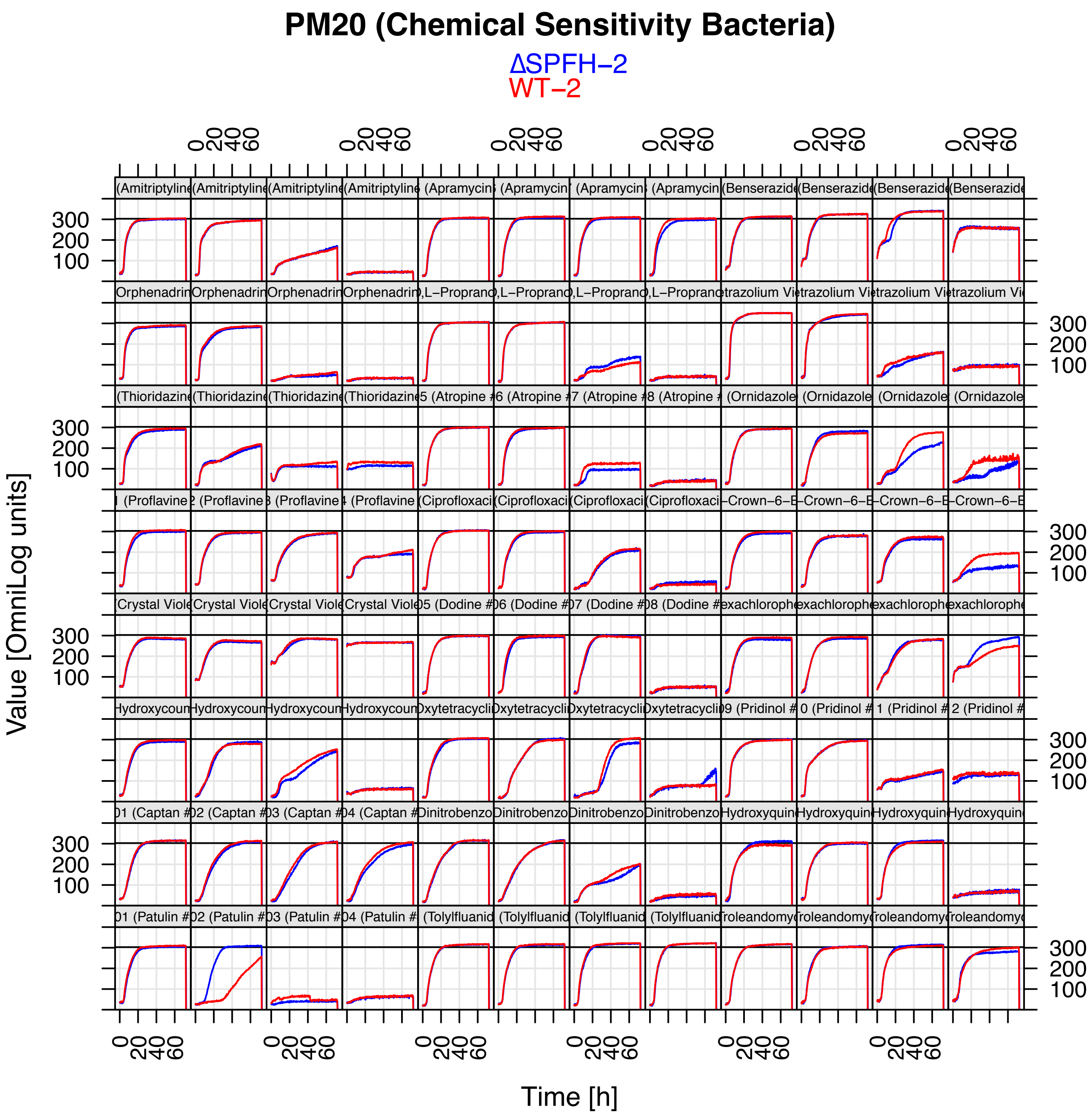
PM20 (Chemical Sensitivity Bacteria)
ΔSPFH−2
WT−2
Value [OmniLog units]
Time [h]

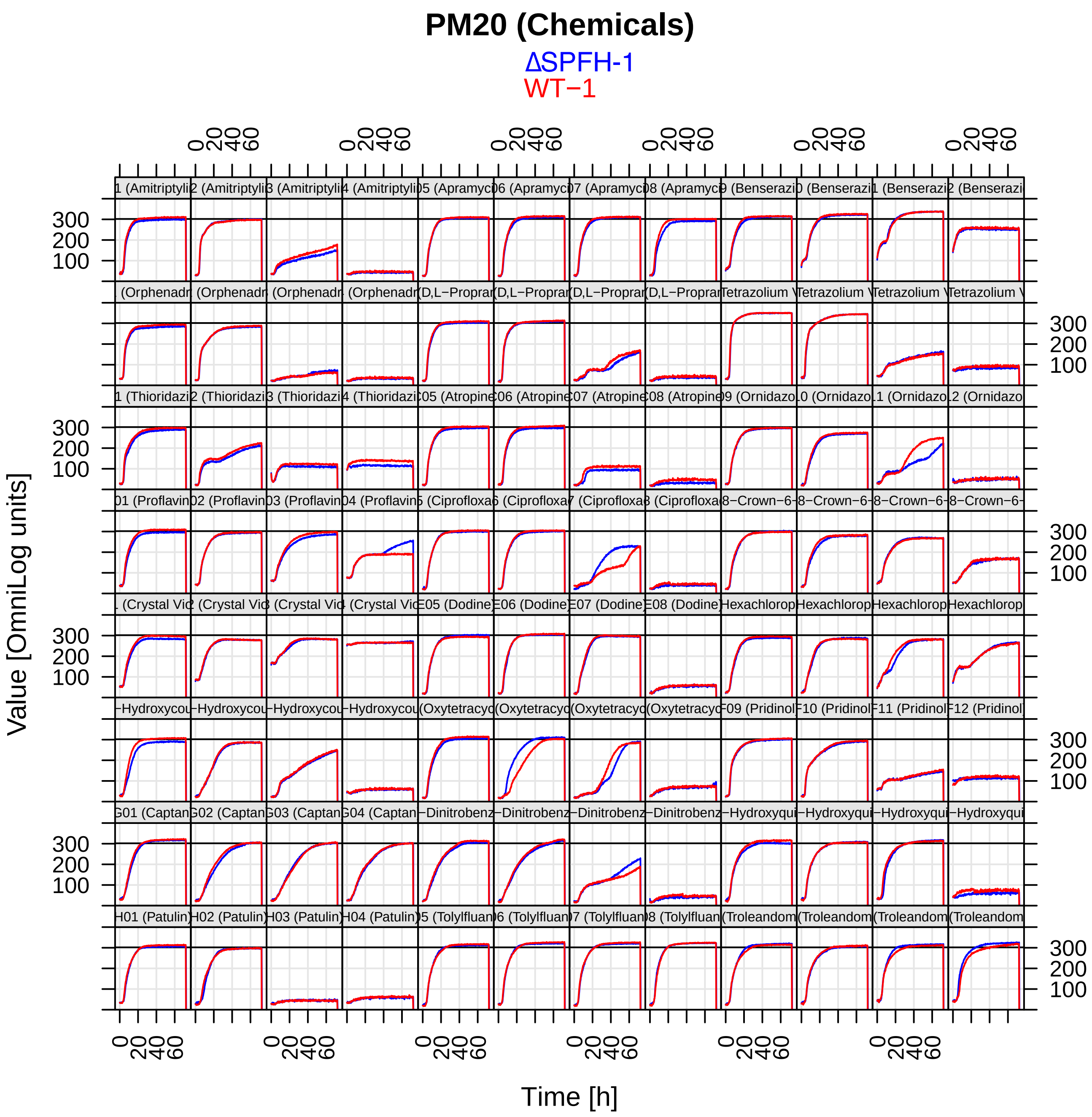
PM20 (Chemicals)
ΔSPFH-1
WT−1
1 (Amitriptyli
2 (Amitriptyli
3 (Amitriptyli
4 (Amitriptyli
05 (Apramyc
06 (Apramyc
07 (Apramyc
08 (Apramyc
9 (Benserazi
0 (Benserazi
1 (Benserazi
2 (Benserazi
(Orphenadr
(Orphenadr
(Orphenadr
(Orphenadr
(D,L−Propran
(D,L−Propran
(D,L−Propran
(D,L−Propran
Tetrazolium V
Tetrazolium V
Tetrazolium V
Tetrazolium V
1 (Thioridazi
2 (Thioridazi
3 (Thioridazi
4 (Thioridazi
C05 (Atropine
C06 (Atropine
C07 (Atropine
C08 (Atropine
9 (Ornidazo
0 (Ornidazo
1 (Ornidazo
2 (Ornidazo
01 (Proflavin
02 (Proflavin
03 (Proflavin
04 (Proflavin
5 (Ciprofloxa
6 (Ciprofloxa
7 (Ciprofloxa
8 (Ciprofloxa
8−Crown−6−
8−Crown−6−
8−Crown−6−
8−Crown−6−
(Crystal Vio
2 (Crystal Vio
3 (Crystal Vio
4 (Crystal Vio
E05 (Dodine
E06 (Dodine
E07 (Dodine
E08 (Dodine
Hexachlorop
Hexachlorop
Hexachlorop
Hexachlorop
−Hydroxycou
−Hydroxycou
−Hydroxycou
−Hydroxycou
(Oxytetracyc
(Oxytetracyc
(Oxytetracyc
(Oxytetracyc
F09 (Pridinol
F10 (Pridinol
F11 (Pridinol
F12 (Pridinol
G01 (Captan
G02 (Captan
G03 (Captan
G04 (Captan
−Dinitrobenz
−Dinitrobenz
−Dinitrobenz
−Dinitrobenz
−Hydroxyqui
−Hydroxyqui
−Hydroxyqui
−Hydroxyqui
H01 (Patulin)
H02 (Patulin)
H03 (Patulin)
H04 (Patulin)
5 (Tolylfluan
6 (Tolylfluan
7 (Tolylfluan
8 (Tolylfluan
(Troleandom
(Troleandom
(Troleandom
(Troleandom
100
200
300
0
20
40
60
Value [OmniLog units]
Time [h]
